# Supplementary material for: Environmental damping and vibrational coupling of confined fluids within isolated carbon nanotubes
Source: Nat Commun. 2024 Jul 3;15:5605. doi: 10.1038/s41467-024-49661-8 (PMC11222464; doi:10.1038/s41467-024-49661-8)
Supplement: Supplementary file 1 — Supplementary Information [file 41467_2024_49661_MOESM1_ESM.pdf]

Supplementary Information

Environmental Damping and Vibrational Coupling of Confined Fluids within Isolated Carbon Nanotubes

Yu-Ming Tu[1,†], Matthias Kuehne[1,2,†], Rahul Prasanna Misra[1], Cody L. Ritt[1], Hananeh Oliaei[3], Samuel Faucher[1], Haokun Li[4], Xintong Xu[4], Aubrey Penn[5], Sungyun Yang[1], Jing Fan Yang[1], Kyle Sendgikoski[6], Joshika Chakraverty[1], John Cumings[7], Arun Majumdar[4,8], Narayana Aluru[9], Jordan A. Hachtel[10], Daniel Blankschtein[1], and Michael S. Strano[1,*]

Affiliations:

1. Department of Chemical Engineering, Massachusetts Institute of Technology, Cambridge, MA 02139, USA
2. Department of Physics, Brown University, Providence, RI 02912, USA
3. Department of Mechanical Science and Engineering, University of Illinois Urbana-Champaign, Urbana, IL 61801, USA
4. Department of Mechanical Engineering, Stanford University, Stanford, CA, 94305, USA
5. MIT.nano, Massachusetts Institute of Technology, Cambridge, MA 02139, USA
6. Department of Physics, University of Maryland, College Park, MD 20742, USA
7. Department of Materials Science and Engineering, University of Maryland, College Park, MD 20742, USA
8. Stanford Precourt Institute for Energy, Stanford, CA, 94305, USA
9. Department of Mechanical Engineering, Oden Institute for Computational Engineering and Sciences, University of Texas at Austin, Austin, TX 78712, USA

10. Center for Nanophase Materials Sciences, Oak Ridge National Laboratory, Oak Ridge, TN 37831, USA

*Corresponding author. Email: strano@mit.edu
†These authors contributed equally to this work.

The PDF file includes the following:

Supplementary Text 1 to 14

Supplementary Fig.

Supplementary Movie 1 and 2

Supplementary Table

Supplementary References

Table of Contents

Supplementary Text **1**

1: Other Sources of Environmental Coupling for the Radial Breathing Mode (RBM) **1**

a) Coupling to the CNT substrate 1

b) Intrinsic temperature invariance of the RBM frequency 2

2: Summary of Full Raman Spectra for CNT G, X, and F **3**

a) CNT G full Raman spectra 4

b) CNT X full Raman spectra 8

c) CNT F Full Raman spectra 16

3: Summary of All RBM Trajectories and Derivative Analysis for CNT G, X, and F **27**

a) CNT G RBM Thermal Trajectories and Derivative Analysis 27

b) CNT X RBM Thermal Trajectories and Derivative Analysis 29

c) CNT F RBM Thermal Trajectories and Derivative Analysis 34

d) Rare Trajectories with Two Apparent $\boldsymbol{T}\mathbf{max}$ Values 37

4: Summary of $\boldsymbol{T}\mathbf{max}$ Analysis of All RBM Trajectories for CNT G, X, and F **38**

a) $\boldsymbol{T}\mathbf{max}$ values for each data file for CNT G 38

b) $\boldsymbol{T}\mathbf{max}$ values for each data file for CNT X 39

c) $\boldsymbol{T}\mathbf{max}$ values for each data file for CNT F 39

5: Transmission Electron Microscopy (TEM) of CNTs and Graphitic Impurities **40**

a) Statistical information on CNTs produced from the CVD synthesis of this work 40

b) TEM Video of Graphitic Ribbons on a CNT and Mean Squared Displacement 43

6: Thermodynamic and Mathematical Proof Distinguishing Damping and Fluid Adsorption **43**

a) Falsifying the null hypothesis of an adsorbed fluid changing the restoring force at the CNT wall or net spring constant ($\boldsymbol{\gamma}$), no damping term 44

b) The downward concavity of the RBM trajectory requires a negative 2nd derivative 46

7: Use of the Single Shell Approximation versus Double Coupled Shells **47**

8: Full Derivation of the RBM Frequency from the Harmonic Oscillator Model **50**

a) Single shell force balance: 50

b) A linear damping, increasing with temperature appears experimentally justified 50

c) Scaling Requirements with Temperature for the Spring Constant to Describe RBM Trajectories 52

9: The Magnitude of the RBM Trajectories is Consistent with Damping **52**

10: Strain-induced coupling and damping of the RBM oscillator **53**

11: Understanding the Limiting $\boldsymbol{\omega}$RBM when $\boldsymbol{T > T}\mathbf{max}$ **55**

a) Derivation of the limiting $\boldsymbol{\omega}\mathbf{RBM}$ when $\boldsymbol{T} > \boldsymbol{T}\mathbf{max}$ 56
b) Evaluation of the intercept in terms of approximate β: 58
12: RBM Peak Variance or Full Width at Half Maximum (FWHM) 59
a) The FWHM of the RBM peak tracks the RBM frequency for a given temperature scan 60
b) The minimum RBM variance across locations varies greater than between scans 60
c) Thermal annealing for 12 hrs systematically decreases the RBM variance 68
d) The RBM variance tracks the temperature dependent change in damping, $\boldsymbol{b}[\boldsymbol{T}]$ 70
13: Comparison with Prior Literature Measurements of the RBM 73
a) Comparison with Liu *et al.*[8] 73
b) Scaling $\boldsymbol{\gamma}\mathbf{CC}$ values for a series of DWNT 74
14: RBM Trajectories Change after FIB Cutting and Interior Water Filling 78
a) TEM Imaging of Interior Water is Statistically Rare 79
b) RBM Trajectories for Interior Filled CNTs Exhibit a Positive Second Derivative at High Temperature 79
c) Substrate-Supported Interior Filling CNTs Replicate a Positive Second Derivative 84
Supplementary References 86

Lists of Figures and Tables

Supplementary Fig. 1-1| Observations for the Radial Breathing Mode (RBM) shift of a substrate-bounded CNT. 1

Supplementary Fig. 1-2| No distinct RBM frequency shifts of three CNTs upon laser heating. 3

Supplementary Fig. 3-1| Three scans showing two $T\max$ values. 37

Supplementary Fig. 5-1| Transmission electron microscopy of as-grown CNTs on commercial TEM grids. 42

Supplementary Fig. 5-2| Representative TEM side-by-side still frame of graphitic ribbon motions on a CNT. 43

Supplementary Fig. 6-1| A representative plot of all 93 temperature scans. 44

Supplementary Fig. 7-1| Extension of the Single Shell Approximation to the Double Coupled Shell Model. 49

Supplementary Fig. 12-1| RBM frequency trajectories (top row) and corresponding FWHM trajectories (bottom row) for successive scans of CNT X at $2.5 \times 10 - 5$ bar. 60

Supplementary Fig. 12-2| Comparison of FWHM ($T > Tmax$) and $\omega RBM, min$ variance between locations and vacuum conditions. 69

Supplementary Fig. 12-3 | Selected scaled variances for the whole temperature scan versus scaled temperature-dependent damping constant of CNT X. 72

Supplementary Fig. 13-1| Comparison of experimental data with Lennard-Jones and Buckingham models. 77

Supplementary Fig. 14-1| Interior water filled CNT sample fabrication and TEM imaging. 78

Supplementary Fig. 14-2| 78 Raman spectra of CNT (Fig. 5e) scanned with a pressure of 4e-2mbar 81

Supplementary Fig. 14-3| Interior filled isobars of water vapor-filled CNTs. 82

Supplementary Fig. 14-4| Observation of reversible fluid isobar of water-immersed, FIB-cut opened CNT. 85

Supplementary Table 11-1| Comparison of uncoupled frequencies predicted by Eq. (8) and mechanical models **59**

Supplementary Table 12-1| Comparison of minimum RBM frequencies and FWHM variances for CNT X. **61**

Supplementary Table 12-2| Comparison of minimum RBM frequencies and FWHM variances for CNT F **64**

Supplementary Table 12-3| Comparison of minimum RBM frequencies and FWHM variances for CNT G. **67**

Supplementary Table 12-4| Comparison of minimum RBM frequencies and FWHM variances for CNT X before and after bakeout at 100 ^{o}C for 12 hrs. **68**

Supplementary Text

1: Other Sources of Environmental Coupling for the Radial Breathing Mode (RBM)

a) Coupling to the CNT substrate

To examine the substrate coupling effects of as-grown CNTs, we synthesized an ultralong (longer than a millimeter) CNT via the CVD method on a SiO_2 substrate. The CNTs were then immersed in water to determine the extra coupling effect of the introduced liquid. The statistical results presented below offer an overview observation of CNTs immersed in a water environment. We observed that immersing the substrate-bound CNT into liquid water results in only a 1 cm^{-1} upshift in RBM frequency, despite the fact that more than 80% of the external exposed surface is available for coupling to the newly introduced water (**Supplementary Fig. 1-1**). The overall statistical information of the same synthesis batch of substrate-supported CNTs in water indicates that 9 out of 10 CNTs show 0.5 or less cm^{-1} Raman upshift in water. In contrast, only 1 out of 10 CNTs exhibits >1 cm^{-1} upshift in RBM frequency. The small upshift in the RBM frequency suggests that the initial substrate coupling almost saturates the frequency response to the external surroundings.

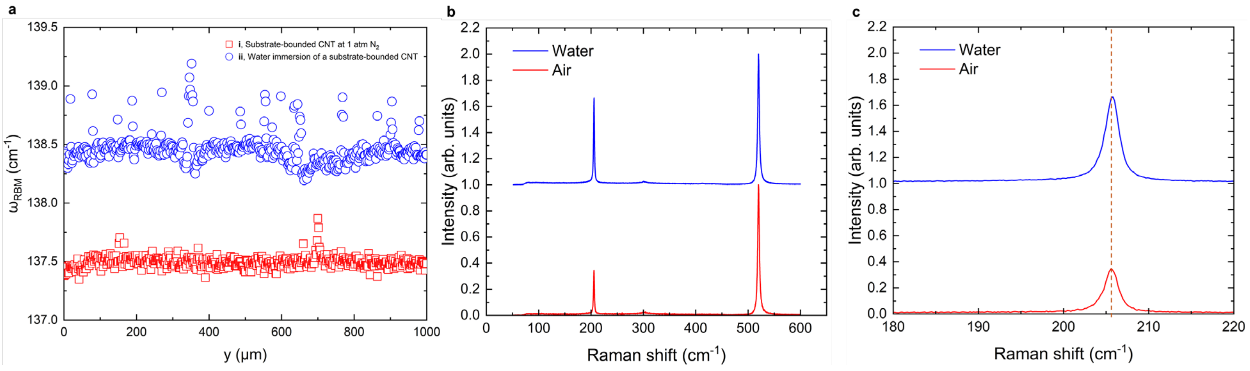

Supplementary Fig. 1-1| Observations for the Radial Breathing Mode (RBM) shift of a substrate-bounded CNT.
a, (**i**) Raman scans taken at 1 atm N_2 along the SiO_2 substrate-bounded, millimeter-long, as-synthesized CNT, with measurements every 2 μm shows that ω_{RBM} remains almost consistent at approximately 137.5 cm^{-1} over 1 mm long. (**ii**) When the substrate-bounded CNT is immersed in water, ω_{RBM} is at about 138.5 cm^{-1}, which is only an approximately 1 cm^{-1} upshift in RBM frequency. **b**, Comparison of representative Raman spectra of a substrate-

supported CNT in both atmosphere air and water. The RBM shift observed at approximately 300 cm^{-1} and 520 cm^{-1} are attributed to the silicon substrate, with the 520.7 cm^{-1} silicon peak specially used for spectrum calibration. These signals confirm the presence of the CNT on the silicon substrate. The ω_{RBM} of the CNT is measured at 205.6 cm^{-1} (in air) and 205.8 cm^{-1} (in water) of the CNT. **c**, The zoomed-in Raman spectrum illustrates the comparison of RBM frequencies. The orange dash-line indicates the eye-guided reference.

b) Intrinsic temperature invariance of the RBM frequency

In this work, we observe minimal change in the RBM frequency due to temperature alone. This observation is apparent in most of the trajectories from $T > T_{\mathrm{max}}$, where the RBM is shown to reach a limiting value and remain essentially constant over a change in temperature as high as 800 K. We also observe that approximately 1/3 of CNTs synthesized in this suspended format show no RBM trajectory with temperature and these also indicate an invariance of the RBM to temperature. For this, we grew CNTs on commercially available porous Si_3N_4 membranes (Ted Pella, Inc) via the same CVD method mentioned above. In an investigation of 9 CNTs on a TEM grid suspended across a porous Si_3N_4 membrane (200 nm, 500 nm, and 5 μm), 3 CNTs from each porous substrate shows RBMs unaffected by the temperature for each scan temperature variation under vacuum ($\sim 10^{-5}$ bar) (**Supplementary Fig. 1-2**). This observation suggests that any intrinsic RBM shift with temperature is negligible, and hence has not been considered in the modeling conducted in the main text.

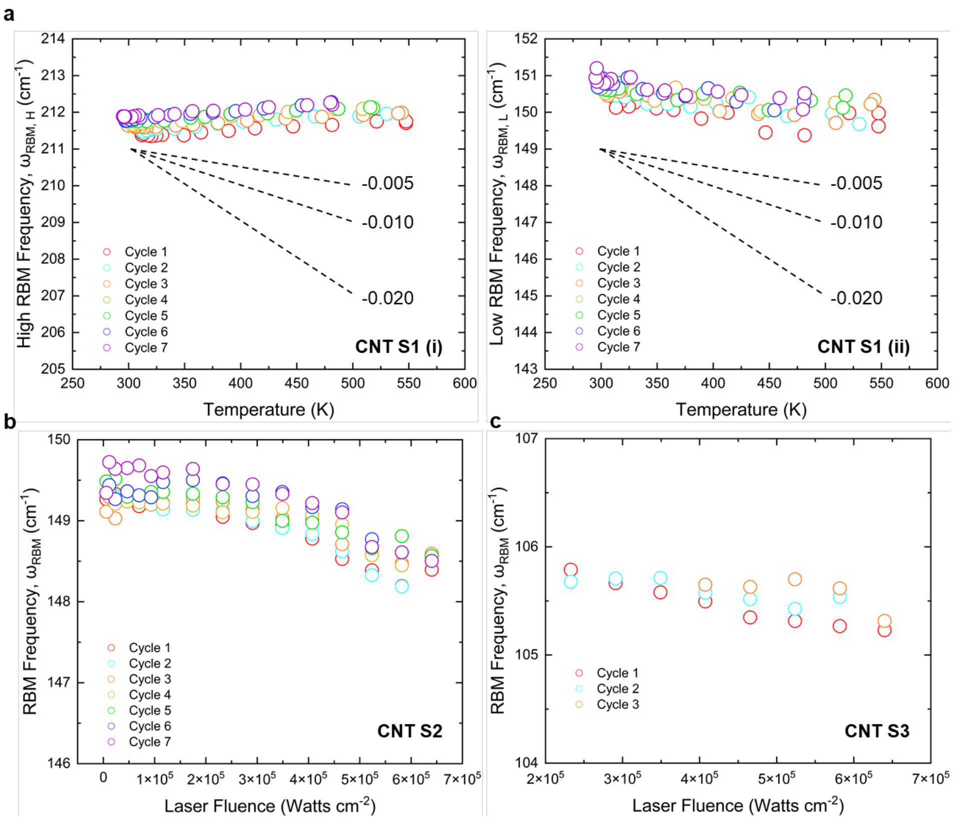

Supplementary Fig. 1-2| No distinct RBM frequency shifts of three CNTs upon laser heating. **a**, CNT S1 on a 200 nm pore membrane exhibits two RBMs (**i**) high frequency $\omega_{\mathrm{RBM,H}}$ (**ii**) low frequency $\omega_{RBM,L}$ using 633 nm laser and both RBMs demonstrate no appreciable RBM shift within the resolution of our spectrometer (0.2 cm^{-1}) upon laser heating to 550 K with a pressure of 1.2×10^{-5} bar. The black lines represent different $\frac{d\omega}{dT}$ values (-0.005, -0.010, and -0.020 in units of K^{-1}). **b**, CNT S2 on a 500 nm pore membrane exhibits 1 RBM shift of approximately 1 cm^{-1} upon 633 nm laser heating to 6.4×10^{5} Watts/cm^{2} with a pressure of 7.4×10^{-6} bar. However, the CNT S2 was damaged during electron diffraction by TEM and no temperature calibration was performed. **c**, CNT S3 on a 5 µm membrane demonstrates no RBM shift within the resolution of our spectrometer (0.2 cm^{-1}) upon 633 nm laser heating to 6.4×10^{5} Watts/cm^{2}. The CNT S3 was destroyed after 2.5 laser heating cycles and no temperature calibration was conducted.

2: Summary of Full Raman Spectra for CNT G, X, and F

We include the full Raman spectra for all of the temperature scans for the three DWNTs studied in this work. The data span 93 RBM trajectories in all, all under different levels of vacuum from 10^{-3} to 10^{-8} bar. For all figures, the panels **a** (RBM region) and **b** (G band region) are the Raman spectra of the first local laser power cycle scans. The panels **c** (RBM region) and **d** (G band region) are the full temperature scans for this experiment.

a) CNT G full Raman spectra

filename = ["210628_210621Bs02x0168_633nm_p1.2e-3mbar.csv"];

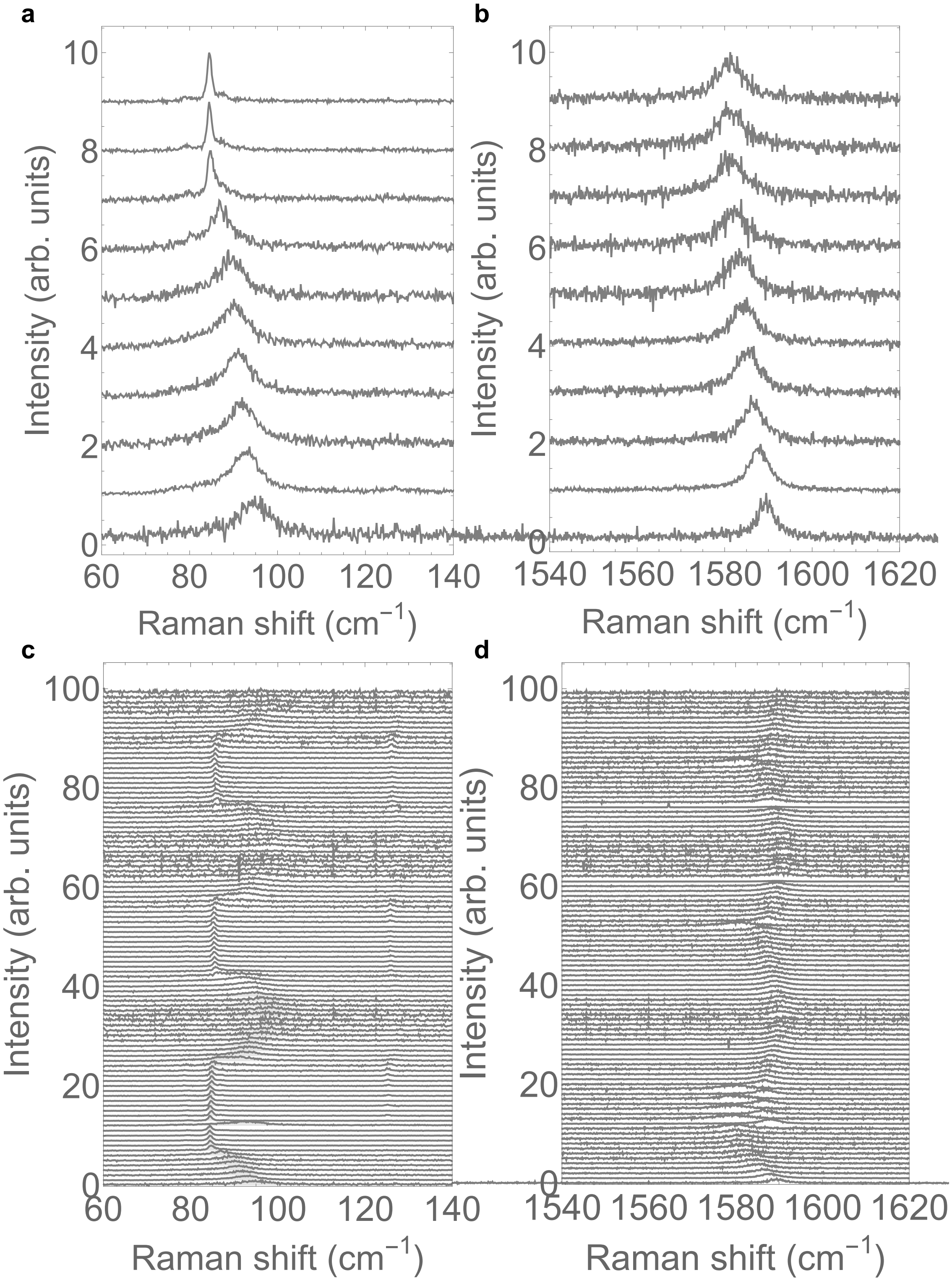

filename = ["210628_210621Bs03x0181_633nm_p1.2e-3mbar.csv"];

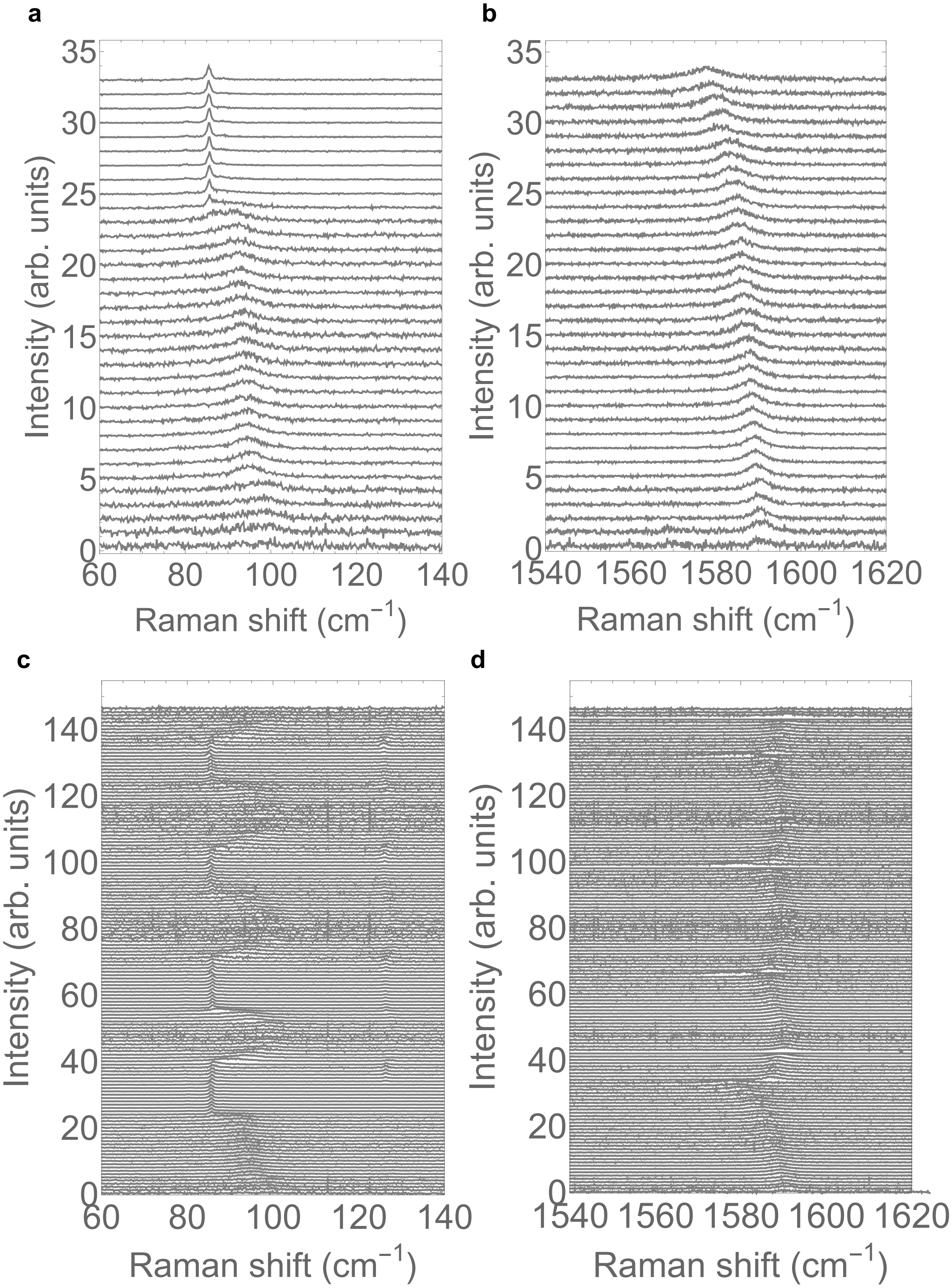

filename = ["210628_210621Bs04x0188_633nm_p1.2e-3mbar.csv"];

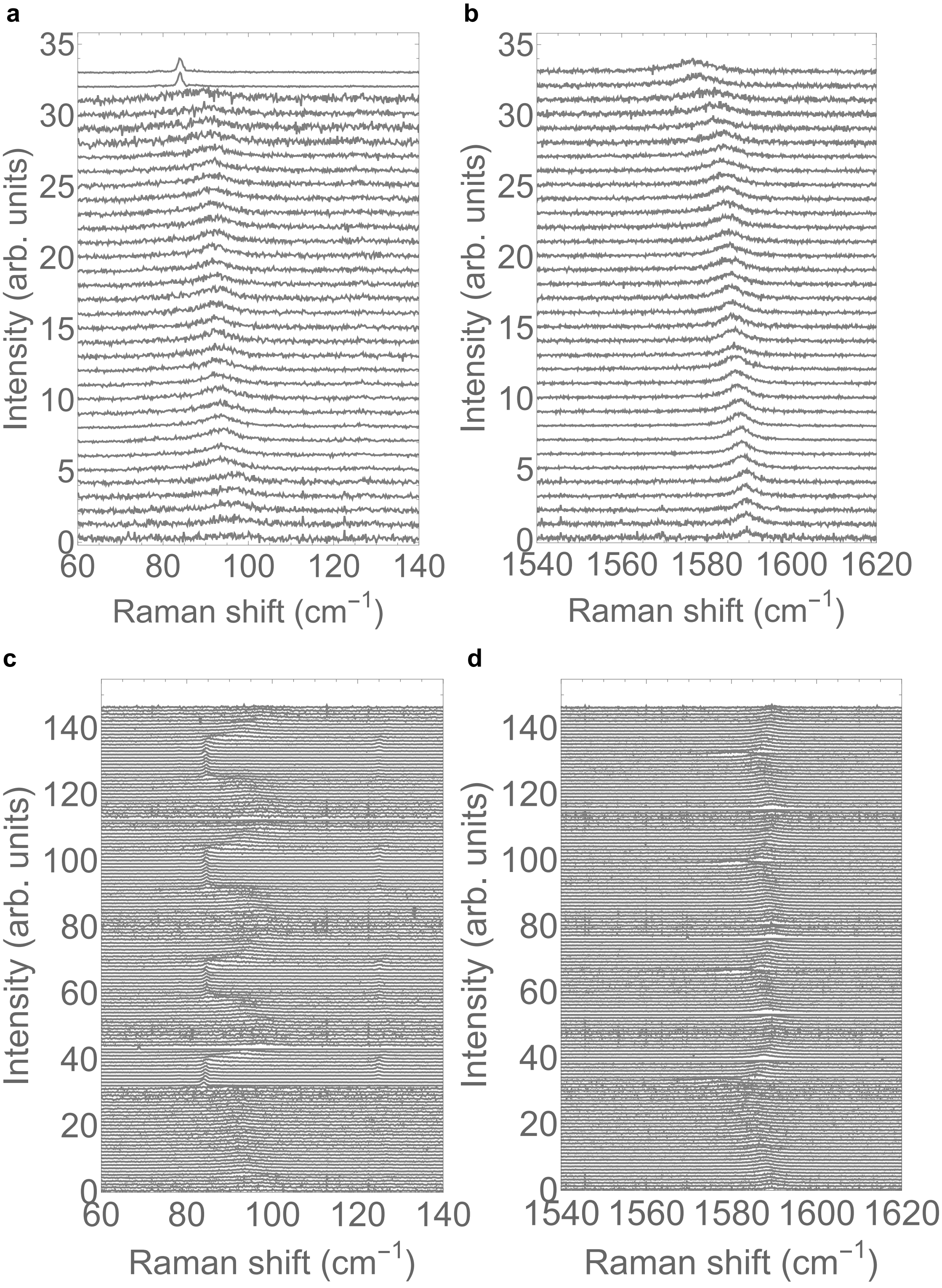

filename = ["210628_210621Bs05x0196_633nm_p1.2e-3mbar.csv"];

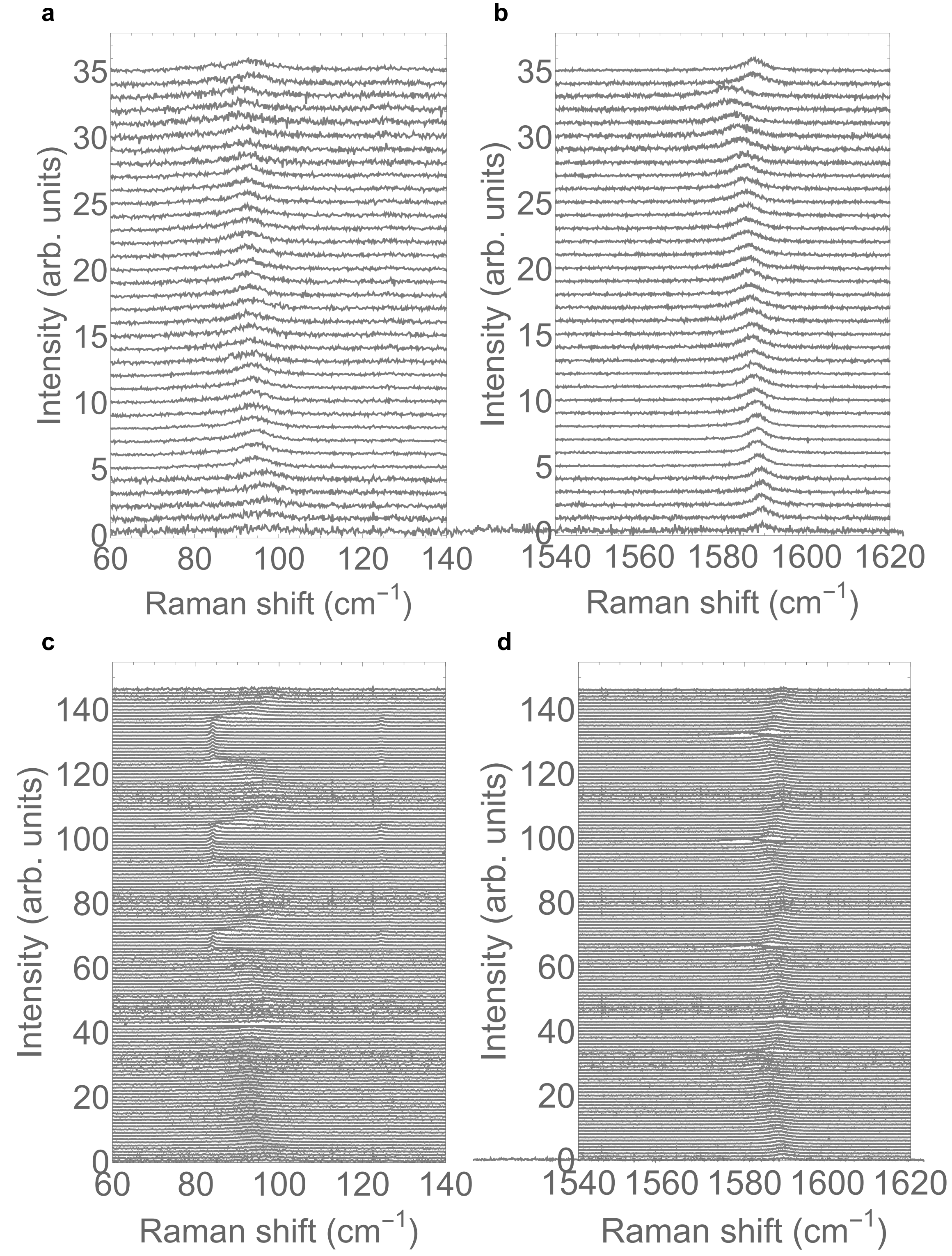

b) CNT X full Raman spectra

filename = ["Matthias_CNT-X_211113_210803Bs01x1177_02_2e-2mbar.csv"];

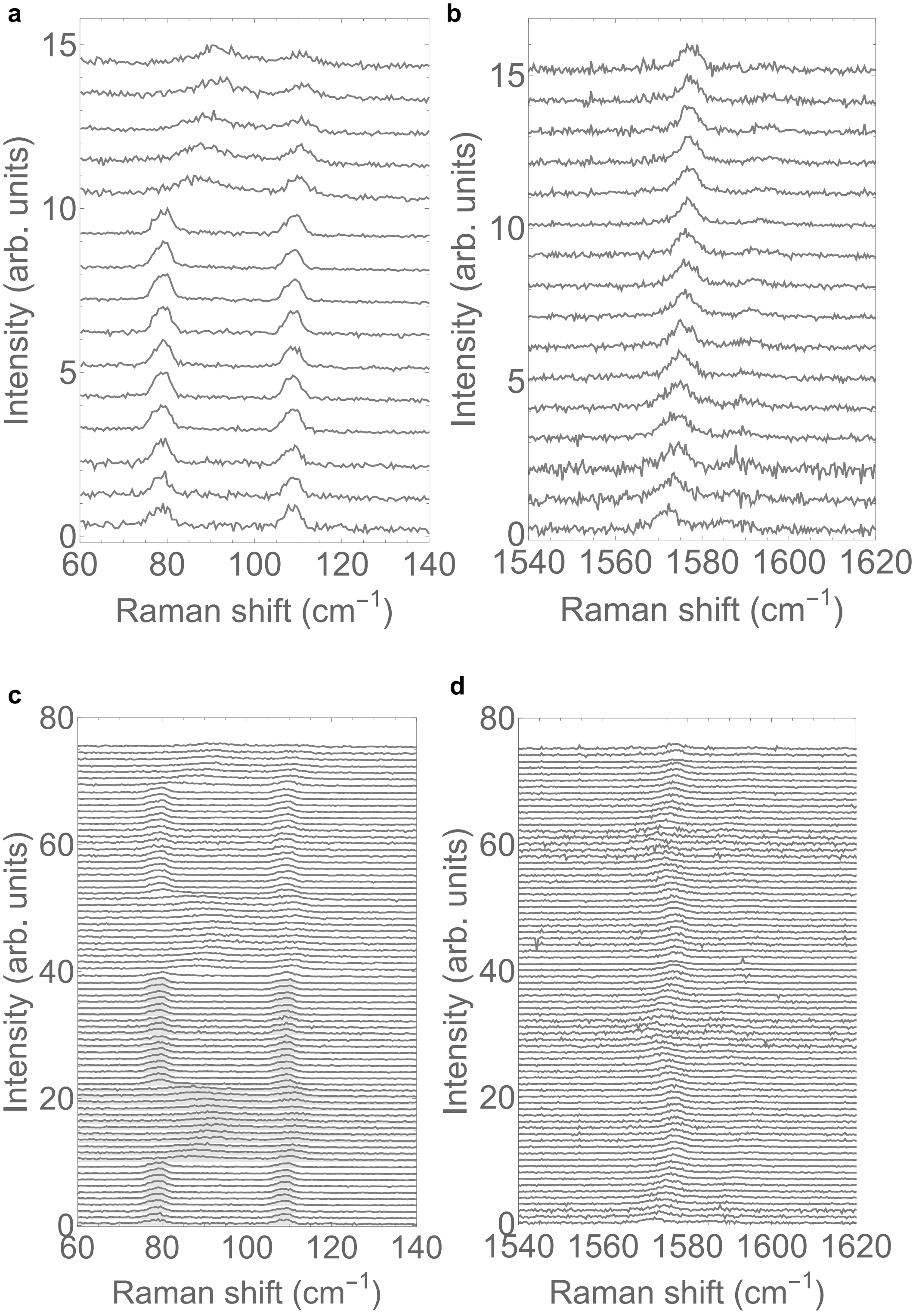

filename = ["Matthias_CNT-X_211116_210803Bs01x1177_03_2e-2mbar_afterbakeout.csv"];

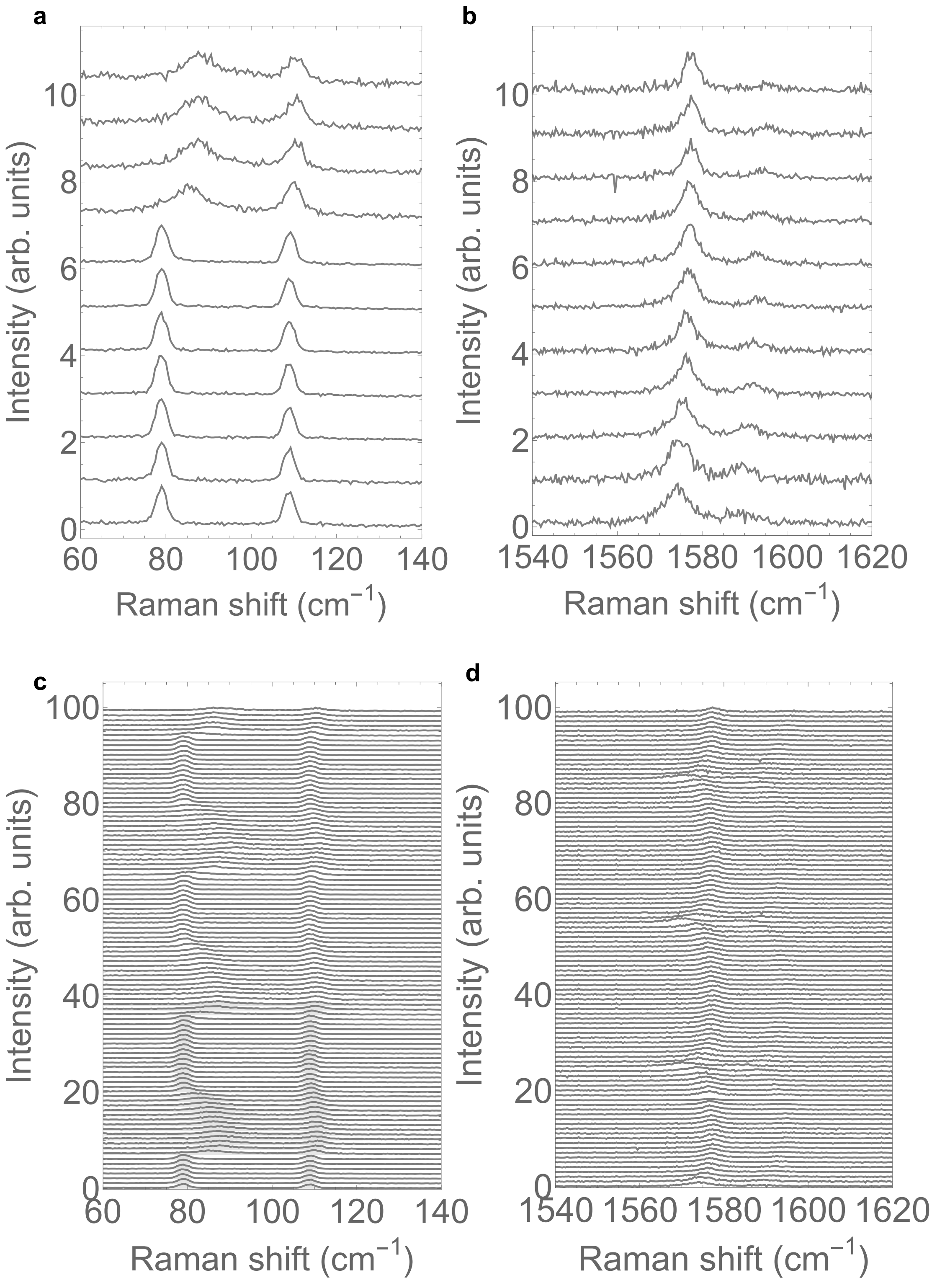

filename = ["Matthias_CNT-X_211220_210803Bs01x1177_08_3.4mbar.csv"];

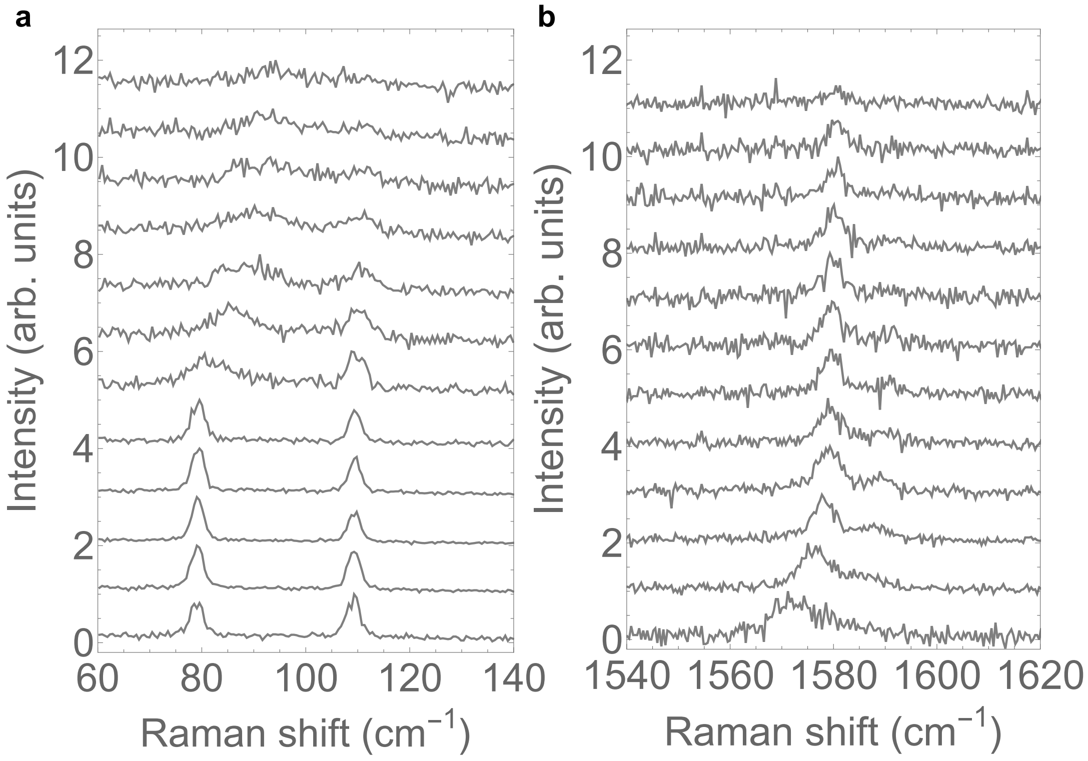

filename = ["Matthias_CNT-X_211221_210803Bs01x1177_09_3.4mbar.csv"];

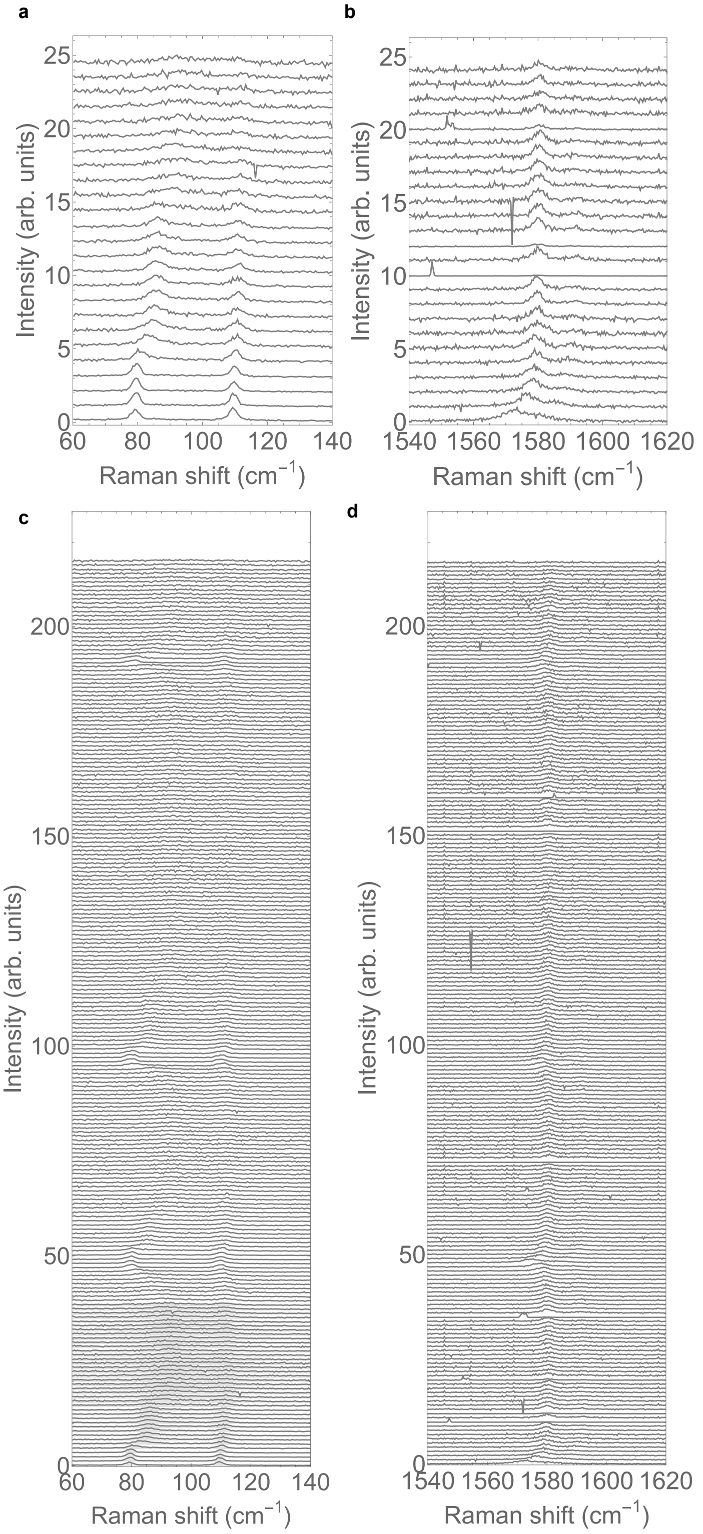

filename = ["Matthias_CNT-X_211222_210803Bs01x1177_10_2.5e-5mbar.csv"];

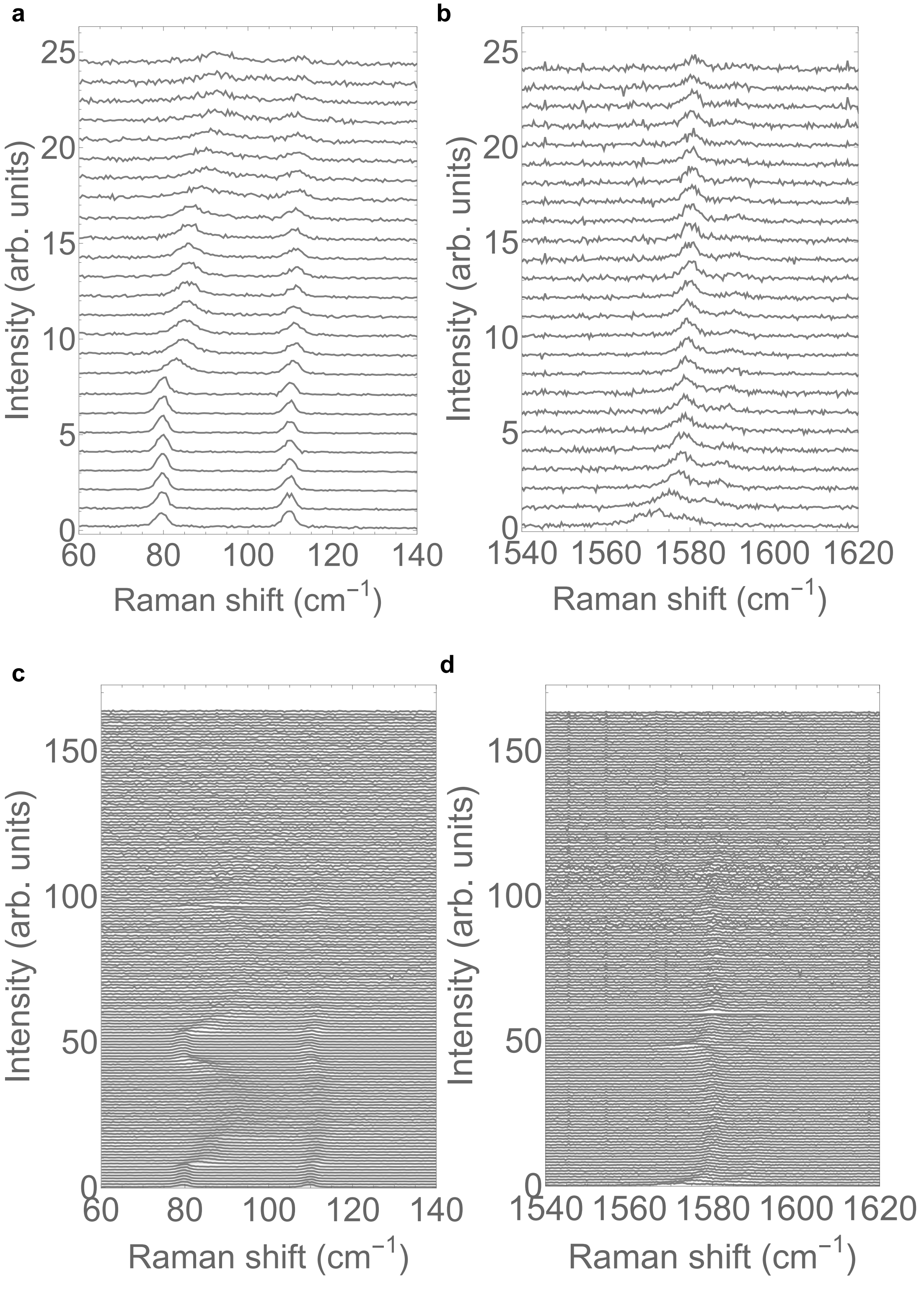

filename = ["Matthias_CNT-X_211223_210803Bs01x1177_11_2.5e-5mbar.csv"];

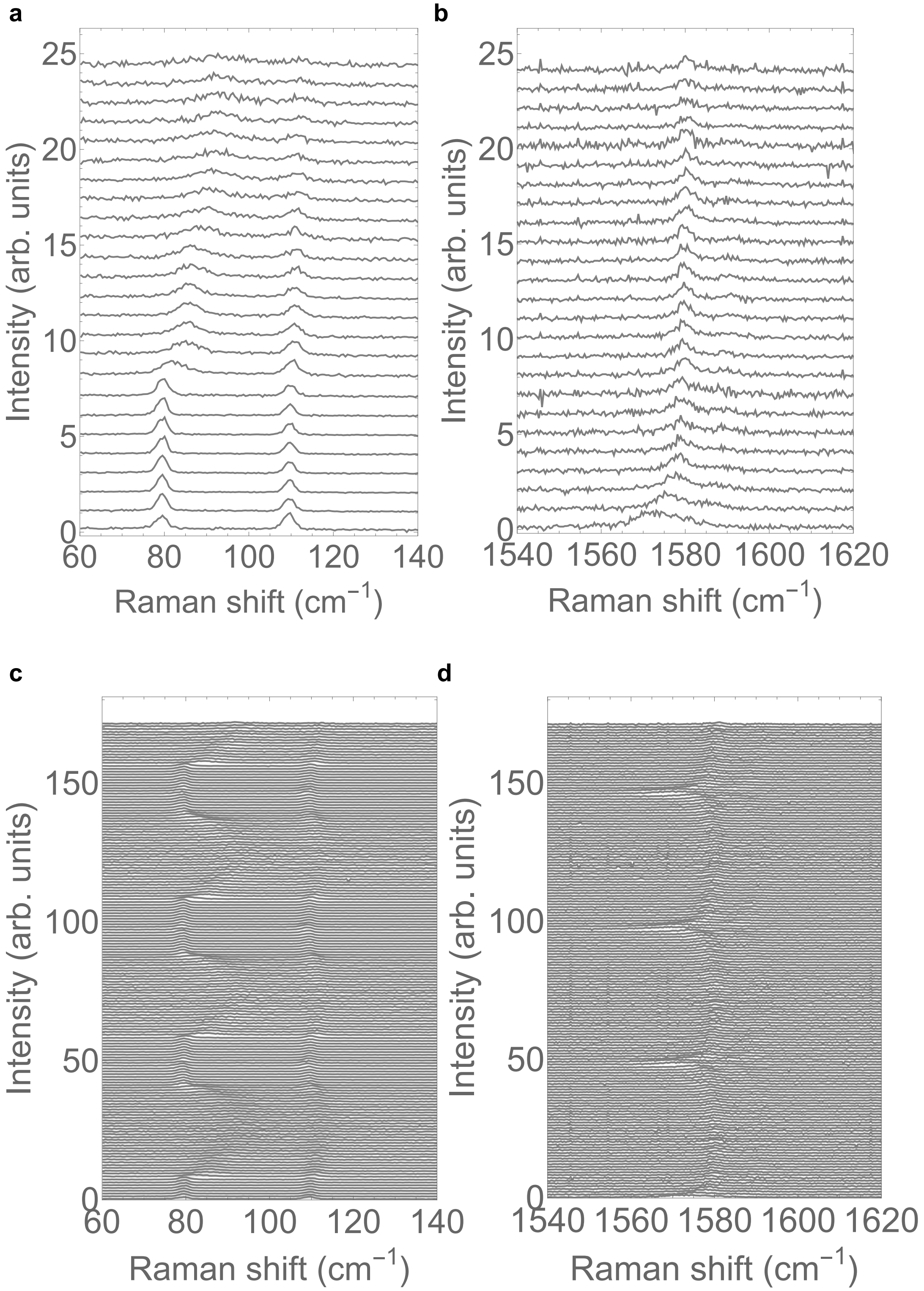

filename = ["Matthias_CNT-X_211228_210803Bs01x1177_12_1.9e-5mbar.csv"];

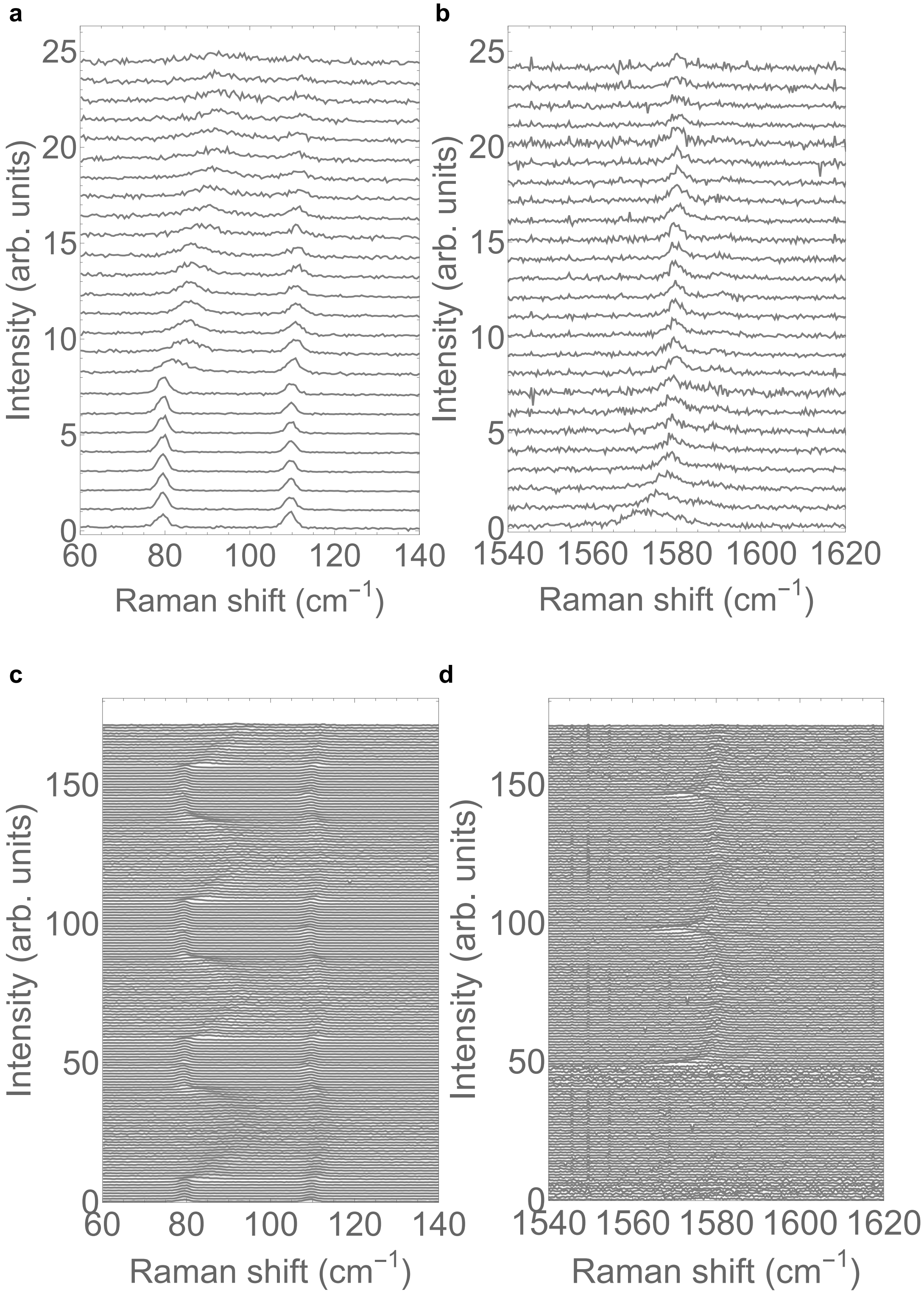

filename = ["Matthias_CNT-X_220105_210803Bs01x1177_13_1.6e-5mbar.csv"];

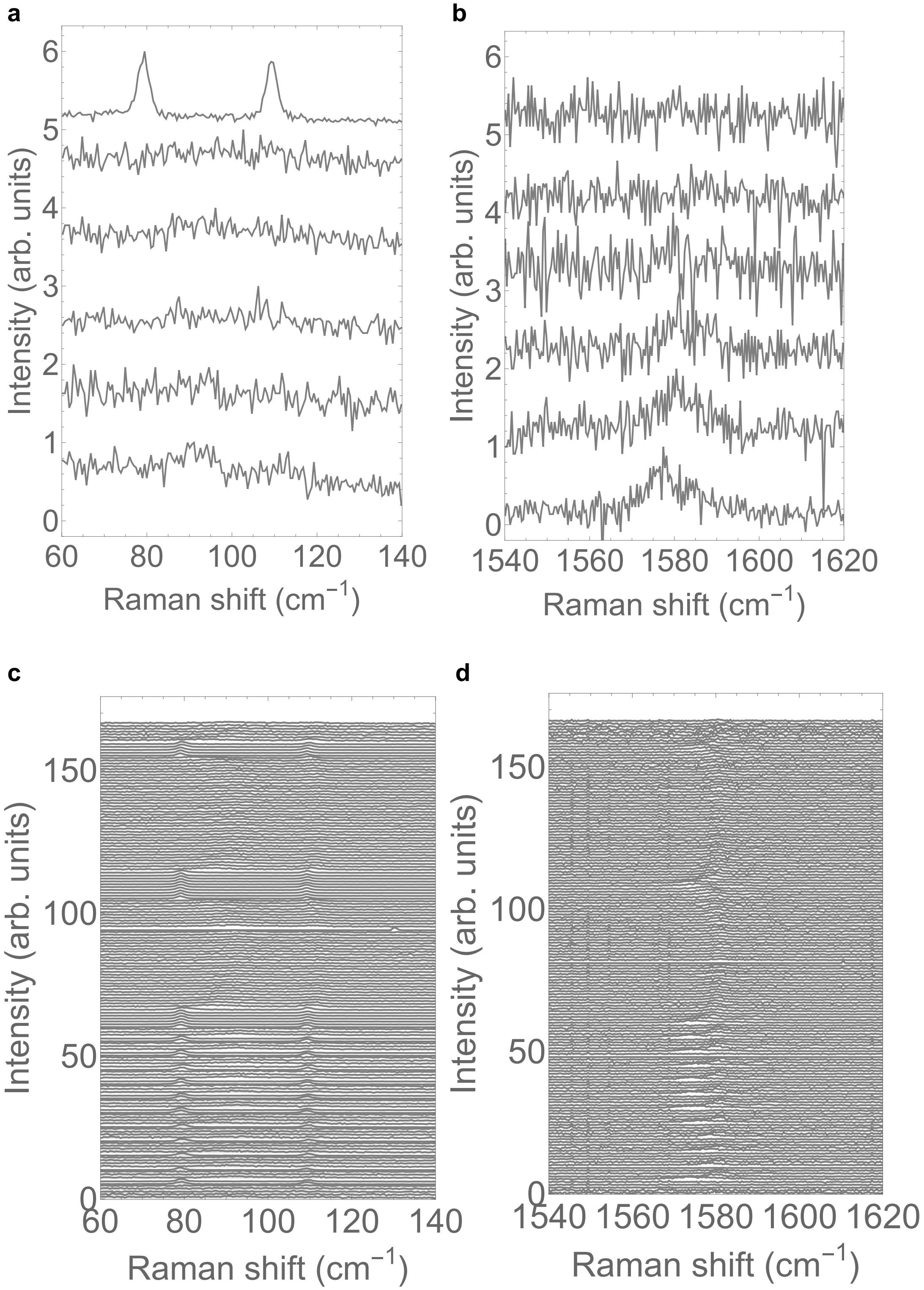

c) **CNT F Full Raman spectra**

filename = ["210519_210518As01x1132_LaserPowerScan_633nm_p7.4e-4mbar.csv"];

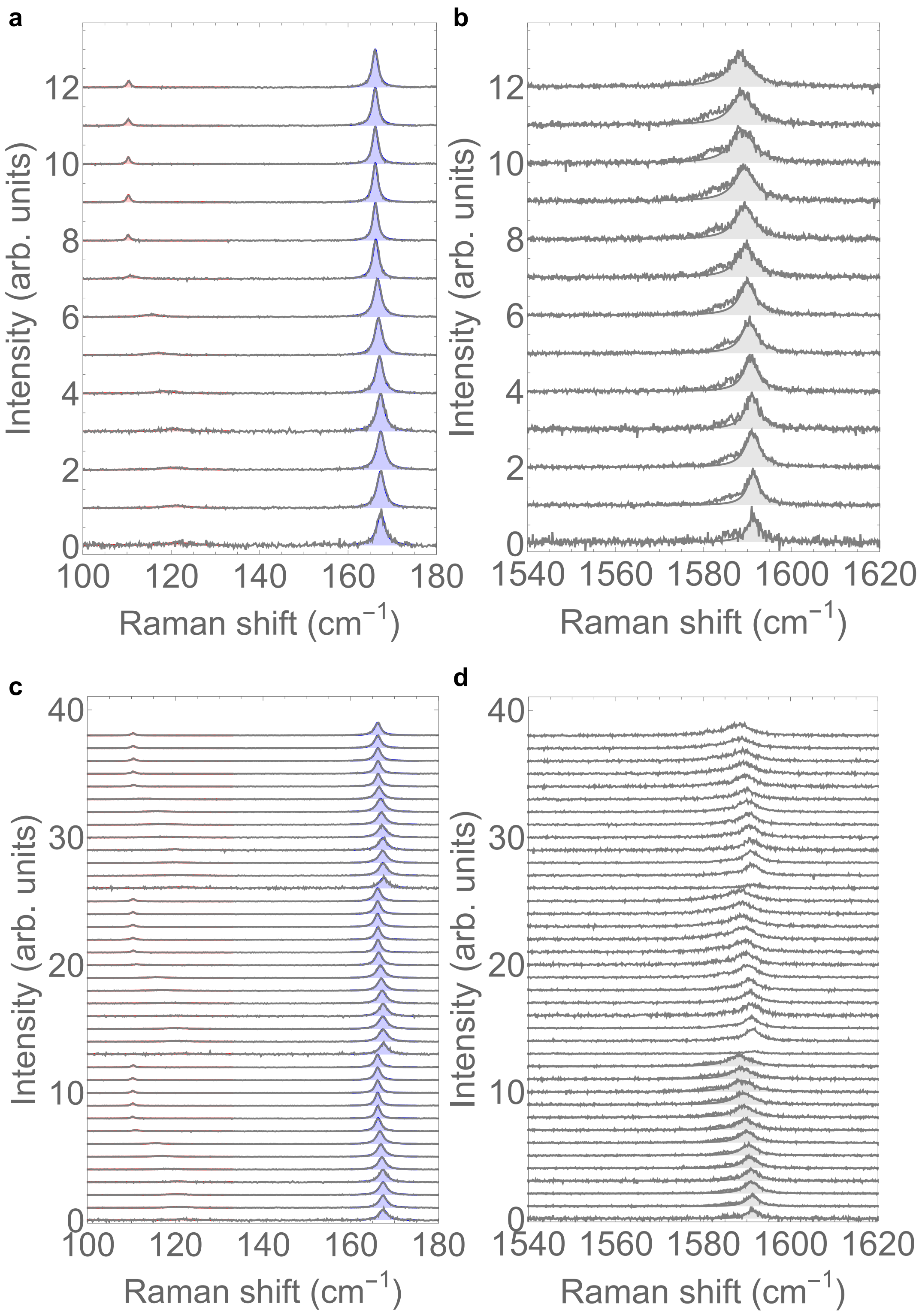

filename = ["210519_210518As02x1122_LaserPowerScan_633nm_p7.4e-4mbar.csv"];

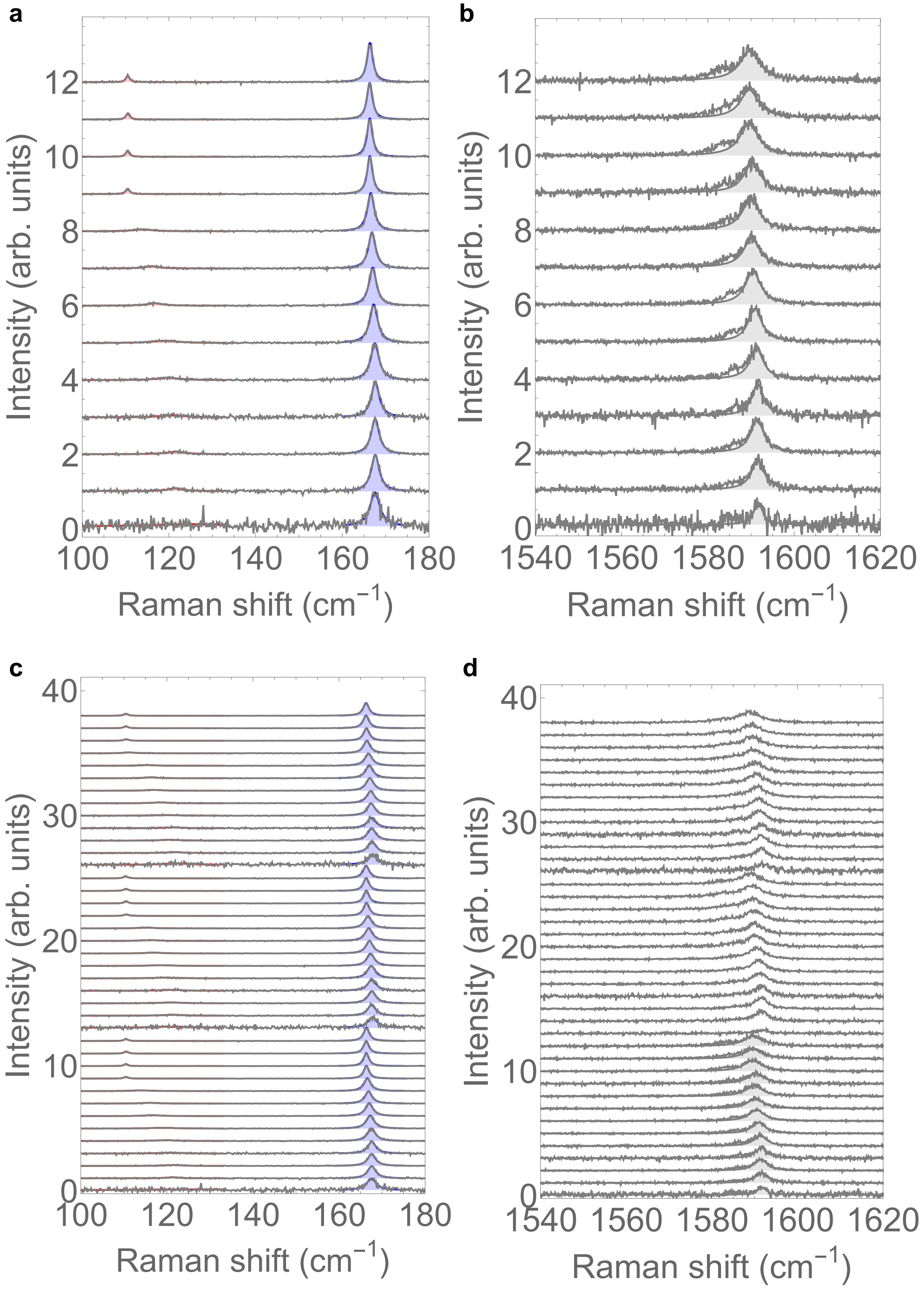

filename = ["210519_210518As03x1117_LaserPowerScan_633nm_p7.4e-4mbar.csv"];

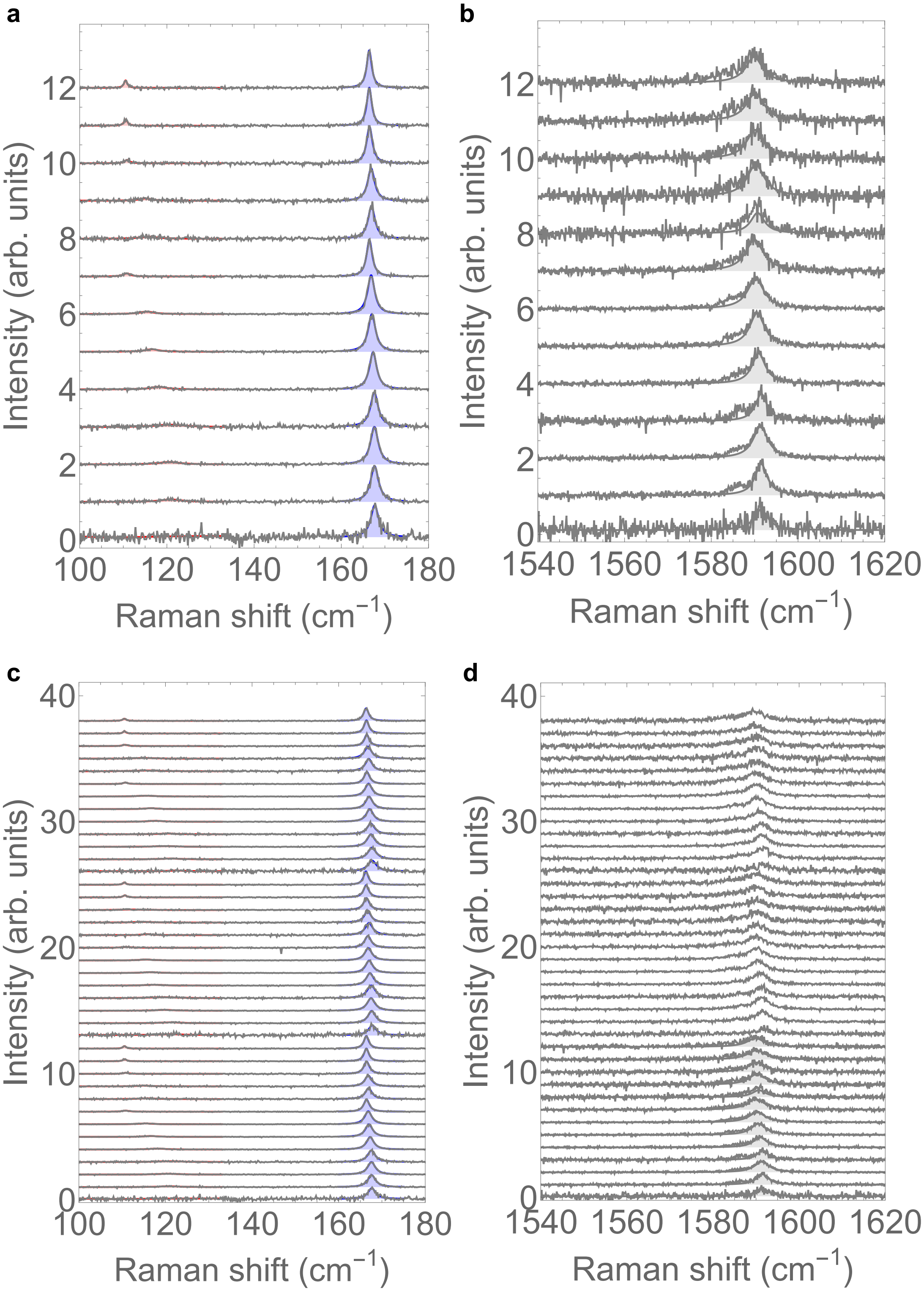

filename = ["210519_210518As04x1107_LaserPowerScan_633nm_p7.4e-4mbar.csv"];

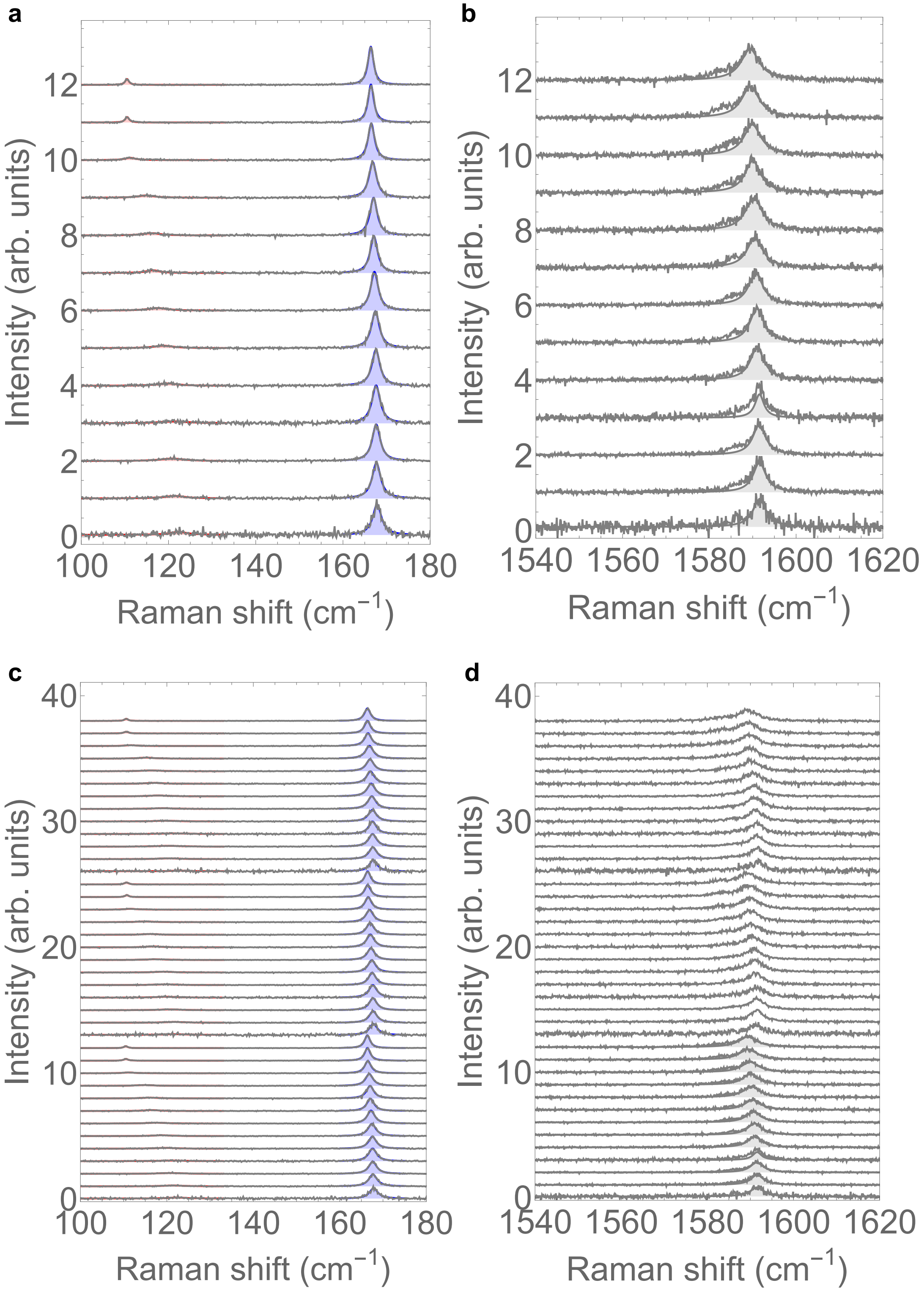

```
filename = ["210521_210518As01x1132_LaserPowerScan_633nm_p2.5e0mbar.csv"];
```

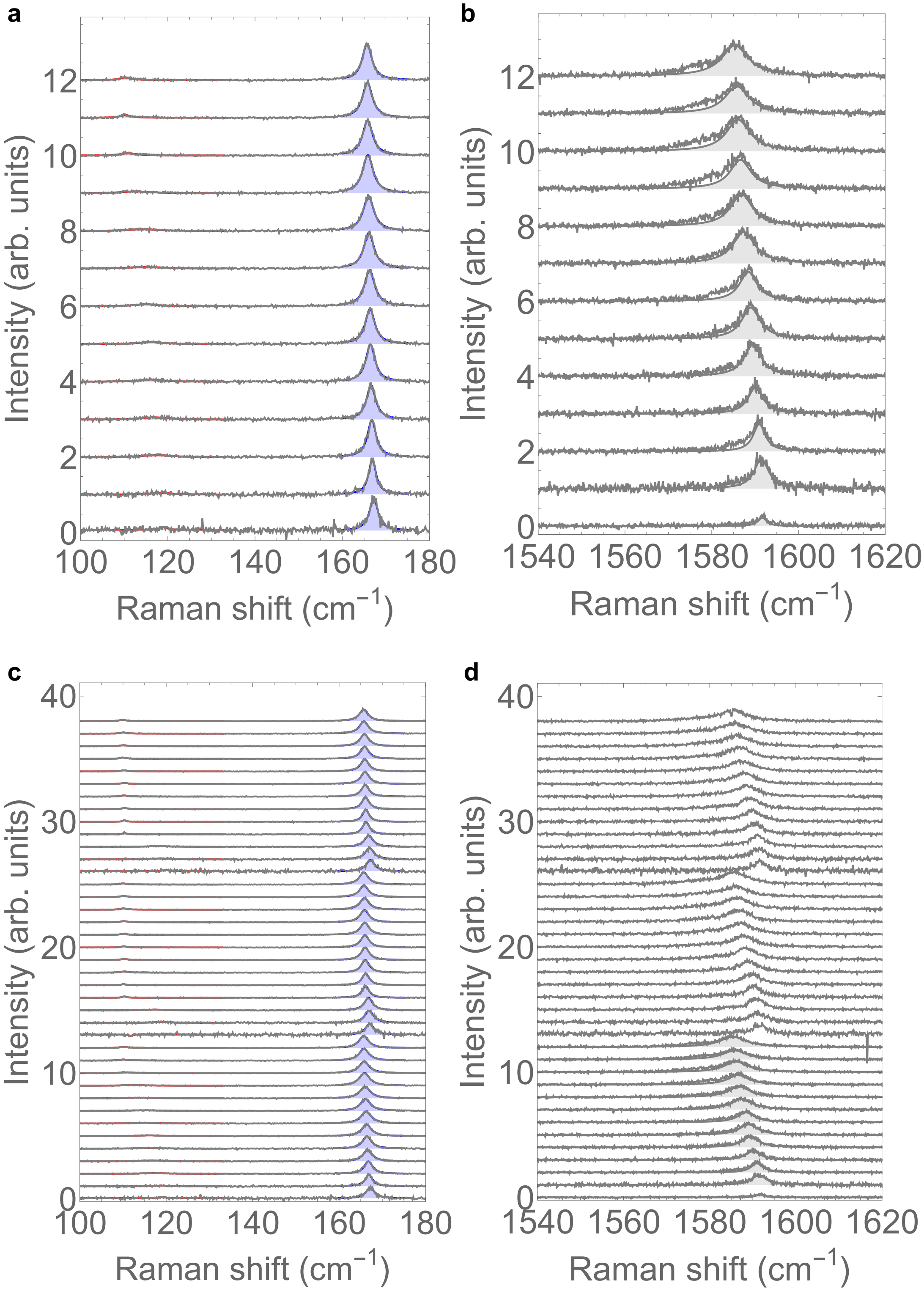

filename = ["210521_210518As02x1122_LaserPowerScan_633nm_p2.5e0mbar.csv"];

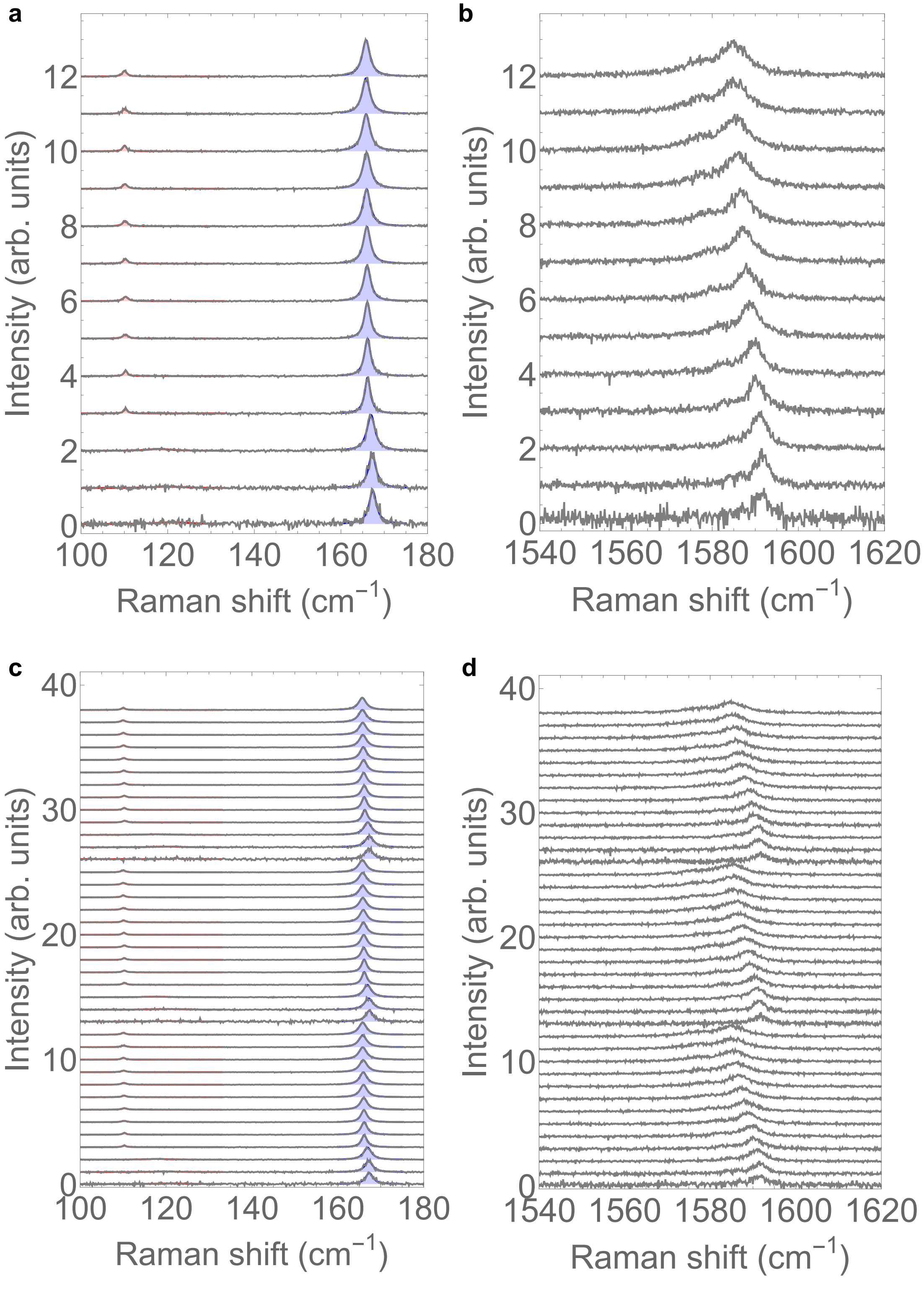

filename = ["210521_210518As03x1117_LaserPowerScan_633nm_p2.5e0mbar.csv"];

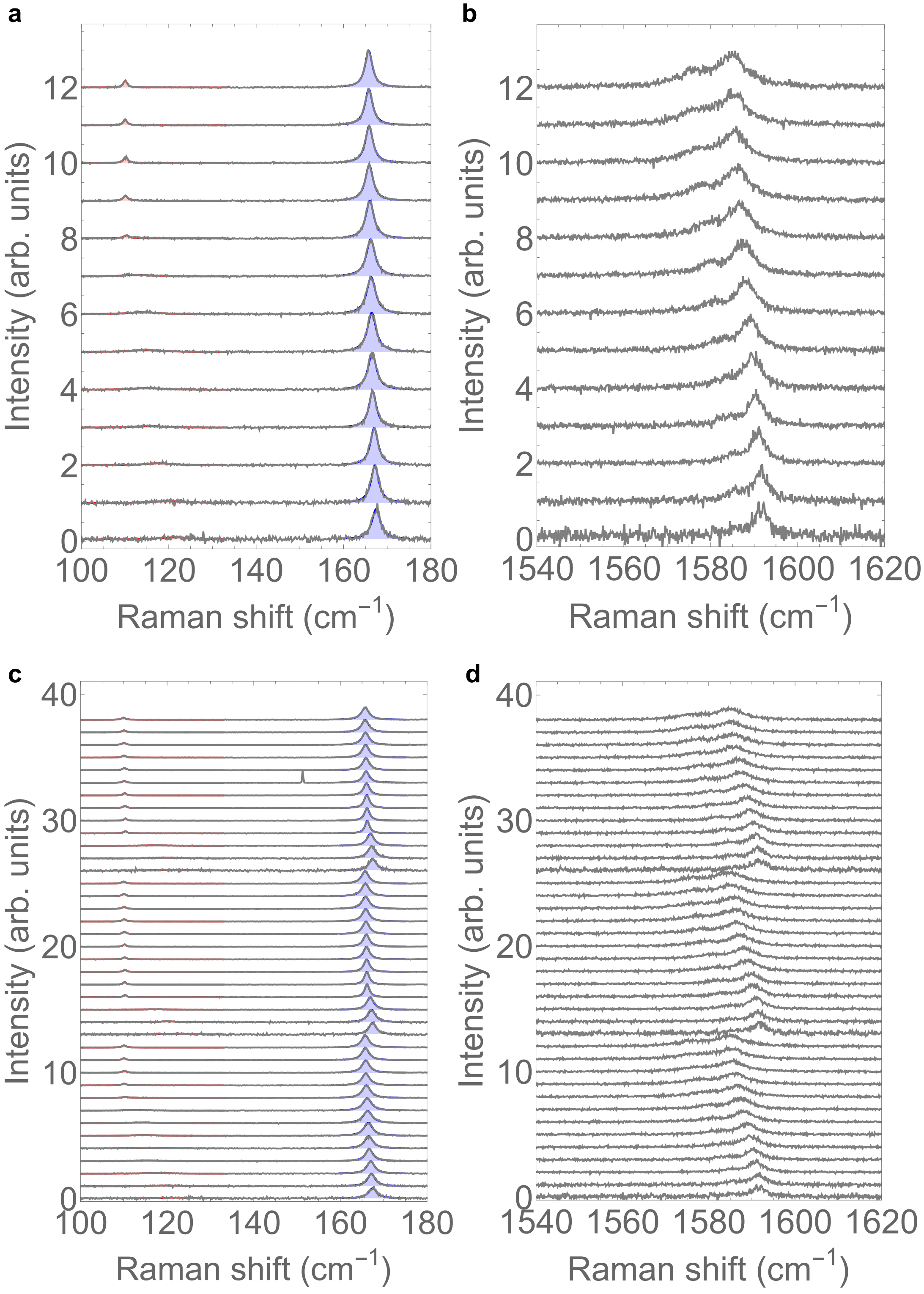

filename = ["210521_210518As04x1107_LaserPowerScan_633nm_p2.5e0mbar.csv"];

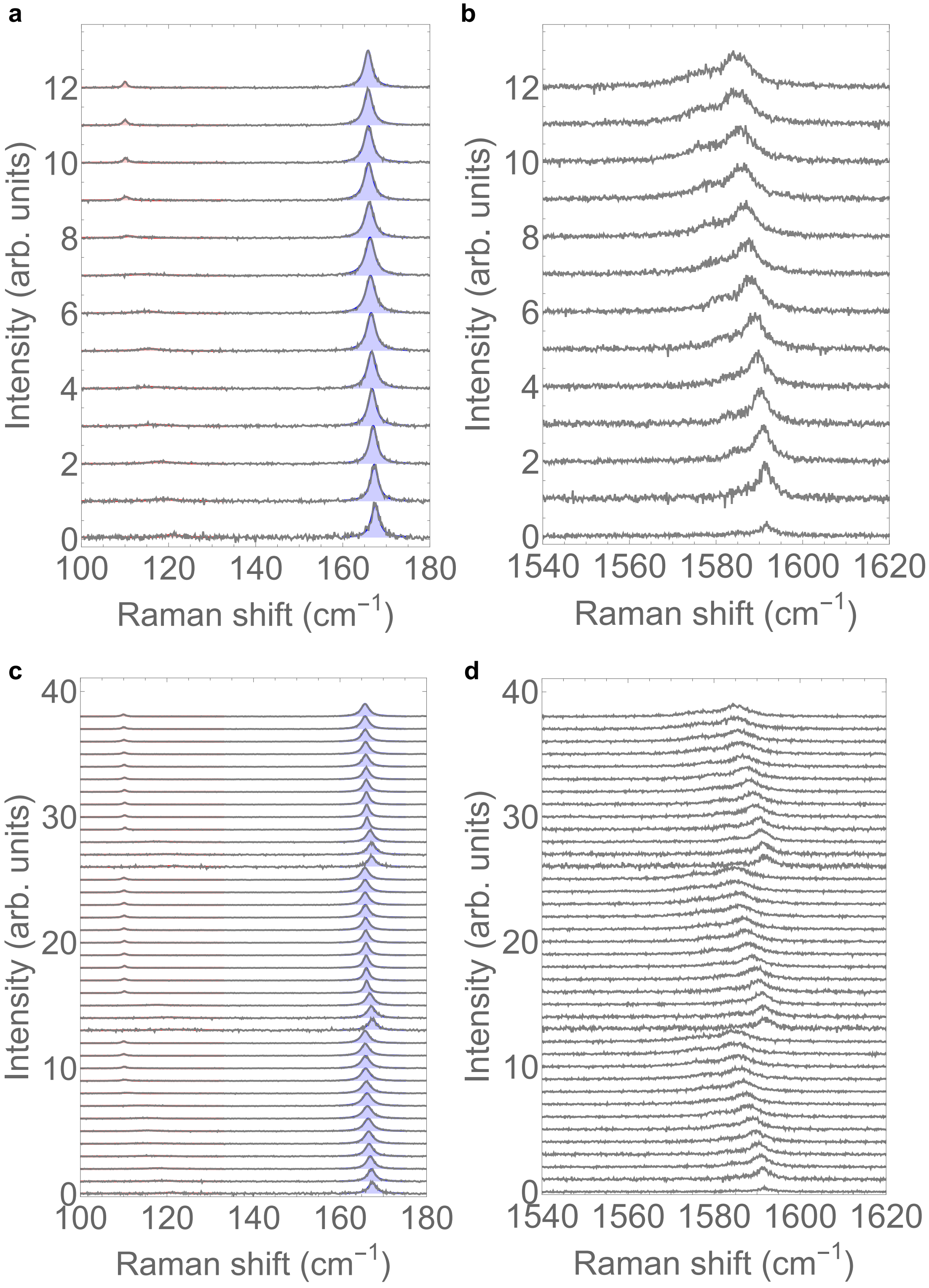

filename = ["210525_210518As02x1122_LaserPowerScan_633nm_p1.2e-3mbar.csv"];

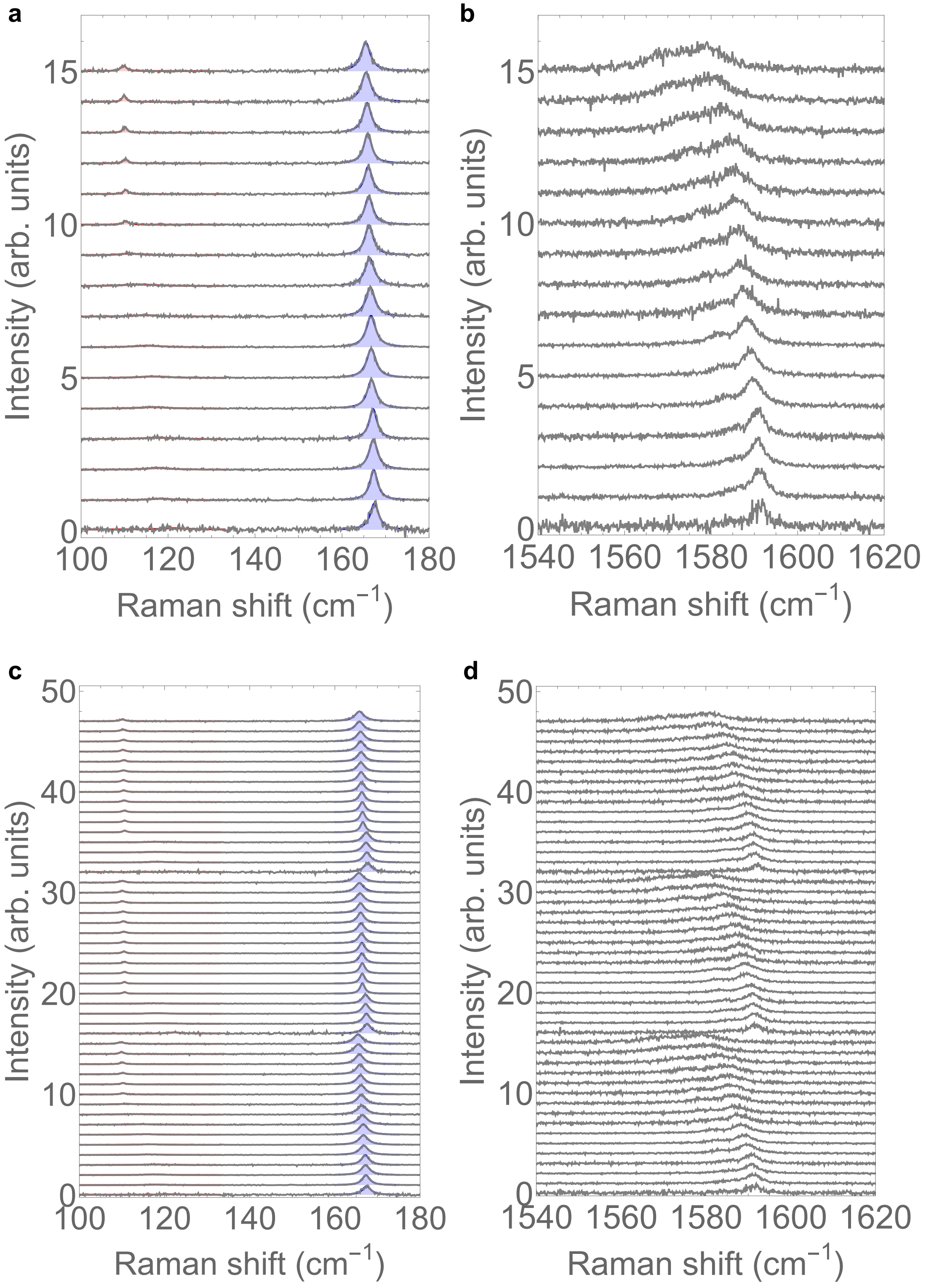

filename = ["210525_210518As03x1117_LaserPowerScan_633nm_p1.2e-3mbar.csv"];

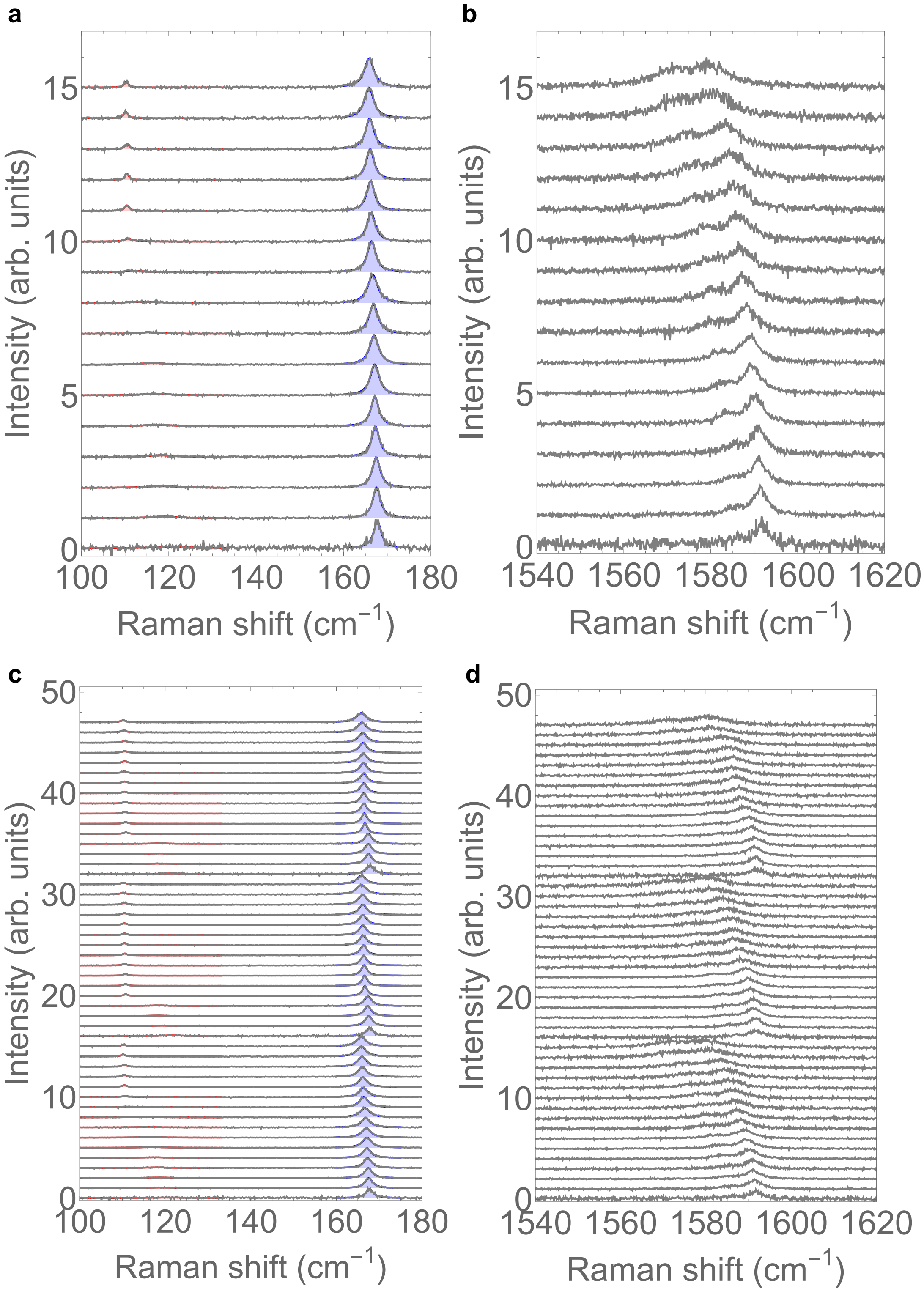

filename = ["210525_210518As04x1107_LaserPowerScan_633nm_p1.2e-3mbar.csv"];

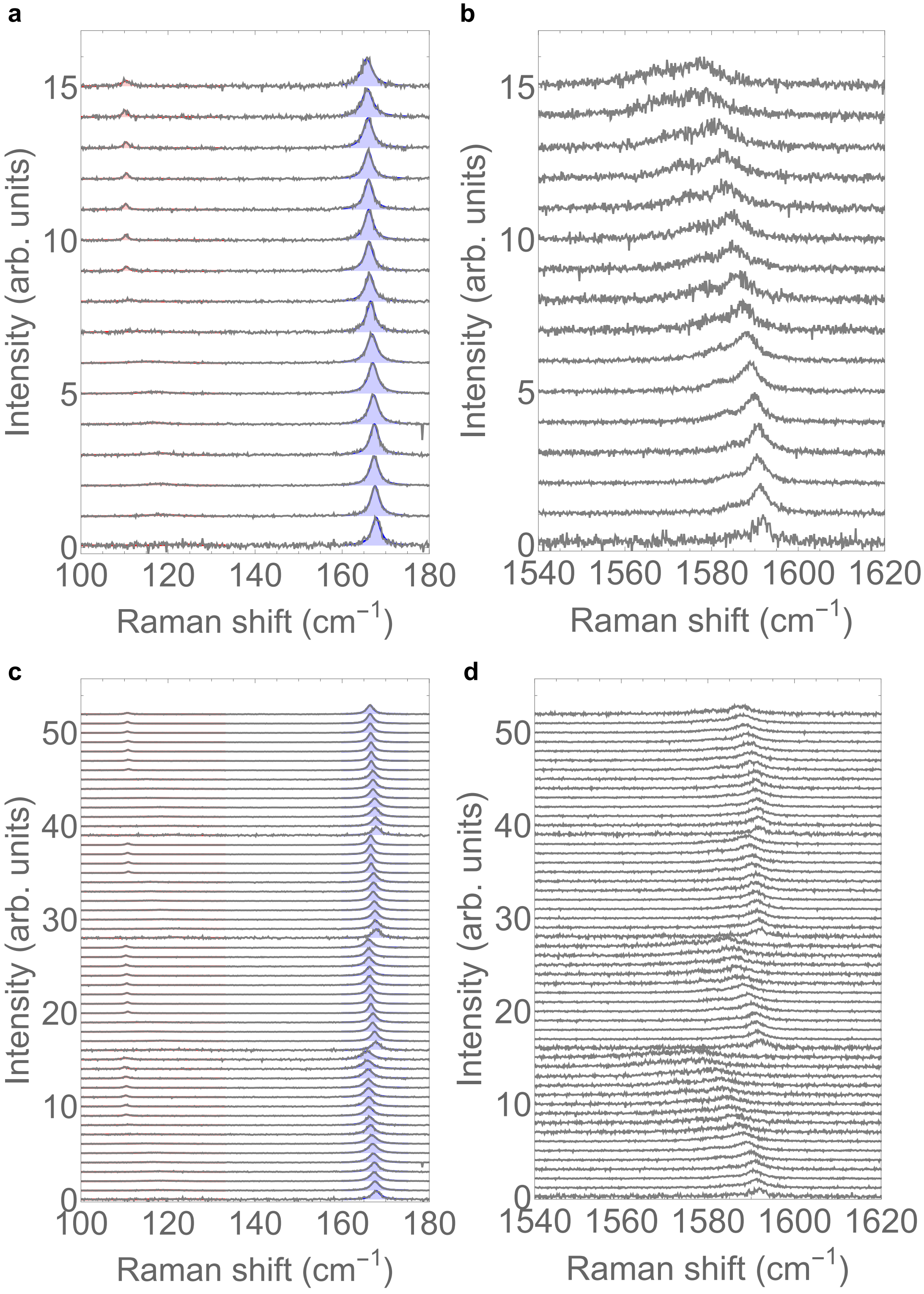

3: Summary of All RBM Trajectories and Derivative Analysis for CNT G, X, and F

We include for reference all of the temperature scans, model fitting and derivative analysis for the three DWNTs studied in this work. The data span 93 RBM trajectories in all, all under different levels of vacuum from 10^{-3} to 10^{-8} bar.

a) CNT G RBM Thermal Trajectories and Derivative Analysis

filename = ["210628_210621Bs02x0168_633nm_p1.2e-3mbar.csv"];

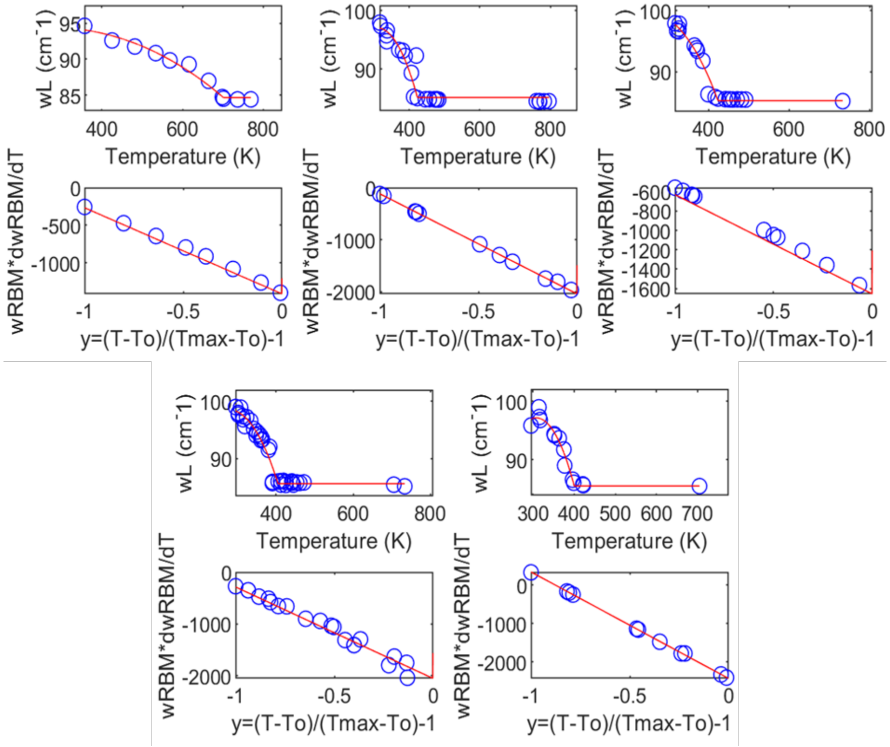

filename = ["210628_210621Bs03x0181_633nm_p1.2e-3mbar.csv"];

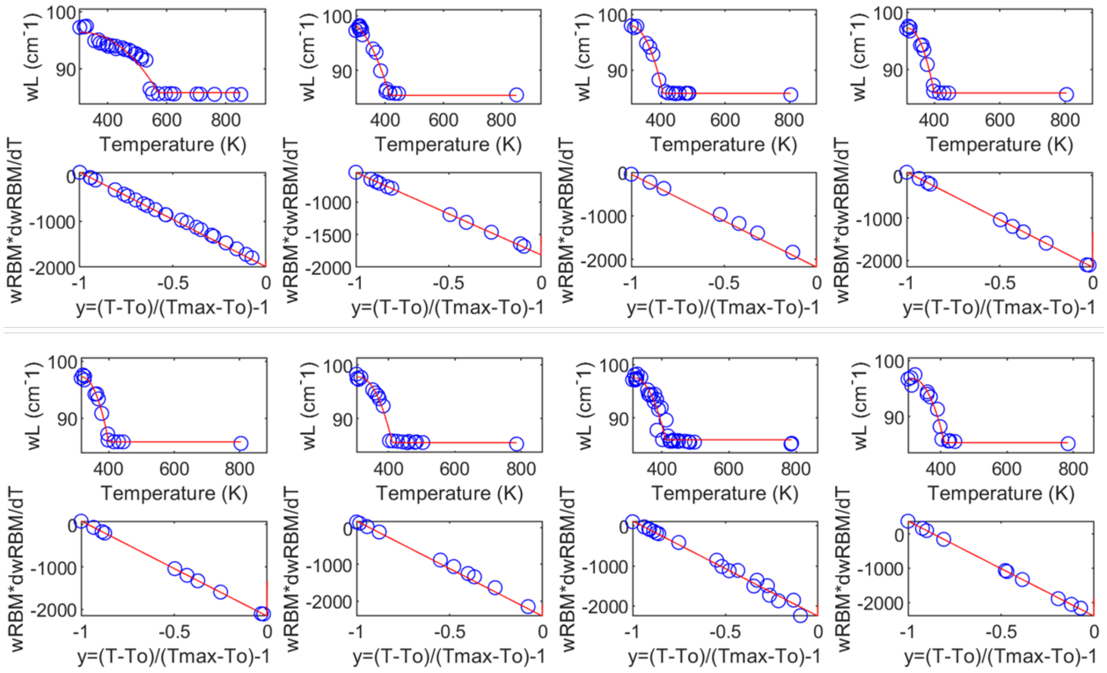


```
filename = ["210628_210621Bs04x0188_633nm_p1.2e-3mbar.csv"];
```

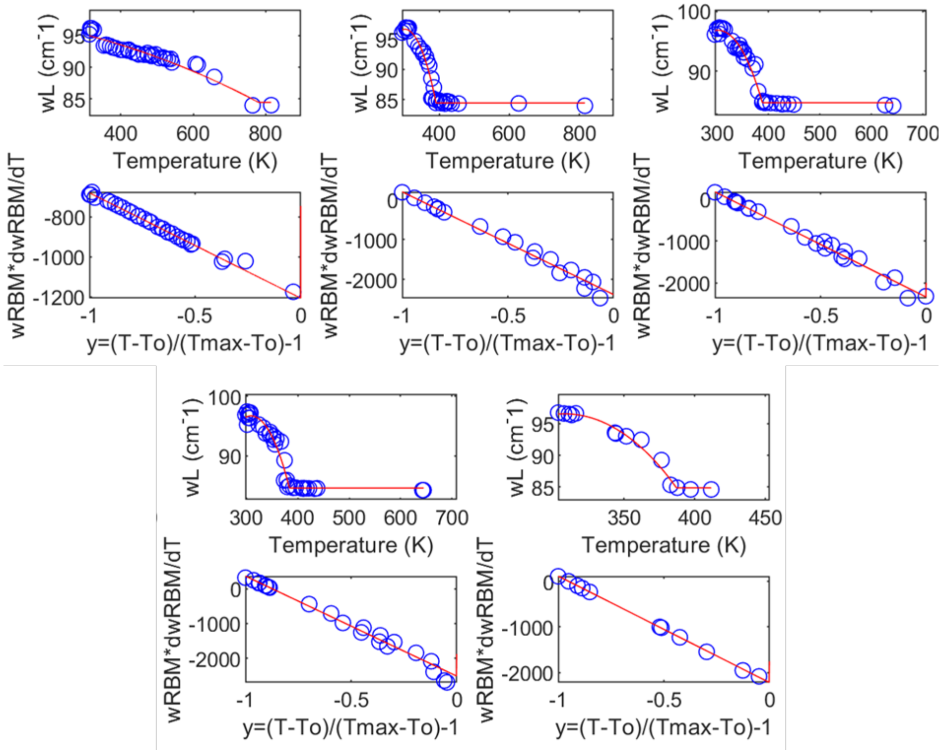


```
filename = ["210628_210621Bs05x0196_633nm_p1.2e-3mbar.csv"];
```

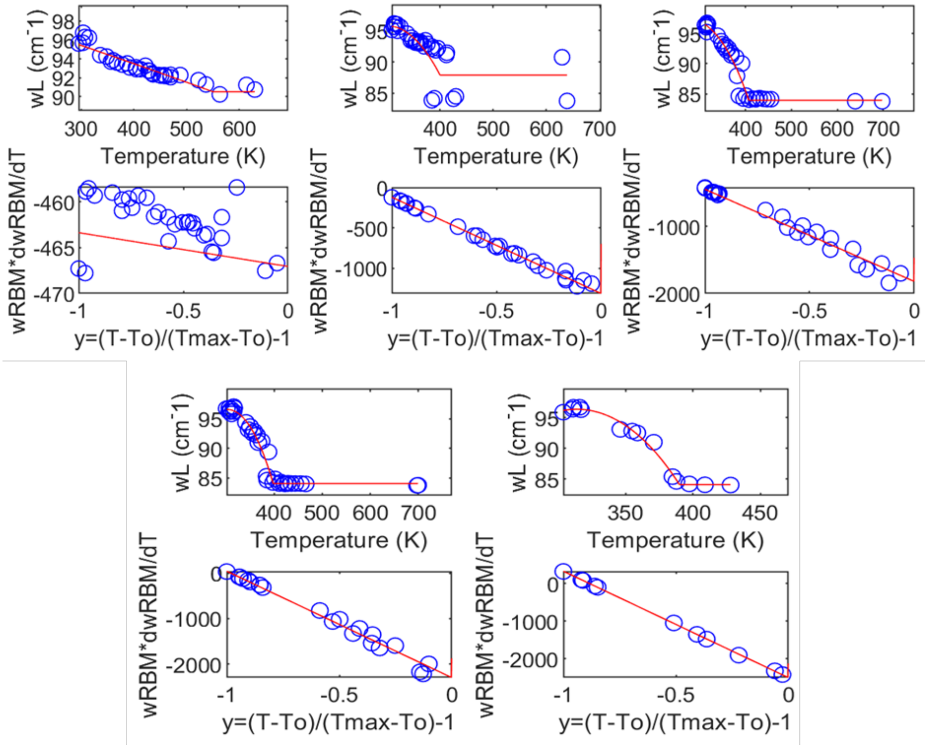

b) CNT X RBM Thermal Trajectories and Derivative Analysis

filename = ["Matthias_CNT-X_211113_210803Bs01x1177_02_2e-2mbar.csv"];

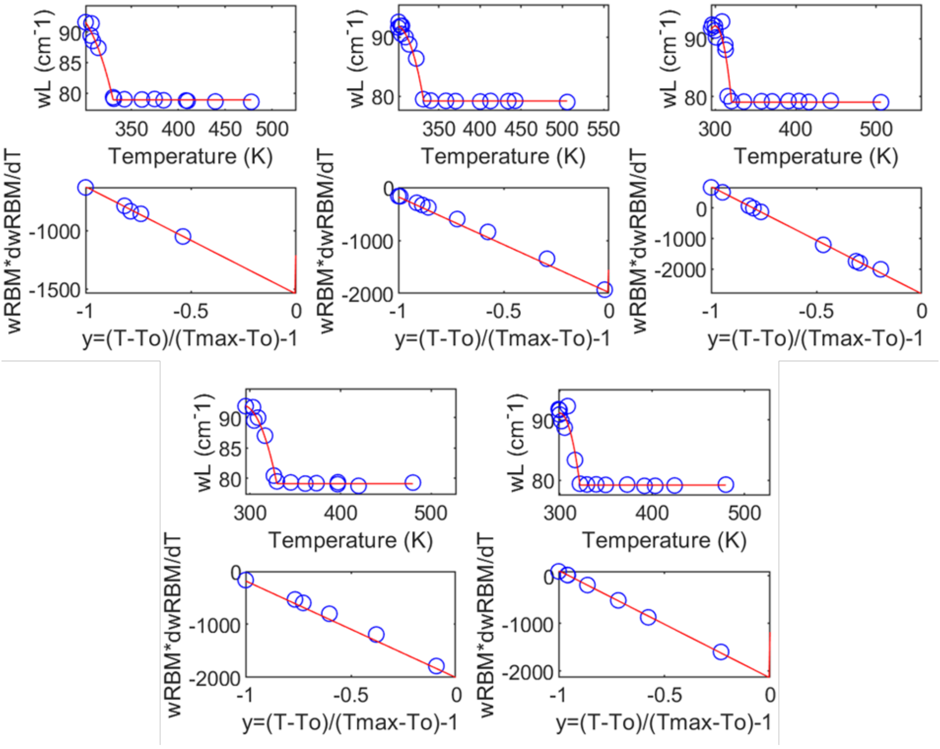

filename = ["Matthias_CNT-X_211116_210803Bs01x1177_03_2e-2mbar_afterbakeout.csv"];

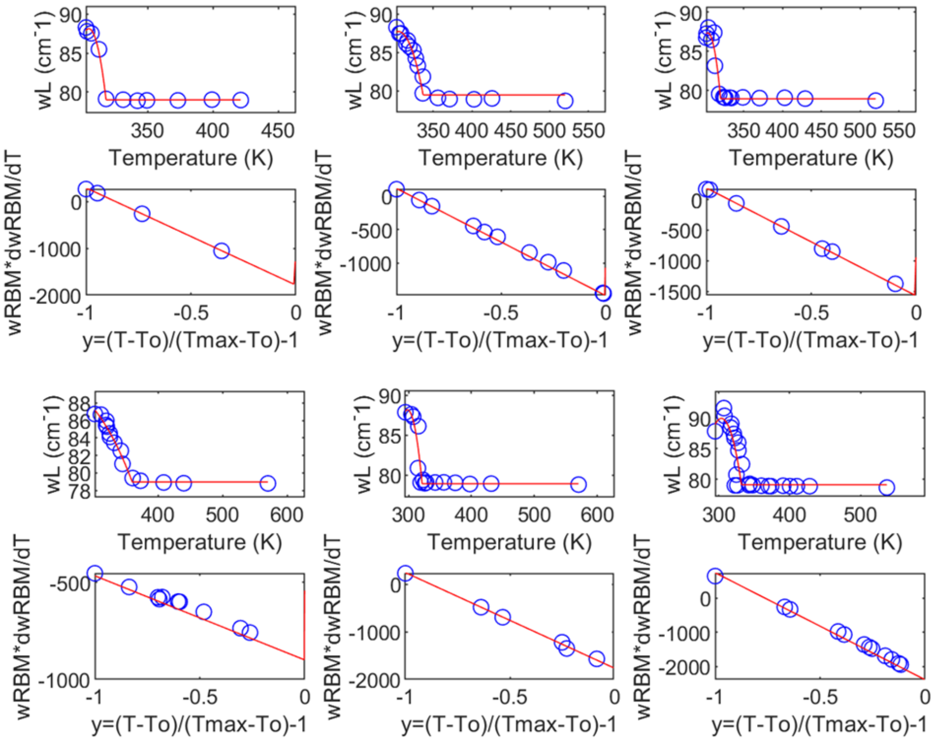

filename = ["Matthias_CNT-X_211220_210803Bs01x1177_08_3.4mbar.csv"];

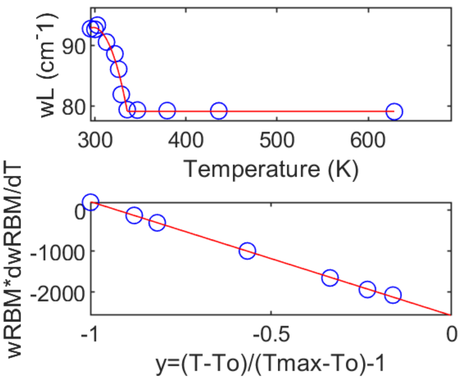

filename = ["Matthias_CNT-X_211221_210803Bs01x1177_09_3.4mbar.csv"];

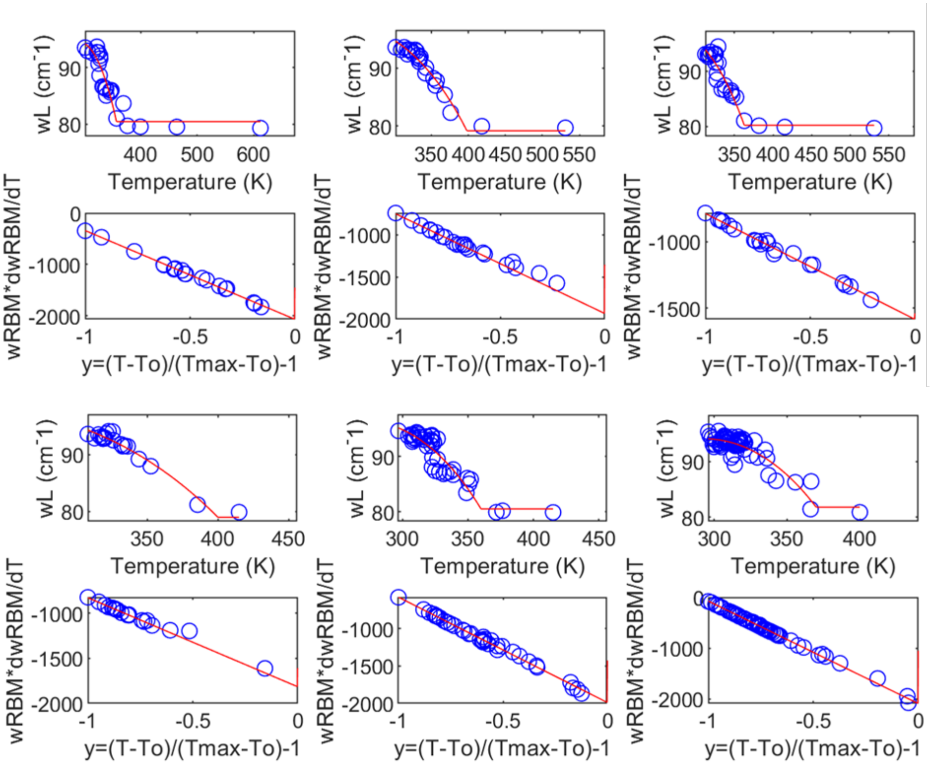

filename = ["Matthias_CNT-X_211222_210803Bs01x1177_10_2.5e-5mbar.csv"];

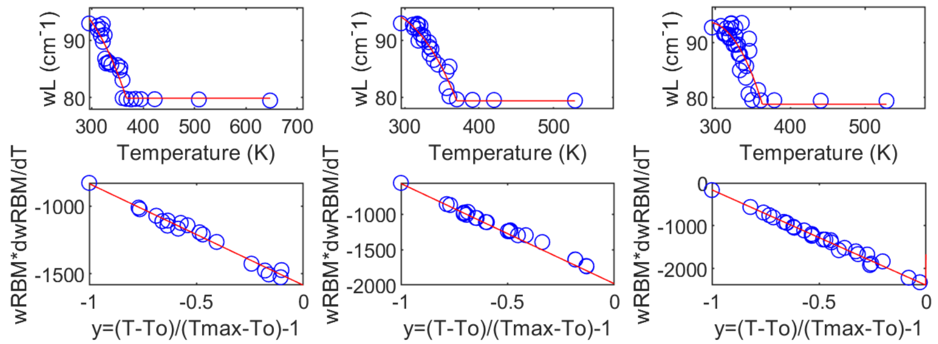

filename = ["Matthias_CNT-X_211223_210803Bs01x1177_11_2.5e-5mbar.csv"];

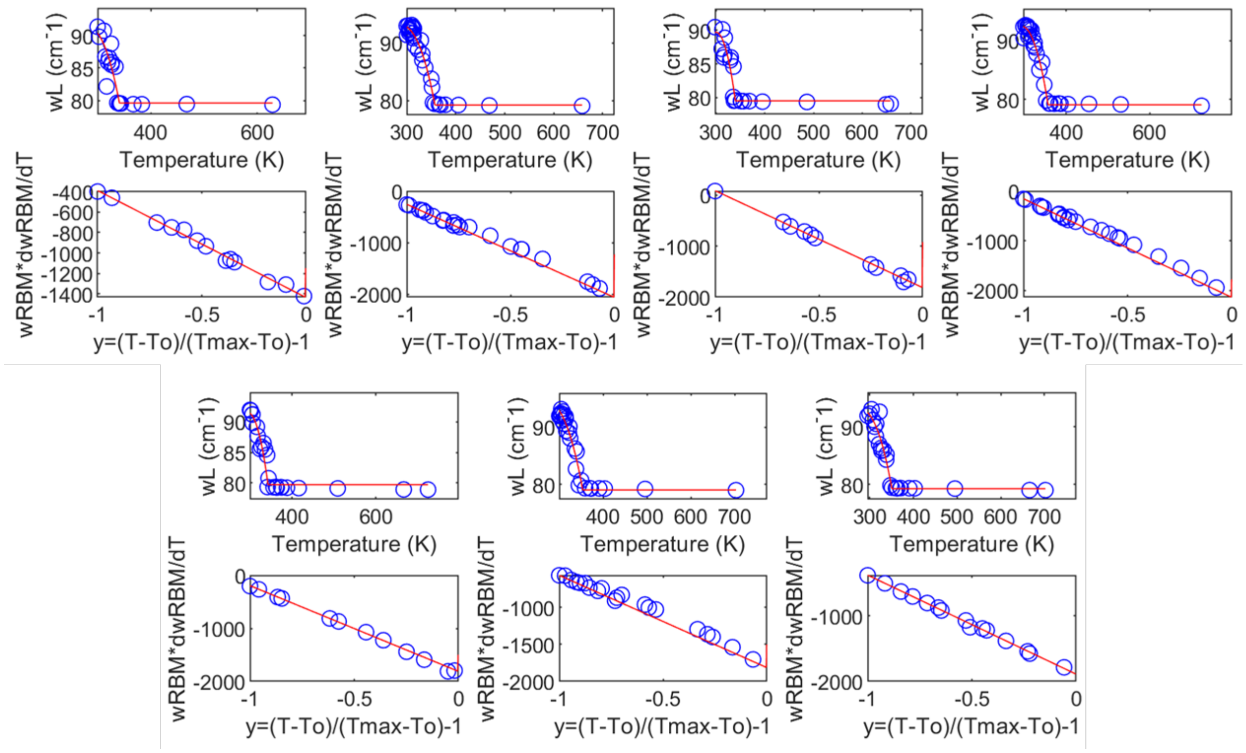

filename = ["Matthias_CNT-X_211228_210803Bs01x1177_12_1.9e-5mbar.csv"];

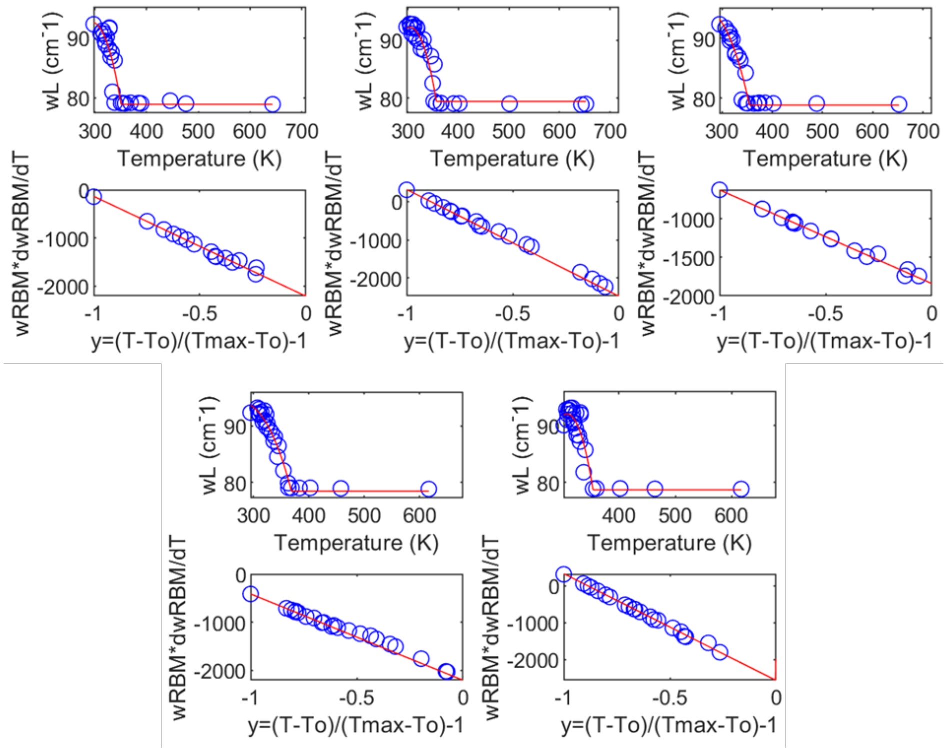


```
filename = ["Matthias_CNT-X_220105_210803Bs01x1177_13_1.6e-5mbar.csv"];
```

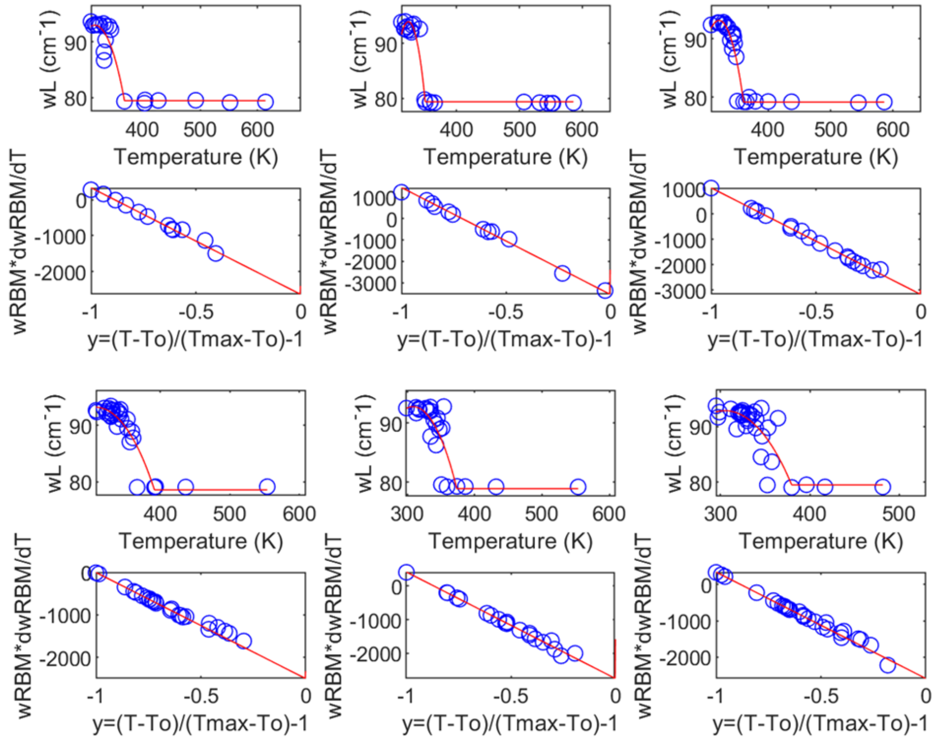

c) CNT F RBM Thermal Trajectories and Derivative Analysis

filename = ["210519_210518As01x1132_LaserPowerScan_633nm_p7.4e-4mbar.csv"];

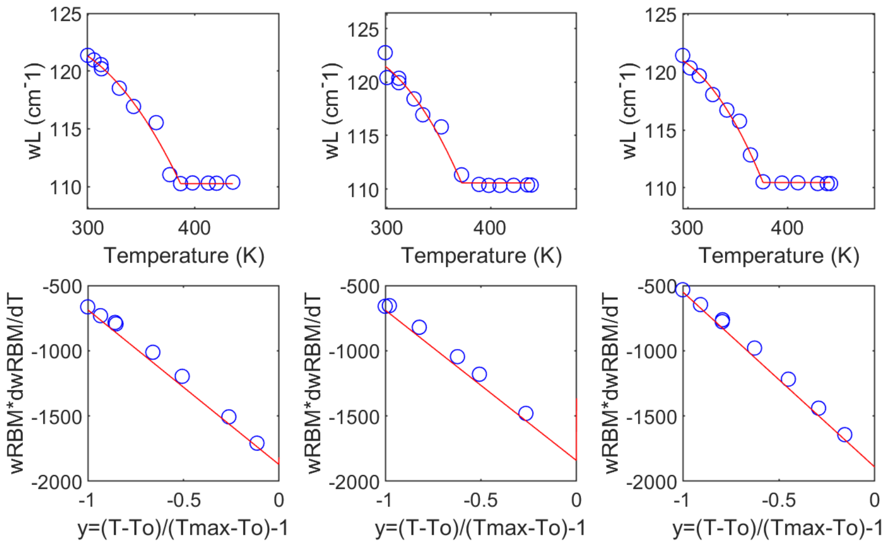

filename = ["210519_210518As02x1122_LaserPowerScan_633nm_p7.4e-4mbar.csv"];

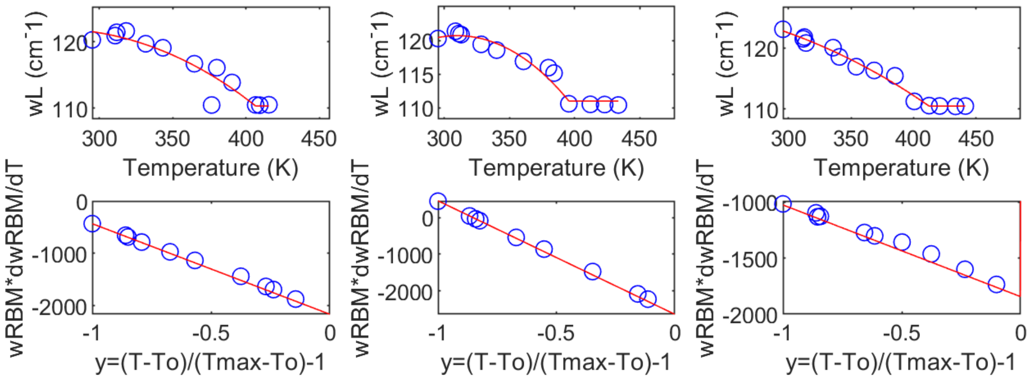

filename = ["210519_210518As03x1117_LaserPowerScan_633nm_p7.4e-4mbar.csv"];

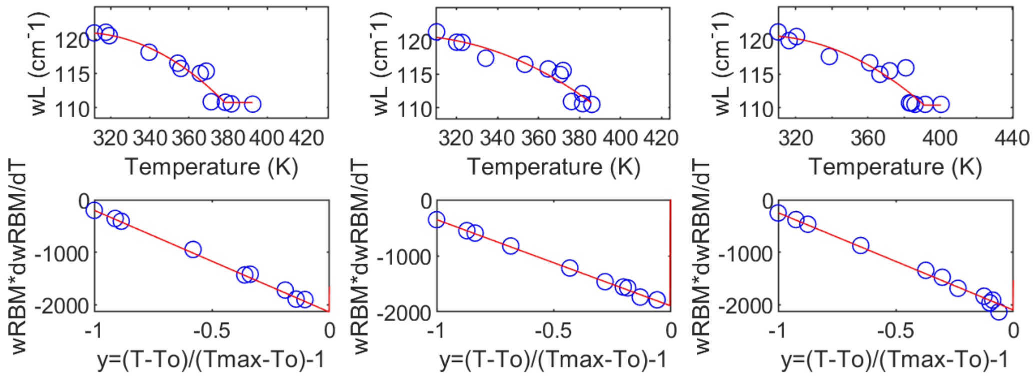

filename = ["210519_210518As04x1107_LaserPowerScan_633nm_p7.4e-4mbar.csv"];

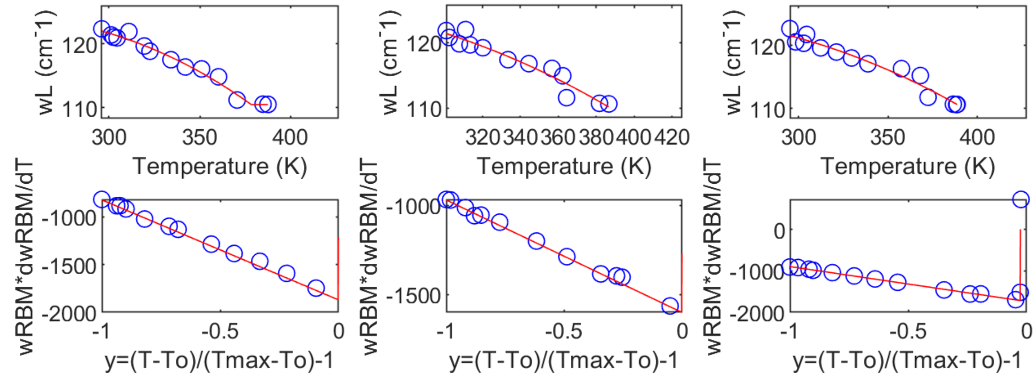

filename = ["210521_210518As01x1132_LaserPowerScan_633nm_p2.5e0mbar.csv"];

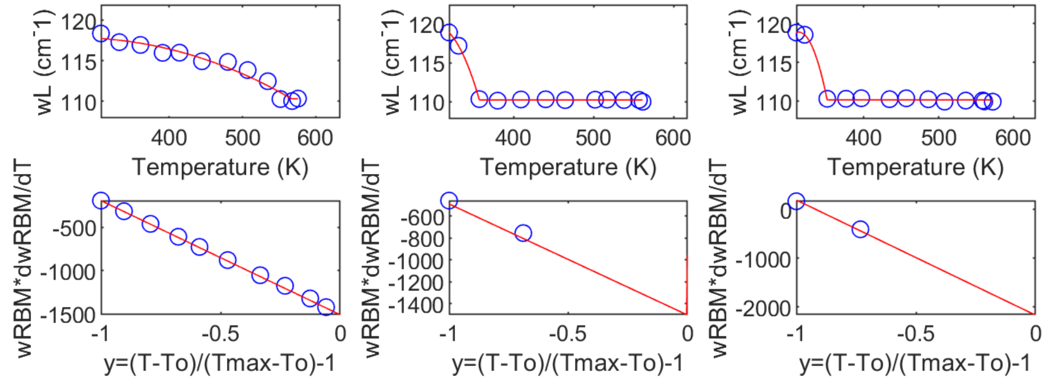

filename = ["210521_210518As02x1122_LaserPowerScan_633nm_p2.5e0mbar.csv"];

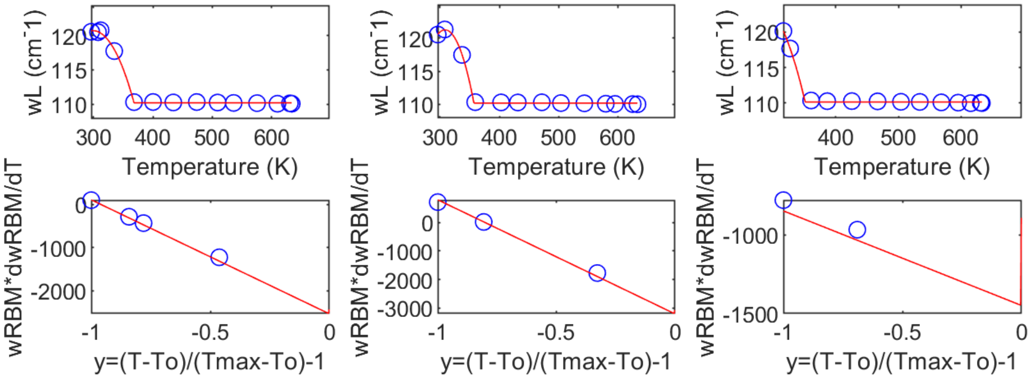

filename = ["210521_210518As03x1117_LaserPowerScan_633nm_p2.5e0mbar.csv"];

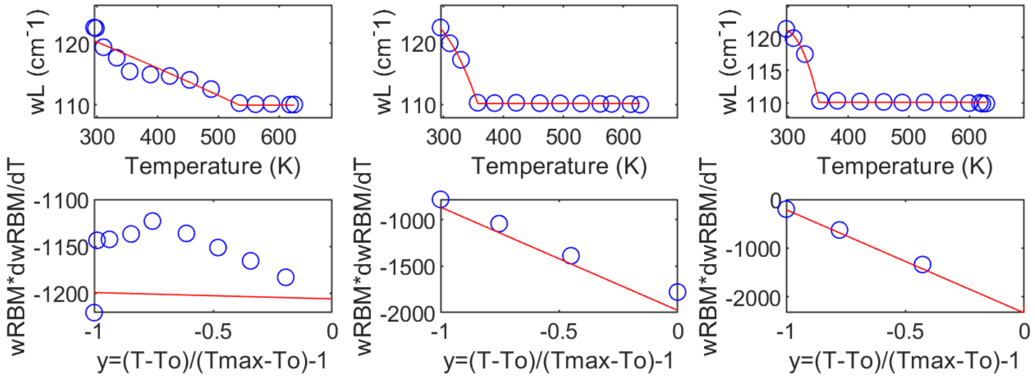

filename = ["210521_210518As04x1107_LaserPowerScan_633nm_p2.5e0mbar.csv"];

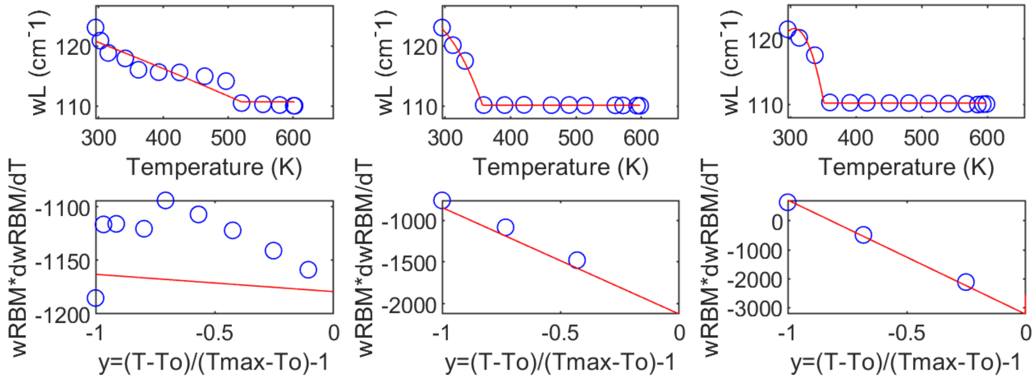

filename = ["210525_210518As02x1122_LaserPowerScan_633nm_p1.2e-3mbar.csv"];

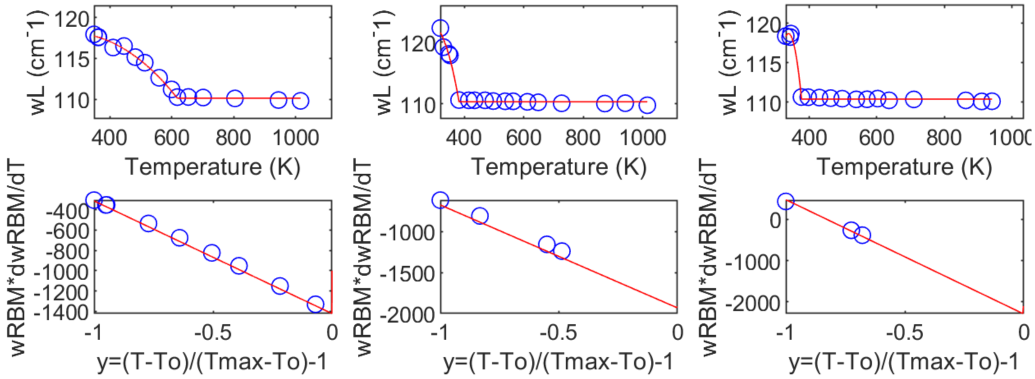

filename = ["210525_210518As03x1117_LaserPowerScan_633nm_p1.2e-3mbar.csv"];

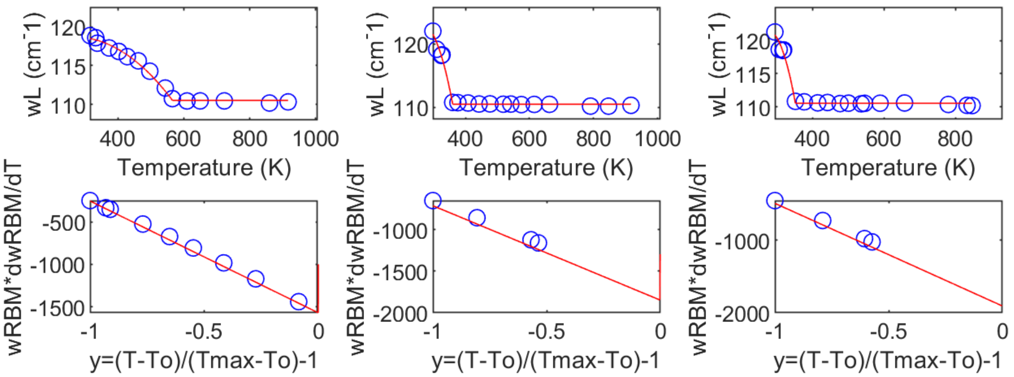

filename = ["210525_210518As04x1107_LaserPowerScan_633nm_p1.2e-3mbar.csv"];

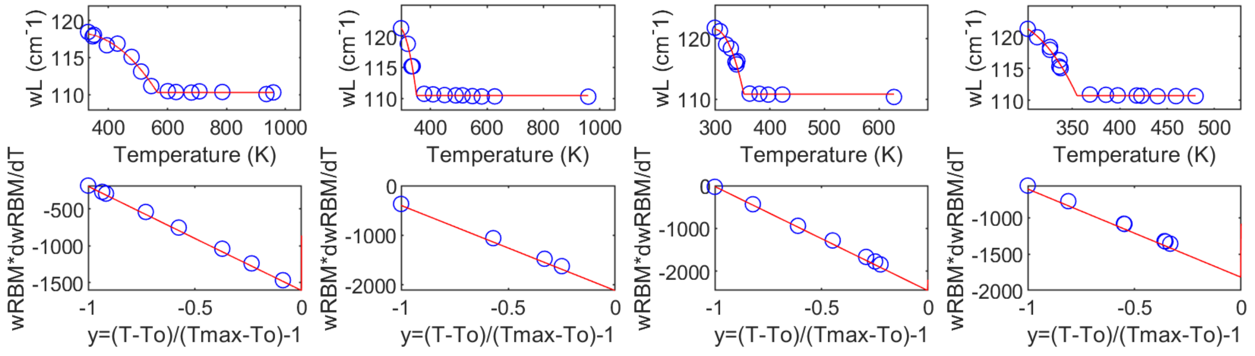

d) Rare Trajectories with Two Apparent T_{max} Values

Among the 93 RBM trajectories, three trajectories appear to show two distinct T_{max} values. These appear to be described by the sum of two terms represented by **Eq. (6)**. The result is **Supplementary Fig. 3-1** below:

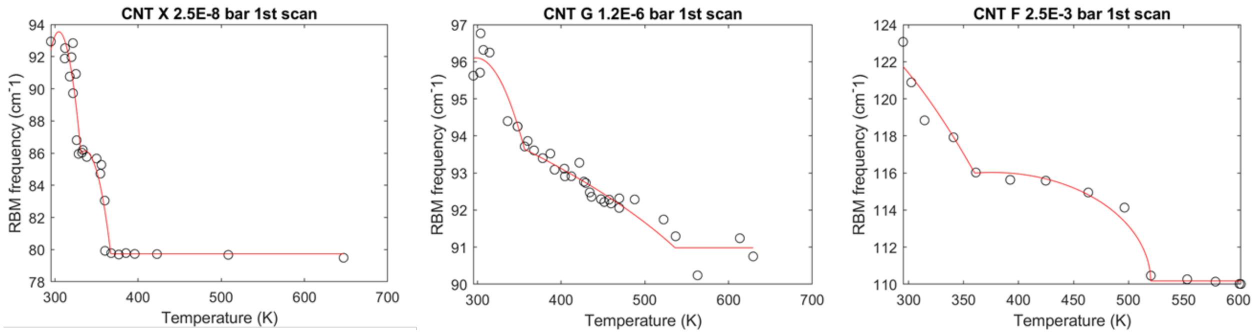

Supplementary Fig. 3-1| Three scans showing two T_{max} values.
Initial CNT X scan exhibiting T_{max} values at 331 and 367 K. Initial CNT G scan exhibiting T_{max} values at 356 and 536 K. Initial CNT F scan exhibiting T_{max} values at 360 and 520 K.

Mechanistically, since the heated spot is placed at approximately the center of the suspended CNT, there are two segments under tension on either side. If both segments relax the self-tension at approximately the same temperature, a single $T_{\max}$ value should be observed. In the cases in **Supplementary Fig. 3-1**, the two segments may relax at two distinct temperatures, creating the sum of two hyperbolic trajectories observed, each with a distinctly observable $T_{\max}$.

4: Summary of $T_{\max}$ Analysis of All RBM Trajectories for CNT G, X, and F

The figures below are the $T_{\max}$ values from the scans for each CNT in the same order as the files listed in the previous section. Dotted lines are the $T_{\max}$ estimate from the graphene self-tension value in the literature, CNT mechanical modulus coefficient of thermal expansion (CTE) $= 2 \times 10^{-5}$ K^{-1} for SWNT and DWNT, and a laser spot size of 2 μm, as discussed in the main text.

a) $T_{\max}$ values for each data file for CNT G

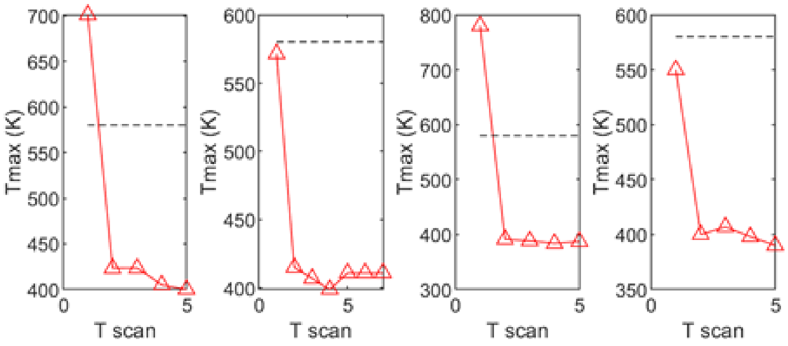

b) T_{max} values for each data file for CNT X

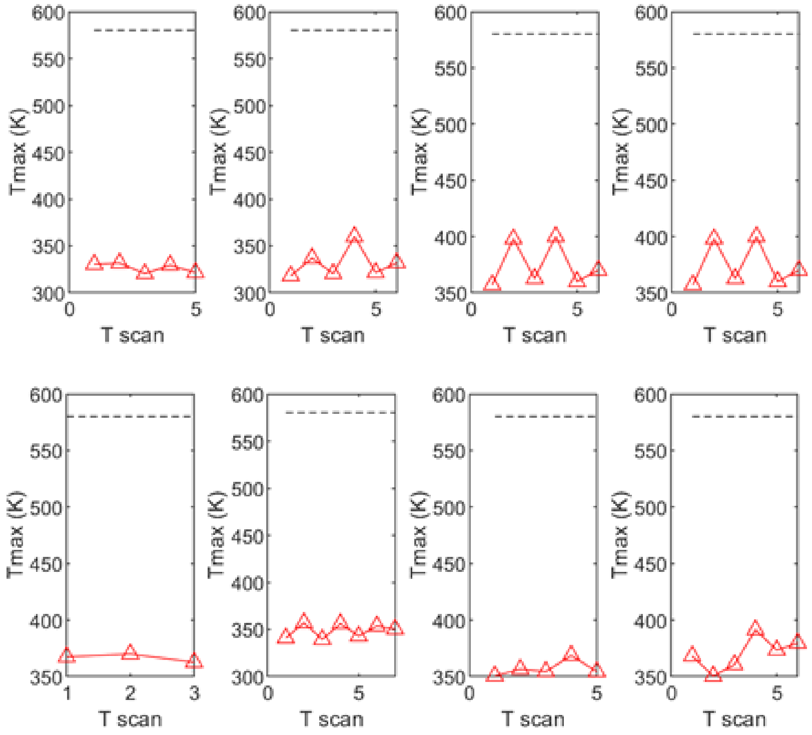

c) T_{max} values for each data file for CNT F

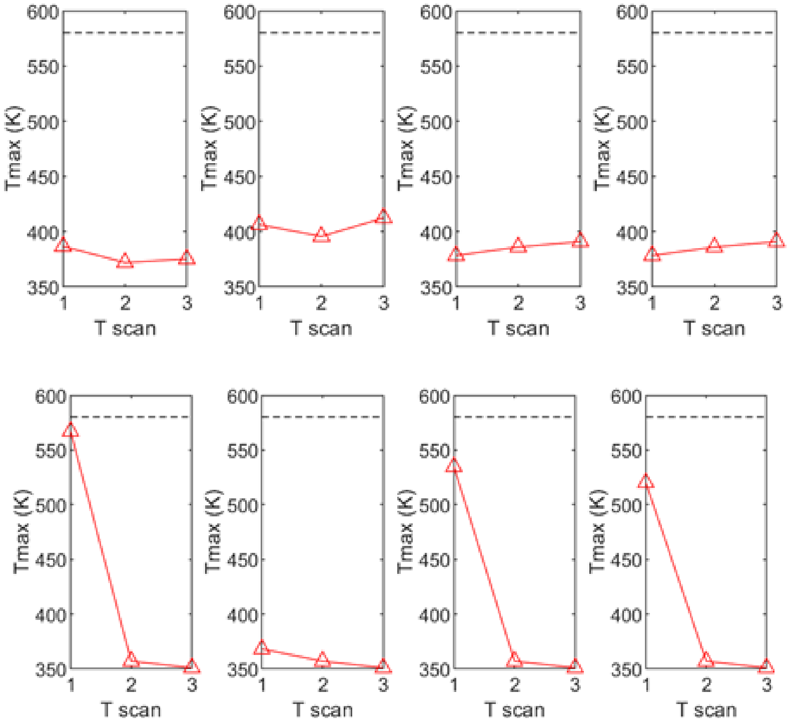

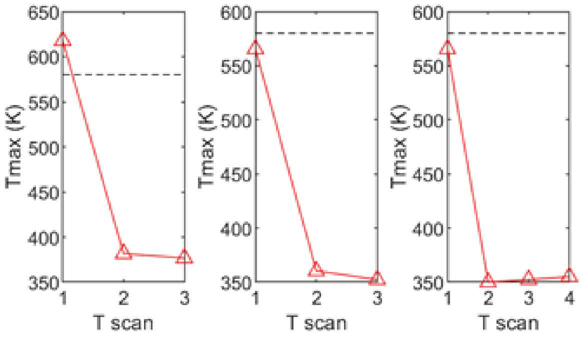

5: Transmission Electron Microscopy (TEM) of CNTs and Graphitic Impurities

Transmission electron microscopy (TEM) and electron diffraction (ED) of suspended CNTs were performed on a Thermo Fisher Themis Z G3 Cs-Corrected scanning transmission electron microscope (STEM). The microscope was operated in TEM mode at 60 kV, below the knock-on damage threshold for carbon nanotubes. A 100 μm objective aperture was used to improve image contrast and reduce delocalization. ED patterns were collected with a 20 nm diameter parallel beam on a Ceta CMOS camera.

a) Statistical information on CNTs produced from the CVD synthesis of this work

In order to draw generalizable conclusions, we gathered statistical information on the CNTs that resulted from our synthesis, by growing CNTs directly on commercial TEM grids (PELCO®, Ted Pella, Inc) with openings in a Si_3N_4 or silicon membrane much smaller in size than the slits in our homemade TEM chips. **Supplementary Fig. 5-1a** is a low-magnification image showing two CNTs on a SiN membrane with 2 μm-diameter holes over which CNTs are partially suspended. **Supplementary Fig. 5-1b** is a higher resolution image of such a CNT. Based on the image, we assign this tube as being isolated as opposed to being composed of two or more bundled tubes. Based on high-resolution images shown in **Supplementary Fig. 5-1c**, we further determine wall

number and inner diameter of each tube on these commercial TEM grids. **Supplementary Fig. 5-1d** to **f** show results obtained in this manner from CNTs grown on 15 TEM grids. In 65 % of cases, we found an isolated CNT, and in 35 % of cases we found a CNT bundle (**Supplementary Fig. 5-1d**). Constituent tubes differed in wall number, with 1-3 walls being typical (**Supplementary Fig. 5-1e**). Most frequent inner diameters were in the 1-2.5 nm diameter range. All TEM images collected of as-grown CNTs exhibit carbonaceous impurities on the CNT exterior at surface attached masses (**Supplementary Fig. 5-1b** and **c**).

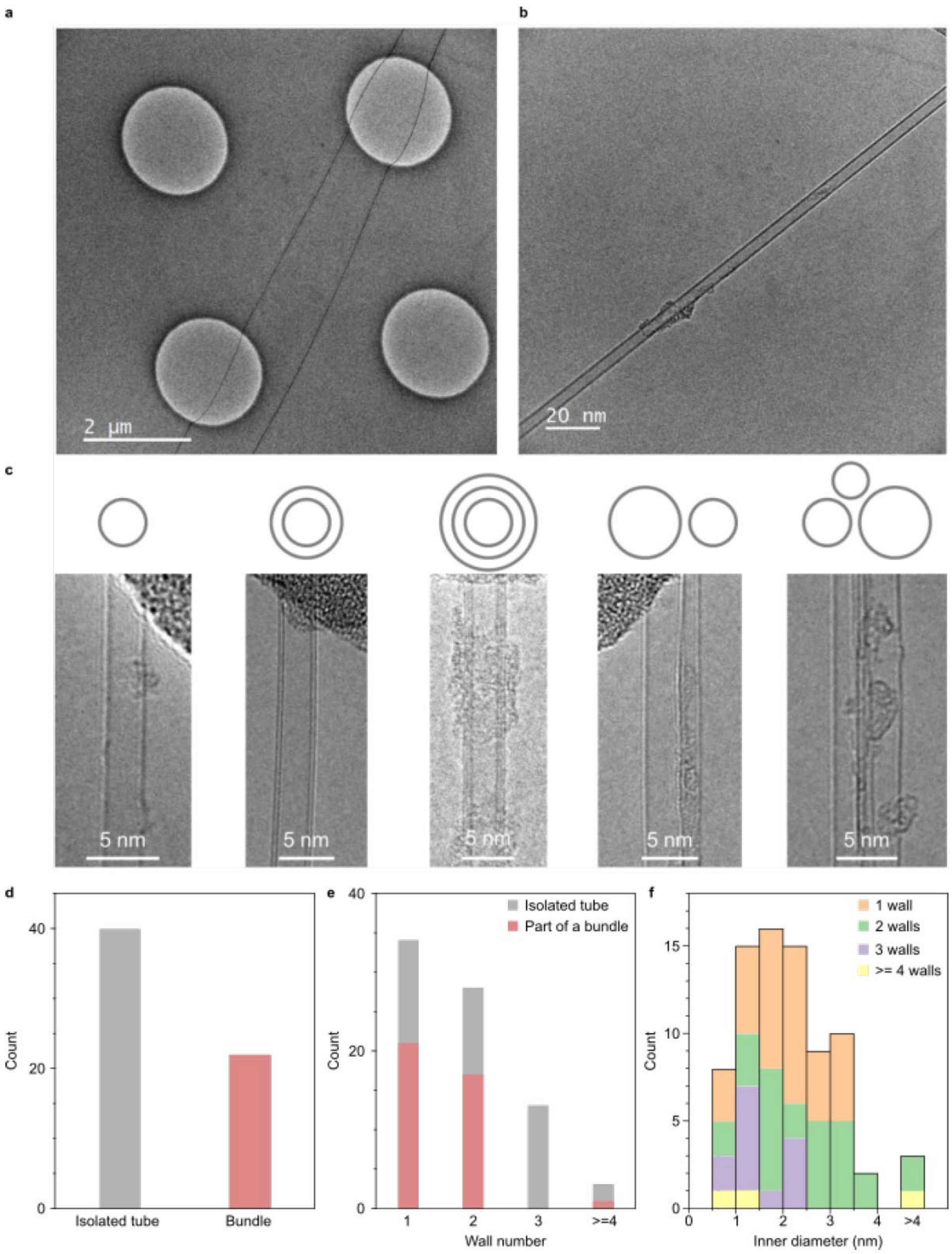

Supplementary Fig. 5-1| Transmission electron microscopy of as-grown CNTs on commercial TEM grids. **a**, Low-magnification TEM image shows two CNTs that are partially suspended over holes in a Si_3N_4 membrane. **b**, Higher magnification TEM image, reveals an isolated CNT as opposed to a CNT bundle. Some graphitic carbons, typical residues from the CNT synthesis, is observed on the CNT exterior. **c**, High-resolution TEM images (bottom row) that allow to determine the wall number of different CNTs. Top row: Tentative schematic cross-sections of CNTs shown in the bottom row. **d-f**, Histograms of **d**, isolated CNTs vs. bundles, **e**, wall number of all resolved tubes, include constituent tubes of bundles, and **f**, inner tube diameter, include of each resolved constituent tube of a bundle.

b) TEM Video of Graphitic Ribbons on a CNT and Mean Squared Displacement

To visualize and examine as-synthesized CNT and the vibrational motion of ribbons lattice on the CNT surface, we utilized Cs-corrected STEM operating at the acceleration voltage of 60 kV. As presented in the time still frame of TEM images (**Supplementary Fig. 5-2a** to **c**) and **Supplementary Movie 1**, we observed that the graphitic ribbons are mobile under the transmission electron beam and has the ability to lock into place on the CNT surface. The dynamics of graphitic ribbons have the potential to lattice register with the underlying CNT and influence the RBM frequency of a CNT. The mean squared displacement (MSD) of a graphitic ribbon movement (orange arrow) is estimated by a single-particle tracking with every 10 frames (t = ~5 s) (**Supplementary Fig. 5-2d**). The 1D diffusion is approximately 0.121 nm^2 s^{-1} by the linear fit of the first 20 data points.

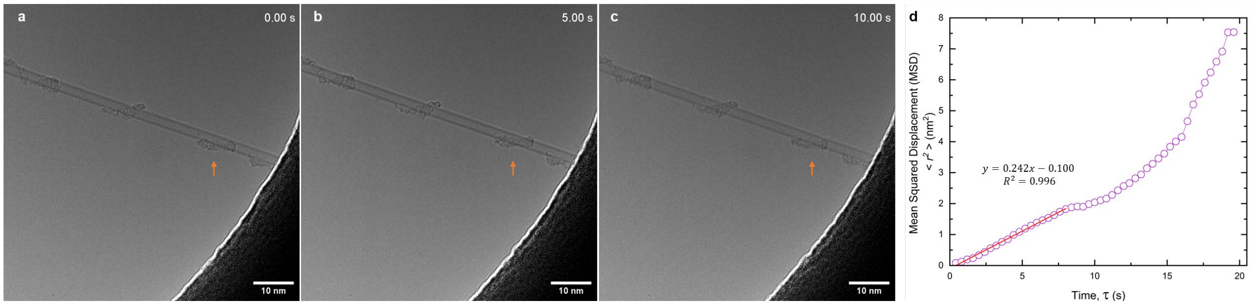

Supplementary Fig. 5-2| Representative TEM side-by-side still frame of graphitic ribbon motions on a CNT. **a**, at time = 0.00 s; **b**, at time = 5.00 s; **c**, at time = 10.00 s TEM images from **Supplementary Movie 1** show visual representation of the vibrational movement of the graphitic ribbons with the surface of a CNT under the electron beam. **d**, Individual trajectory and mean-squared displacement (MSD) of a graphitic ribbon (orange arrow). The linear fit (red line) is applied to the first 20 data points, $y = 0.242x - 0.100;\ R^2 = 0.996$.

6: Thermodynamic and Mathematical Proof Distinguishing Damping and Fluid Adsorption

As shown in **Fig. 1e** and **Supplementary Fig. 6-1**, all 93 temperature scans of the RBM frequency for all three DWNTs (CNT F, G and X) studied in this work exhibit a concave downward trajectory for $T < T_{\max}$ (for $T > T_{\max}$, the RBM remains invariant in all cases).

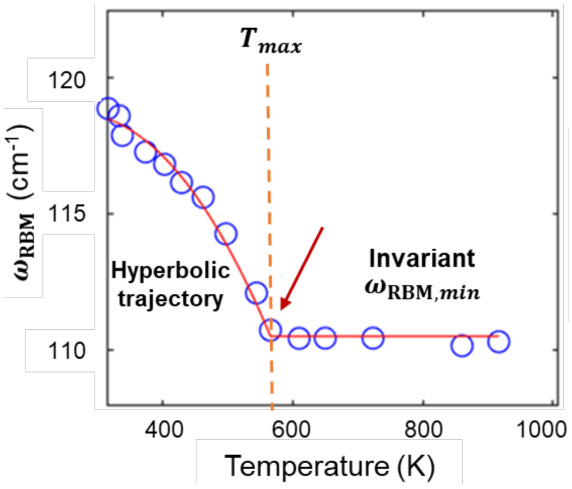

Supplementary Fig. 6-1| A representative plot of all 93 temperature scans.
All 93 Raman scans of CNT F, G, and X exhibits a concave down trajectory towards $T_{\max}$, and invariant $\omega_{\text{RBM,min}}$ at $T > T_{\max}$.

a) Falsifying the null hypothesis of an adsorbed fluid changing the restoring force at the CNT wall or net spring constant (γ), no damping term

Describing the RBM frequency using a temperature (T) dependent spring constant imposed by an adsorbed fluid ($\gamma_2[T]$) with a linear expression for the purpose of a simple mathematical and conceptual elucidation:

$$\omega_{\text{RBM}}[T] = \beta_2 + \gamma_2[T] \qquad \textbf{Eq. S6-1}$$

Here, β_2 is the RBM frequency in the absence of any environmental coupling (intrinsic spring constant of CNT).

The second derivative of the ω_{RBM} frequency with temperature is then:

$$\omega_{\text{RBM}}''[T] = \gamma_2''[T] \qquad \textbf{Eq. S6-2}$$

If the adsorbed fluid modifies the spring constant proportional to the surface coverage $q[T]$ manner, where $0 < q[T] < 1$,

$$\gamma_2[T] = \gamma_o\, q[T] \qquad \textbf{Eq. S6-3}$$

the Langmuir isobar relates the spring constant to the temperature via:

$$q[T] = \frac{e^{\frac{\Delta H}{RT}} P K_o}{1 + e^{\frac{\Delta H}{RT}} P K_o} \quad \textbf{Eq. S6-4}$$

where K_o is equilibrium constant, ΔH is heat of adsorption, and P is the fluid pressure. The second derivative of **Eq. S6-4** is then:

$$q''[T] = \frac{e^{\frac{\Delta H}{RT}} P \Delta H K_o (2RT + \Delta H + e^{\frac{\Delta H}{RT}} P (2RT - \Delta H) K_o)}{R^2 T^4 (1 + e^{\frac{\Delta H}{RT}} P K_o)^3} \quad \textbf{Eq. S6-5}$$

In the limit of high temperature, as in the approach to $T_{\max}$, noting that exp(ΔH/RT) ~ 1 in this limit, assuming that the high temperature limit is observed, the **Eq. S6-5** becomes:

$$q''[T] = \frac{2P\Delta H K_o}{RT^3 (1 + PK_o)^2} > 0 \quad \textbf{Eq. S6-6}$$

We note that for all fluids adsorbing to the surface, ΔH must be > 0, and all other quantities on the right-hand side (RHS) of **Eq. S6-6** are > 0. Because it is impossible for the RHS to be < 0, contrary to what we observe experimentally in this work, this conclusively rules out fluid adsorption/desorption as the mechanism for the RBM trajectory. Additionally, the first derivative of **Eq. S6-4**:

$$q'[T] = -\frac{e^{\frac{\Delta H}{RT}} P K_o \Delta H}{R\left(T + e^{\frac{\Delta H}{RT}} P T K_o\right)^2} < 0 \quad \textbf{Eq. S6-7}$$

$q'[T]$ must always be less and never equal to zero at finite temperature, contrary to what we observe for $T > T_{\max}$ for the trajectories in question. While the above limit is analytically derived for the Langmuir isobar, it remains true for all other adsorption models which converge to the Langmuir limit as $T \rightarrow \infty$. Because of this, we exclude the possibility that reversible fluid adsorption/desorption is the cause of the temperature dependent trajectory discussed in the first part of the main text (i.e., in the absence of interior water filling). The mathematical proof in this

section is experimentally confirmed when the same CNT system is subjected to FIB cut opening, humidity filling with water, and sealed (**Fig. 5** in the main text.). After this process, approximately 2/3 of the corresponding temperature trajectories of the RBM show a distinct, concave up (positive $q''[T]$) trajectory consistent with the Langmuir adsorption process.

b) The downward concavity of the RBM trajectory requires a negative 2nd derivative

Allowing for a generic temperature dependence of $\gamma[T]$ and $b[T]$:

$$\omega_{\mathrm{RBM}}[T] = \sqrt{\frac{\beta}{r^2} + \gamma[T] - b[T]^2} \qquad \textbf{Eq. S6-8}$$

The second derivative becomes:

$$\omega_{\mathrm{RBM}}''[T] = \frac{-b'[T]^2 - \omega_{\mathrm{RBM}}'[T]^2 - b[T]c + \gamma''[T]/2}{\omega_{\mathrm{RBM}}[T]} \qquad \textbf{Eq. S6-9}$$

Where we note that if $b[T]$ is approximately linear with temperature as observed in this work, $b''[T] = \text{constant} = \mathrm{c} < 0$. Note that $\gamma''[T]$ cannot be < 0 if attributed to fluid adsorption or desorption as per the proof in **Supplementary Text 6: a** above; hence $b[T]$ cannot be zero. Also, the inclusion of the damping term in **Eq. (2)** additionally rules out a fluidic description, as prevailing theory is built around $b = 0$ for this case[1]. Therefore, the experimental trajectories in this work that all have $\omega_{\mathrm{RBM}}''[T] < 0$ are attributed to a change in the coupling of a stationary mass with respect to the CNT (via damping of the CNT radial velocities), as in the case of the graphitic ribbons observed in this work.

If the spring constants of the stationary mass are invariant and $b[T]$ c << $b'[T]^2$:

$$\omega_{\mathrm{RBM}}''[T] = \frac{-b'[T]^2 - \omega_{\mathrm{RBM}}'[T]^2}{\omega_{\mathrm{RBM}}[T]} \qquad \textbf{Eq. S6-10}$$

Note that the RHS in this limit is necessarily < 0 for all T, regardless of the dependence of whether $b[T]$ is monotonically increasing or decreasing. This means that the observation that:

$$\omega_{\mathrm{RBM}}''[T] < 0 \qquad \textbf{Eq. S6-11}$$

under these assumptions is a characteristic property of a change in environmental damping as the mechanism inducing the ω_{RBM} change with any experimental variable. This observation has the utility of determining the source of environmental coupling affecting the CNT.

7: Use of the Single Shell Approximation versus Double Coupled Shells

To simplify the theory, **Eqs. (6)** and **(8)** in the main text were derived by modeling the DWNT as a single shell oscillating at the low RBM frequency (outer shell, $\omega_{\mathrm{RBM,L}}$). This section shows that this approximation is expected to introduce less than 1 cm^{-1} and 2 cm^{-1} error for $T > T_{\mathrm{max}}$ and $T < T_{\mathrm{max}}$, respectively. Future work will refit all RBM trajectories at high and low frequency (inner and outer shells) to a more complex coupled elastic shell model and obtain more accurate parameters. This is not expected to change the conclusions of this work, however.

To determine the error bound, we compared the Single Shell Approximation used in the main text to the case of two explicitly coupled shells. If $w[t]$ is the radial displacement of the outer shell, environmentally coupled to the external carbon with damping constant b (temperature dependent), and now coupled to an inner shell with radial displacement $z[t]$, the coupled equations become:

$$\left(\frac{\beta}{r^2} + \gamma\right) w[t] + \frac{\bar{b}}{2\rho} w'[t] + w''[t] = \omega_{\mathrm{o}}^2 w[t] + 2b w'[t] + w''[t] \; - \gamma_{\mathrm{cc}}\, z[t] = 0 \qquad \textbf{Eq. S7-1}$$

$$\omega_{\mathrm{oz}}^2\, z[t] + z''[t] \; - \gamma_{\mathrm{cc}}\, w[t] = 0 \qquad \textbf{Eq. S7-2}$$

Here, γ_{cc} is the spring constant of the coupled shells, which can be incorporated into the composite spring constants of the outer shell ω_{o}^2 and inner shell ω_{oz}^2 respectively.

The high frequency RBM corresponding to the coupled inner shell can be modeled by solving the two equations above using the previously fitted ω_{o}^2 and $b[t]$ from the Single Shell approximation used in the main text and adjusting ω_{oz}^2 and γ_{cc} to describe the coupled trajectory in the high RBM frequency mode (inner shell, $\omega_{\mathrm{RBM,H}}$). **Supplementary Fig. 7-1** compares three example scans from the CNT X data set. The parameters ω_{oz}^2 and γ_{cc} are adjusted to fit the $\omega_{\mathrm{RBM,H}}$ of the first scan, but left at these values for subsequent comparisons (**Supplementary Fig. 7-1, b**) and (**Supplementary Fig. 7-1, c**). We find that for the low RBM frequency (outer shell, $\omega_{\mathrm{RBM,L}}$) trajectories, the difference between Single Shell and coupled shell models is less than 2 cm^{-1} generally, with the concavity and cusp still well described. This provides confidence that the analysis performed in the main text, yielding estimates of damping and spring constant frequencies, have sufficient accuracy. The comparison of the $\omega_{\mathrm{RBM,H}}$ trajectories are also well described by the coupled model, still employing the Single Shell parameters from the main text. Two fit parameters for the first scan are able to adequately describe subsequent scans. Hence, these results indicate that the Single Shell approximation employed in the main text is sufficient for the analysis therein.

Future work will refit both trajectories with the more general double shell model above, and provide information about γ_{cc} and its temperature dependence. The question of whether the inner shell frequency contribution is also dampened will be addressed by comparison to the extensive 93 scan data set we have collected in this work for three DWNTs.

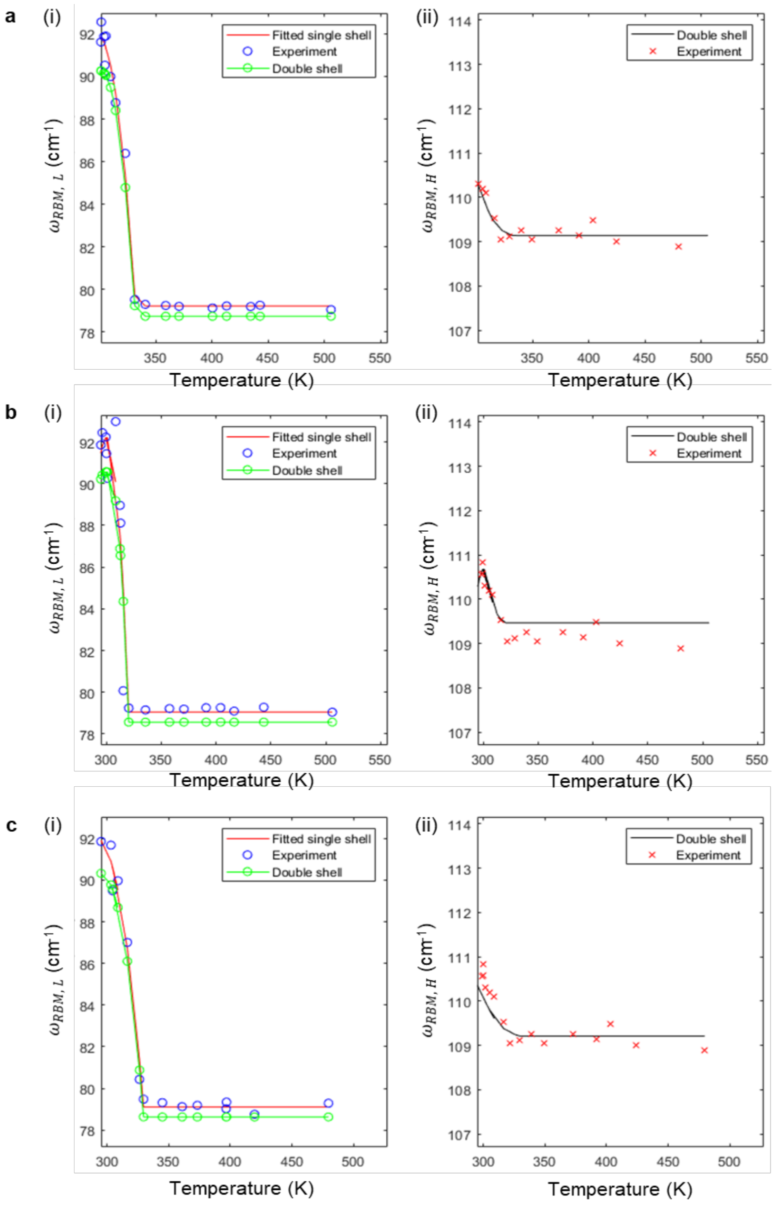

Supplementary Fig. 7-1| Extension of the Single Shell Approximation to the Double Coupled Shell Model. The parameters fit from the Single Shell Approximation for a series of scans from CNT X can be used in a Double Coupled Shell Model without modification to describe the high RBM frequency ($\omega_{RBM,H}$) associated with the inner shell. The shell coupling and low RBM frequency (outer shell, $\omega_{RBM,L}$) (**a, i**) are fit to the $\omega_{RBM,H}$ scan in (**a, ii**), which also compares the two models to the experimental data for $\omega_{RBM,L}$. The $\omega_{RBM,L}$ changes less than 2 cm^{-1} between the two models, whereas $\omega_{RBM,H}$ is adequately described. The subsequent scan in **b**, uses the same parameters and is shown to describe both outer (**b, i**) and inner (**b, ii**) shell frequencies. Scan **c**, is similarly described by the same parameters in the next scan. The results indicate that the Single Shell Approximation used in the main text is not expected to introduce significant errors. Future work will refit all of the RBM trajectories for inner and outer shell contributions using the more complex Double Coupled Shell model to elucidate the inter-shell coupling. The scans are from the data set in (*Matthias_CNT-X_211113_210803Bs01x1177_02_2e-2mbar.csv*).

8: Full Derivation of the RBM Frequency from the Harmonic Oscillator Model

a) Single shell force balance:

The radial force balance on the CNT that describes the uniaxial radial displacement in time ($w[t]$) is given by:

$$\left(\frac{\beta}{r^2}+\gamma\right)w[t]+\frac{\bar{b}}{2\rho}w'[t]+w''[t]=\omega_o^2 w[t]+2bw'[t]+w''[t]=0 \qquad \textbf{Eq. S8-1}$$

The solution to **Eq. S8-1** yields the RBM frequency (ω_{RBM}) as

$$\omega_{\mathrm{RBM}}[T]=\sqrt{\omega_o^2-b^2} \qquad \textbf{Eq. S8-2}$$

where b is the damping coefficient, and $\omega_o^2=\frac{\beta}{r^2}+\gamma$ is the sum of the intrinsic CNT and environmental coupling spring constants. Note that here, for simplicity, we have modeled the DWNT as a single shell with a single frequency as the lower value of the two from the coupled system. The error this introduces (< 2 cm^{-1}) is examined in a later **Supplementary Text 7:**.

b) A linear damping, increasing with temperature appears experimentally justified

The experimental observation of the $T_{\max}$ value (temperature, T at the minimum RBM frequency, $\omega_{\mathrm{RBM,min}}$) suggests the scaling below:

$$y[T]=-1+\frac{T-T_o}{T_{\max}-T_o} \qquad \textbf{Eq. S8-3}$$

where T_o is room temperature.

The main text and **Supplementary Text 7:** above provide the motivation for the focus on the damping coefficient b variation with $y[T]$ but the functional form of the temperature dependence is not obvious. Squaring both sides of **Eq. S8-2** and taking the derivative with respect to T yields:

$$\omega_{\mathrm{RBM}}[T]\,\omega_{\mathrm{RBM}}'[T] = -b\big[y[T]\big]\,b'\big[y[T]\big]\,y'[T] \qquad \textbf{Eq. S8-4}$$

Plotting the experimental trajectory on the left-hand side (LHS) against $y[T]$ appears to yield a negative trend for all values of $T < T_{\mathrm{max}}$ (**Fig. 3c-e**, and **Supplementary Text 3:** above applied to all scans in the data set). Therefore, damping must be increasing with T because $b[T] > 0$ for all $y[T]$ and:

$$y'[T] = \frac{1}{T_{\mathrm{max}} - T_{\mathrm{o}}} > 0 \qquad \textbf{Eq. S8-5}$$

The LHS requires that:

$$\omega_{\mathrm{RBM}}[T]\,\omega_{\mathrm{RBM}}'[T] = -(b[y[T]]^2)' \qquad \textbf{Eq. S8-6}$$

And equal to a linear function in $y[T]$ and therefore T. On integration, one obtains that $b[T]$ should be a linear expansion in T. If $b[T]$ is:

$$b[T] = a + g\,y[T] \qquad \textbf{Eq. S8-7}$$

Then:

$$\omega_{\mathrm{RBM}}[T]\,\omega_{\mathrm{RBM}}'[T] = \pm\, g\,(a + g\,y[T])\,y'[T] \qquad \textbf{Eq. S8-8}$$

A further consequence of this linearity is that damping must be increasing with increasing T since $b[T] > 0$ for all $y[T]$ and **Eq. S8-5**.

Mathematically, $b'[T]$ must therefore always be greater than zero. Therefore, the negative second derivative in the experimental data means that the damping constant is increasing with temperature over the course of the trajectory. This analysis also allows one to use the linearity of the plot in each case to justify a linear expansion of the trajectory variable ($b[T]$).

c) Scaling Requirements with Temperature for the Spring Constant to Describe RBM Trajectories

If the environmental spring constant (γ) of the ribbons were changing between strongly bound (at T_0) to weakly bound (at $T_{\max}$) states, the decay in value would have to be stronger than T^n with $n > 1$. The proof of this follows. Assuming a polynomial expansion in T:

$$\gamma = c_1 - c_2 T^n \qquad \textbf{Eq. S8-9}$$

The first derivative of **Eq. S8-9** must always be negative as observed:

$$\frac{d\gamma}{dT} = -c_2\, n\, T^{n-1} \qquad \textbf{Eq. S8-10}$$

Hence, $c_2 > 0$.

The second derivative of **Eq. S8-9** yields

$$\frac{d^2\gamma}{dT^2} = -c_2\, n\, (n-1)\, T^{n-2} \qquad \textbf{Eq. S8-11}$$

Hence, it would be required for $n > 1$ to yield both a negative second derivative and the concave down trajectory observed in this work, and a decrease (negative first derivative) towards $T_{\max}$. The strength of this scaling is non-physical for such spring constant softening. Moreover, the magnitude of the ω_{RBM} change significantly exceeds the bounds between a tightly packed CNT bundle and the nominal pristine vacuum state[2]. This allows us to rule out a temperature dependent spring constant change, and focus on a change in the damping term in **Eq. (2)** (**Eq. S8-1**).

9: The Magnitude of the RBM Trajectories is Consistent with Damping

The magnitude and direction of the shift are consistent with previous estimates of the specific damping for CNTs. The outer shell of CNT X (28,11) has a diameter calculated to be 2.73 nm and a number of atoms in the unit cell of 4,852. The mass per carbon atom of

2.0×10^{-23} g/atom yields a specific axial mass of 6.5×10^{-15} kg m^{-1}. CNT X exhibits an estimated change in frequency damping of $(\omega_{\mathrm{RBM,max}})^2 - (\omega_{\mathrm{RBM,min}})^2 = 2{,}408\ \mathrm{cm}^{-2}\ or\ 49\ \mathrm{cm}^{-1}$, since $(\omega_{\mathrm{RBM,max}})^2 - (\omega_{\mathrm{o}})^2 = (\Delta b)^2$. This predicts 9.6 mN s m^{-2} as the specific damping, in close agreement with the value of 8.3 mN s m^{-2} calculated by Cronin *et al.*[3] for smaller diameter SWCNT.

Similar calculations for CNT F (outer shell (22,11)) yield 8.2 mN s m^{-2}. For CNT G (outer shell (35,6)) the calculated value is 9.5 mN s m^{-2}. This motivates a focus on a change in the damping term in **Eq. (2)** (**Eq. S8-1**) above as the source of the trajectories.

According to existing theory, damping in the system rules out fluid adsorption or desorption as a mechanism for the trajectories, since fluids are modeled with negligible damping[1]. This is consistent with the mathematical findings in **Supplementary Text 6: b** above.

10: Strain-induced coupling and damping of the RBM oscillator

This section contains the full derivation of the strain-dependent coupling model introduced in the main text. The suspended CNT across the TEM window necessarily becomes self-tensioned due to contact between the edges of the substrate window and the CNT, which pulls the CNT towards the window edges. The effect results in the stretching of the CNT by a distance L_{P} from its natural equilibrium distance, thereby producing an initial tension, Tn_{o}:

$$Tn_{\mathrm{o}} = ML_{\mathrm{P}} \qquad \textbf{Eq. S10-1}$$

Here, M is the CNT mechanical modulus.

The laser spot of size S heats the CNT to a temperature T, causing a thermal expansion L_{exp} governed by the Coefficient of Thermal Expansion (CTE), α, where T_{o} is room temperature.

$$L_{\mathrm{exp}} = S\alpha(T - T_{\mathrm{o}}) \qquad \textbf{Eq. S10-2}$$

This thermal expansion increases the equilibrium length of the CNT, reducing the tension Tn according to **Eq. S10-1** and **Eq. S10-2**:

$$Tn = M(L_{\mathrm{P}} - S\alpha(T - T_{\mathrm{o}})) \quad \textbf{Eq. S10-3}$$

The damping coefficient responds to the relaxed tension by reaching a maximum value, $b_{\max}$, from a minimally damped $b_{\min}$ at Tn_{o}. This switch between weak and strong damping can be governed by the lattice registration of the graphitic impurity that is facilitated at $Tn = 0$, compared to its frustration at Tn_o. The temperature-dependent damping coefficient is expressed as follows:

$$b[T] = b_{\max} - \frac{Tn(b_{\max} - b_{\min})}{Tn_{\mathrm{o}}} \quad \textbf{Eq. S10-4}$$

Here, Δb is defined as follows:

$$\Delta b = b_{\max} - b_{\min} \quad \textbf{Eq. S10-5}$$

In terms of the CTE from **Eq. S10-1** and **Eq. S10-3**, the expression becomes:

$$b[T] = b_{\max} - \Delta b\left(1 - \frac{S\alpha(T - T_{\mathrm{o}})}{L_{\mathrm{P}}}\right) \quad \textbf{Eq. S10-6}$$

This **Eq. S10-6** provides a quantitative theory for the $T_{\max}$ value:

$$T_{\max} - T_{\mathrm{o}} = \frac{L_{\mathrm{P}}}{S\alpha} \quad \textbf{Eq. S10-7}$$

Combining **Eq. S10-6** and **Eq. S10-7**:

$$b[T] = b_{\max} - \Delta b\left(1 - \frac{T - T_{\mathrm{o}}}{T_{\max} - T_{\mathrm{o}}}\right) \quad \textbf{Eq. S10-8}$$

The final expression for the temperature-dependent ω_{RBM} from **Eq. S8-2** and **Eq. S10-8** is as follows:

$$\omega_{\mathrm{RBM}}[T] = \sqrt{\omega_{\mathrm{o}}^2 - \left(b_{\max} + \Delta b\left(-1 + \frac{T - T_{\mathrm{o}}}{T_{\max} - T_{\mathrm{o}}}\right)\right)^2} \quad \textbf{Eq. S10-9}$$

The above equation suggests a natural scaling for temperature as **Eq. S8-3**.

We note that in the analysis of the G-peak frequency (ω_{G}) with scan temperature, the cusp at T_{max} is not observed. Rather, an overall linear dependence is seen over the scan range (and used to linearly calibrate the absolute temperature scale as usual). Generally, ω_G is expected to be a function of both temperature T and strain ε, so that:

$$d\omega_{\mathrm{G}} = \frac{\partial \omega_{\mathrm{G}}}{\partial T} dT + \frac{\partial \omega_{\mathrm{G}}}{\partial \varepsilon} d\varepsilon \qquad \textbf{Eq. S10-10}$$

In the literature[4-6], the first and second terms on the RHS have been separately measured and modeled as linear expansions, but not yet under conditions where both T and ε systematically vary simultaneously. In the most general case, the partial derivatives in **Eq. S10-10** are not orthogonal, and notably $\frac{\partial \varepsilon}{\partial T}$ for the CNT is decidedly non-zero, and equal to α, measured only recently[4]. Here, α necessarily cross correlates these derivatives in an environmentally dependent manner, requiring individual temperature calibration of each CNT studied as we have done in this work. The calibration done for $T < T_{\mathrm{max}}$ in this work for CNT X, F and G necessarily incorporates the effect of strain. For these reasons, the general proportionality we observe: $\omega_{\mathrm{G}} \propto (T - T_{\mathrm{o}})$ through the cusp at T_{max} is expected to provide an accurate measure of the temperature within the laser spot, even if $\frac{\partial \omega_G}{\partial T}$ and $\frac{\partial \omega_G}{\partial \varepsilon}$ are commensurate in magnitude and coupled by α when evaluated at T_{max}.

11: Understanding the Limiting $\boldsymbol{\omega_{\mathrm{RBM}}}$ when $\boldsymbol{T > T_{\mathrm{max}}}$

When $T > T_{\mathrm{max}}$, the ω_{RBM} remains constant with respect to T at a remarkably consistent limit for each CNT regardless of the position along the suspended CNT, the direction of heating, or the scan number in the series.

a) Derivation of the limiting $\boldsymbol{\omega_{\mathrm{RBM}}}$ when $\boldsymbol{T > T_{\mathrm{max}}}$

The strain-dependent coupling model predicts this limiting value for $\omega_{\mathrm{RBM,min}}$ as:

$$\omega_{\mathrm{RBM,min}} = \sqrt{\omega_{\mathrm{o}}^2 - b_{\mathrm{max}}^2} \qquad \textbf{Eq. S11-1}$$

It is reasonable to expect different surface densities of graphitic carbon from spot to spot or changes in the coverage over time, including upon thermal processing.

To understand the consistency of this $\omega_{\mathrm{RBM,min}}$ limit for a given CNT, we can note that the presence of graphitic impurities necessarily contributes to the damping by coupling and similarly increase the restoring force through van der Waals (vdW) contact, thereby affecting the spring constant. In other words, each ribbon contributes a differential increase (from spring constant) and decrease (from damping) to the RBM frequency. As the surface density of graphitic ribbons (n) axially vary, the net change to the RBM frequency remains constant.

We can model this phenomenon quantitatively as each impurity contributing a variable number of tethers, n, which in turn contribute γ_i to the spring constant and $b_{\mathrm{max},i}$ to the maximum damping on a per CNT mass basis such that:

$$\gamma = n^2\gamma_i \qquad \textbf{Eq. S11-2}$$

and

$$b_{\mathrm{max}} = nb_{\mathrm{max},i} \qquad \textbf{Eq. S11-3}$$

Note here that both $\sqrt{\gamma_i}$ and $b_{\mathrm{max},i}$ have units of cm^{-1} per tether and quantify the frequency change with each (positive for the former and negative for the latter).

As the surface coverage of impurities varies with n, the limiting RBM remains invariant according to:

$$\omega_{\mathrm{RBM,min}} = \sqrt{\left(\frac{\beta}{r^2} + n^2\gamma\right) - n^2 b_{\mathrm{max},i}^2} = \sqrt{\omega_{\mathrm{o}}^2 - b_{\mathrm{max}}^2} \quad \textbf{Eq. S11-4}$$

Taking the square of both sides of **Eq. S11-4**:

$$\left(\omega_{\mathrm{RBM,min}}\right)^2 = \omega_{\mathrm{o}}^2 - b_{\mathrm{max}}^2 = \left(\frac{\beta}{r^2} + n^2\gamma_i\right) - n^2 b_{\mathrm{max},i}^2 \quad \textbf{Eq. S11-5}$$

Hence, a plot of the first against the second term should be linear with slope:

$$\frac{d(b_{\mathrm{max}})^2}{d(\omega_{\mathrm{o}})^2} = \frac{\frac{d(b_{\mathrm{max}})^2}{d(n)^2}}{\frac{d(\omega_{\mathrm{o}})^2}{d(n)^2}} = \frac{1}{\gamma_i}\left(b_{\mathrm{max},i}\right)^2 \quad \textbf{Eq. S11-6}$$

The intercept is then $\frac{\beta}{r^2}$.

As n varies along the axial length of the CNT, this ratio remains invariant leading to a constant $\omega_{\mathrm{RBM,min}}$ observed from scan to scan, as observed.

We also considered the case of a general, nonlinear dependence of ω_{o} and b_{max} on n. In this case, expanded about zero:

$$\omega_{\mathrm{o}}(n) = \omega_{\mathrm{o}}(0) + \frac{d\omega_{\mathrm{o}}(0)}{dn} n + \ldots \quad \textbf{Eq. S11-7}$$

and

$$b_{\mathrm{max}}(n) = \frac{db_{\mathrm{max}}(0)}{dn} n + \ldots \quad \textbf{Eq. S11-8}$$

Hence, there is always a range of n where the proportionality of **Eq. S11-6** applies, as we observe in **Fig. 4a-d** of the main text.

Additional evidence that n appears to vary from spot to spot along the same CNT is provided by the FWHM, analyzed separately in the section below (**Supplementary Text 12:**) and in **Fig. 4** of the main text. Specifically, the FWHM, which should encompass both intrinsic and extrinsic (i.e., environmental) components, remains variable from spot to spot and in different

scans even in the high temperature ($T > T_{\max}$) limit, falsifying the notion that this limit corresponds to a pristine state of the CNT (i.e. thermally cleaned). **Eq. S11-4** anticipates this observation since the source of the damping is never removed of the observation site, only the strength of its coupling.

b) Evaluation of the intercept in terms of approximate *β*:

This section shows that the intercept values for all three CNTs analyzed in this way from **Eq. S11-5** above are within expected estimates. The origin of β comes from the case of a single CNT shell in the absence of external coupling to describe the RBM frequency[7]. The time-dependent, spatially uniform radial displacement $w(t)$ of a solitary, vibrating elastic shell is as follows:

$$\rho h \frac{\partial^2 w(t)}{\partial t^2} = \frac{-Eh}{(1-\upsilon^2)} \frac{w(t)}{r^2} \qquad \textbf{Eq. S11-9}$$

where ρ is mass density, h is shell thickness, E is Young's modulus, υ is Poisson's ratio, and $r = d/2$ is radius. A solution to **Eq. S11-9** of the form $w(t) = We^{i\omega_{\text{RBM}}t}$ gives the uncoupled RBM frequency as:

$$\omega_{\text{RBM,min}} = \beta = \frac{1}{r}\sqrt{\frac{Eh}{\rho h(1-\nu^2)}} \qquad \textbf{Eq. S11-10}$$

For **Supplementary Table 11-1,** the intercept can then provide an estimate of $Eh/(1-\nu^2)$. An approximate value of $\frac{Eh}{1-\nu^2} = 360\,\frac{\text{J}}{\text{m}^2}$ has been assumed in one particular study[1], along with the relation $\omega_{\text{RBM}}(\text{cm}^{-1}) = 231/d$ (nm) as well as $\rho = 2.27\ \text{g/cm}^3$ and $h = 0.34$ nm all for a single, environmentally uncoupled shell.

In this work, all three CNTs exhibit negative intercepts (l) of -12,282 cm^{-1}, -7,220 cm^{-1}, and -5,817 cm^{-1} for CNT F, G and X, respectively. **Eq. S11-5** anticipates this as the uncoupled frequency limit, $\frac{\beta}{r^2}$ assuming that each DWNT can be modeled using a Single Shell Approximation. Since all three have chirality assigned outer shells, the value of β can be estimated from the intercept value $\frac{\beta}{r^2}$ (**Supplementary Table 11-1**). As DWNT, we expect the interior shell to increase the frequencies of both shells. The results in **Supplementary Table 11-1** indicate that the intercept values, yielding an estimate of β, are within the correct frequency range.

Supplementary Table 11-1| Comparison of uncoupled frequencies predicted by Eq. (8) and mechanical models

DWNT	Chirality (i, j)	Outer diameter (d_0) [nm]	Slope (ν)	Intercept (l) [cm^{-1}]	$\omega_{RBM,limit}$ ($\sqrt{-\frac{l}{\nu}}$) [cm^{-1}]
CNT F	(19,3)@(22,11)	2.28	1.006	-12,282	110
CNT G	(29,1)@(35,6)	3.00	0.996	-7,220	85.1
CNT X	(28,5)@(28,11)	2.73	0.945	-5,817	78.5

12: RBM Peak Variance or Full Width at Half Maximum (FWHM)

The FWHM of the RBM peak also exhibits a temperature trajectory deceasing towards T_{max}, remaining invariant for $T > T_{\text{max}}$ at a minimum value that, unlike $\omega_{\text{RBM,min}}$, appears to vary from CNT location to location. This is evidence that the CNT remains environmentally coupled in the high temperature limit, consistent with the mechanism in the main text. The analysis below shows that the FWHM tracks and is scaled by the temperature dependent damping for the corresponding scan. The variability from spot to spot in the high temperature limit is consistent with the CNT in this limit still remaining in an environmentally coupled state that can be quantified by the relative value of the variance (i.e., variable graphitic carbon density, n).

a) The FWHM of the RBM peak tracks the RBM frequency for a given temperature scan

Supplementary Fig. 12-1 below shows 7 successive RBM frequency trajectories for CNT X at 2.5×10^{-5} bar with the corresponding FWHM at each temperature underneath each plot. The latter decreases towards $T_{\max}$ with increasing temperature, and after which remains invariant. The value of the FWHM generally decreases more than an order of magnitude, from 10 to 20 cm^{-1} at T_{o} to 1.0 to 4.0 cm^{-1} beyond $T_{\max}$.

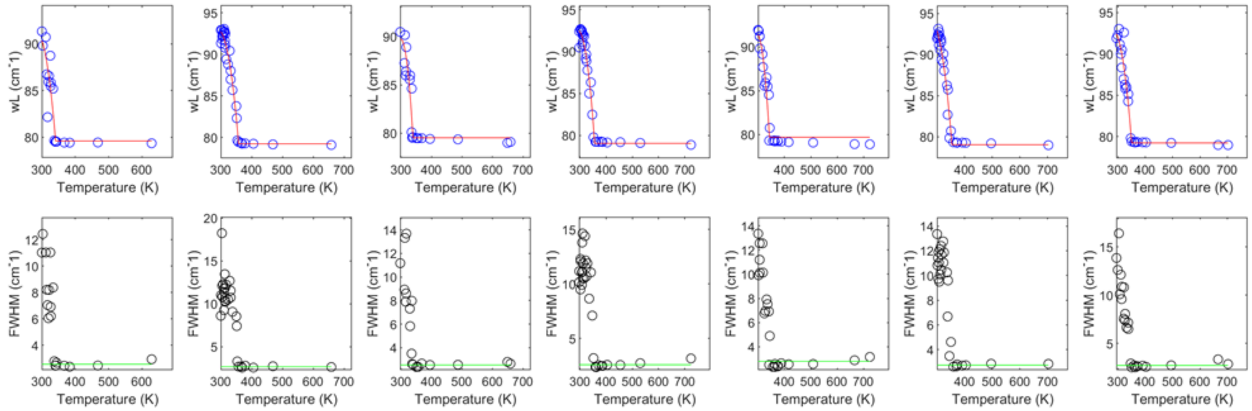

Supplementary Fig. 12-1| RBM frequency trajectories (top row) and corresponding FWHM trajectories (bottom row) for successive scans of CNT X at 2.5×10^{-5} bar.
The red curves for the former are the strain dependent coupling model while the green line for the latter is the average of FWHM values for T greater than the corresponding $T_{\max}$ value of the frequency. The value of the FWHM generally decreases more than an order of magnitude, from 10 to 20 cm^{-1} at T_{o} to 1.0 to 4.0 cm^{-1} beyond $T_{\max}$.

b) The minimum RBM variance across locations varies greater than between scans

While the minimum RBM frequency varies very little from scan to scan or spot to spot for the same CNT, the high temperature (minimum) variance is observed to vary considerably from spot to spot, indicating that a measurable portion of it remains environmentally coupled at high temperature. This is consistent with the presence of residual graphitic carbon that remains even at high temperature, as predicted by **Eq. S11-4** and its successful application to the data in **Fig. 4** of the main text. **Supplementary Table 12-1** to **Supplementary Table 12-3** and **Supplementary**

Fig. 12-2 compare the minimum RBM frequencies and FWHM variances for each scan of CNT X, G and F, respectively.

Supplementary Table 12-1| Comparison of minimum RBM frequencies and FWHM variances for CNT X.

"Matthias_CNT-X_211113_210803Bs01x1177_02_2e-2mbar.csv"

T_{max} (K)	b_{max} (cm^{-1})	Δb (cm^{-1})	ω_o (cm^{-1})	FWHM (cm^{-1})
330.40	50.83	30.38	93.86	4.21
331.80	46.66	42.48	91.94	4.48
320.40	47.50	59.24	92.22	4.27
329.50	46.97	42.78	92.00	4.56
321.60	45.14	47.54	91.17	4.45

"Matthias_CNT-X_211116_210803Bs01x1177_03_2e-2mbar_afterbakeout.csv"

T_{max} (K)	b_{max} (cm^{-1})	Δb (cm^{-1})	ω_o (cm^{-1})	FWHM (cm^{-1})
318.10	39.12	45.62	88.14	2.90
337.00	37.18	40.05	87.73	3.13
320.40	37.45	41.97	87.36	3.00
360.00	43.18	20.84	90.00	3.25
321.60	39.31	44.88	88.16	2.98
332.00	42.70	55.89	89.94	3.69

"Matthias_CNT-X_211220_210803Bs01x1177_08_3.4mbar.csv"

T_{max} (K)	b_{max} (cm^{-1})	Δb (cm^{-1})	ω_o (cm^{-1})	FWHM (cm^{-1})
335.30	48.80	52.80	92.93	2.63

"Matthias_CNT-X_211221_210803Bs01x1177_09_3.4mbar.csv"

T_{max} (K)	b_{max} (cm^{-1})	Δb (cm^{-1})	ω_o (cm^{-1})	FWHM (cm^{-1})
357.00	49.80	41.58	94.59	3.67
398.00	56.23	34.38	97.08	3.16

362.70	56.16	28.23	97.91	2.96
400.00	57.82	31.41	97.90	3.94
360.00	53.00	37.55	96.40	3.83
370.00	46.55	44.91	94.11	4.16

"Matthias_CNT-X_211222_210803Bs01x1177_10_2.5e-5mbar.csv"

T_{max} (K)	b_{max} (cm^{-1})	Δb (cm^{-1})	ω_o (cm^{-1})	FWHM (cm^{-1})
367.50	57.94	27.33	98.69	2.83
370.00	52.74	37.56	95.29	3.03
362.70	50.64	47.20	93.68	3.09

"Matthias_CNT-X_211223_210803Bs01x1177_11_2.5e-5mbar.csv"

T_{max} (K)	b_{max} (cm^{-1})	Δb (cm^{-1})	ω_o (cm^{-1})	FWHM (cm^{-1})
341.00	44.40	32.33	91.15	2.56
357.00	48.53	42.50	92.91	2.67
339.40	41.57	43.64	89.73	2.53
356.20	48.04	44.59	92.51	2.58
343.00	45.06	40.22	91.55	2.80
353.70	51.38	35.33	94.26	2.74
350.40	48.60	38.89	92.95	2.71

"Matthias_CNT-X_211228_210803Bs01x1177_12_1.9e-5mbar.csv"

T_{max} (K)	b_{max} (cm^{-1})	Δb (cm^{-1})	ω_o (cm^{-1})	FWHM (cm^{-1})
350.60	48.51	45.53	92.65	2.30
356.50	46.86	52.86	92.21	2.39
354.40	52.82	34.85	94.91	2.34
368.70	52.05	42.31	94.16	2.19
354.30	47.73	53.87	92.05	2.20

"Matthias_CNT-X_220105_210803Bs01x1177_13_1.6e-5mbar.csv"

T_{max} (K)	b_{max} (cm^{-1})	Δb (cm^{-1})	ω_o (cm^{-1})	FWHM (cm^{-1})
368.20	48.34	54.51	92.99	2.53
350.50	50.17	70.91	93.93	2.27
360.55	49.01	64.90	93.12	2.49
391.30	50.04	49.96	93.19	2.00
373.40	48.89	56.33	92.79	1.98
379.60	47.88	54.26	92.81	2.01

Supplementary Table 12-2| Comparison of minimum RBM frequencies and FWHM variances for CNT F.

"210519_210518As01x1132_LaserPowerScan_633nm_p7.4e-4mbar.csv"

T_{max} (K)	b_{max} (cm^{-1})	Δb (cm^{-1})	ω_o (cm^{-1})	FWHM (cm^{-1})
386.60	54.36	34.45	122.93	0.96
372.00	54.24	33.97	123.16	1.04
375.20	51.63	36.65	121.93	1.09

"210519_210518As02x1122_LaserPowerScan_633nm_p7.4e-4mbar.csv"

T_{max} (K)	b_{max} (cm^{-1})	Δb (cm^{-1})	ω_o (cm^{-1})	FWHM (cm^{-1})
406.40	51.96	41.55	121.97	1.04
395.60	47.42	55.61	120.70	1.13
412.40	64.52	28.58	127.93	1.21

"210519_210518As03x1117_LaserPowerScan_633nm_p7.4e-4mbar.csv"

T_{max} (K)	b_{max} (cm^{-1})	Δb (cm^{-1})	ω_o (cm^{-1})	FWHM (cm^{-1})
378.30	48.61	44.06	120.96	1.30
386.00	48.26	39.26	120.77	0.83
391.00	48.81	43.08	120.72	0.91

"210519_210518As04x1107_LaserPowerScan_633nm_p7.4e-4mbar.csv"

T_{max} (K)	b_{max} (cm^{-1})	Δb (cm^{-1})	ω_o (cm^{-1})	FWHM (cm^{-1})
378.30	57.79	32.35	124.66	1.12
386.00	63.70	25.12	127.33	1.18
391.00	59.86	29.04	125.43	1.03

"210521_210518As01x1132_LaserPowerScan_633nm_p2.5e0mbar.csv"

T_{max} (K)	b_{max} (cm^{-1})	Δb (cm^{-1})	ω_o (cm^{-1})	FWHM (cm^{-1})
567.00	41.56	36.32	117.87	2.61
357.00	47.15	31.86	119.88	1.72

351.00	44.63	48.58	118.89	1.65

"210521_210518As02x1122_LaserPowerScan_633nm_p2.5e0mbar.csv"

T_{max} (K)	b_{max} (cm^{-1})	Δb (cm^{-1})	ω_o (cm^{-1})	FWHM (cm^{-1})
368.00	49.30	51.34	120.71	1.27
357.00	50.67	63.42	121.25	1.12
351.00	59.12	24.51	125.01	1.15

"210521_210518As03x1117_LaserPowerScan_633nm_p2.5e0mbar.csv"

T_{max} (K)	b_{max} (cm^{-1})	Δb (cm^{-1})	ω_o (cm^{-1})	FWHM (cm^{-1})
357.00	59.32	33.32	125.11	1.02
351.00	50.64	46.02	121.23	1.04

"210521_210518As04x1107_LaserPowerScan_633nm_p2.5e0mbar.csv"

T_{max} (K)	b_{max} (cm^{-1})	Δb (cm^{-1})	ω_o (cm^{-1})	FWHM (cm^{-1})
357.00	59.45	35.70	125.15	1.11
351.00	51.36	62.91	121.56	1.09

"210525_210518As02x1122_LaserPowerScan_633nm_p1.2e-3mbar.csv"

T_{max} (K)	b_{max} (cm^{-1})	Δb (cm^{-1})	ω_o (cm^{-1})	FWHM (cm^{-1})
617.70	42.70	33.29	118.10	1.37
381.70	54.44	35.48	123.06	1.10
376.70	43.57	52.55	118.68	1.33

"210525_210518As03x1117_LaserPowerScan_633nm_p1.2e-3mbar.csv"

T_{max} (K)	b_{max} (cm^{-1})	Δb (cm^{-1})	ω_o (cm^{-1})	FWHM (cm^{-1})
565.40	43.29	36.28	118.69	1.23
360.30	54.88	33.69	123.37	1.23

352.30	50.70	37.60	121.60	1.32

"210525_210518As04x1107_LaserPowerScan_633nm_p1.2e-3mbar.csv"

T_{max} (K)	b_{max} (cm^{-1})	Δb (cm^{-1})	ω_o (cm^{-1})	FWHM (cm^{-1})
565.40	42.74	37.60	118.29	1.41
350.00	51.06	41.43	121.72	1.21
352.30	49.78	49.36	121.49	1.07
355.00	52.20	34.86	122.44	1.15

Supplementary Table 12-3| Comparison of minimum RBM frequencies and FWHM variances for CNT G.

"210628_210621Bs02x0168_633nm_p1.2e-3mbar.csv"

T_{max} (K)	b_{max} (cm^{-1})	Δb (cm^{-1})	ω_o (cm^{-1})	FWHM (cm^{-1})
701.10	41.72	33.83	94.40	1.10
423.10	46.51	43.86	97.01	1.01
423.30	51.69	32.05	99.70	1.05
405.00	48.79	41.98	98.55	1.41
400.00	46.21	52.59	97.17	0.88

"210628_210621Bs03x0181_633nm_p1.2e-3mbar.csv"

T_{max} (K)	b_{max} (cm^{-1})	Δb (cm^{-1})	ω_o (cm^{-1})	FWHM (cm^{-1})
571.60	43.85	45.41	96.38	0.93
415.00	51.01	35.56	99.49	1.05
407.00	47.26	46.25	97.99	1.14
399.00	45.62	47.40	97.24	1.10
411.00	47.33	50.92	97.74	1.07
411.00	46.24	48.73	97.57	2.53
411.00	45.59	52.76	96.72	1.18

"210628_210621Bs04x0188_633nm_p1.2e-3mbar.csv"

T_{max} (K)	b_{max} (cm^{-1})	Δb (cm^{-1})	ω_o (cm^{-1})	FWHM (cm^{-1})
780.10	52.61	22.92	99.50	1.21
391.30	46.85	50.72	96.60	1.53
388.30	46.63	50.16	96.77	1.37
383.73	46.86	53.63	96.73	1.24
387.00	45.95	48.38	96.53	1.56

"210628_210621Bs05x0196_633nm_p1.2e-3mbar.csv"

T_{max} (K)	b_{max} (cm^{-1})	Δb (cm^{-1})	ω_o (cm^{-1})	FWHM (cm^{-1})
550.00*	243.15*	1.92*	259.45*	9.63*

400.00*	38.03*	34.53*	95.78*	6.41*
407.00	49.07	37.12	97.21	1.15
398.00	47.48	48.28	96.56	1.22
390.00	47.00	53.10	96.35	1.13

*Note: Scans after the double humps (rare trajectories in the **Supplementary Text 3: d**) are scattered.

c) Thermal annealing for 12 hrs systematically decreases the RBM variance

After the 12-hour annealing experiment at 2.2×10^{-5} bar at 100 ºC, the average high temperature FWHM.

Supplementary Table 12-4| Comparison of minimum RBM frequencies and FWHM variances for CNT X before and after bakeout at 100 ºC for 12 hrs.

"Matthias_CNT-X_211113_210803Bs01x1177_02_2e-2mbar.csv"

T_{max} (K)	b_{max} (cm^{-1})	Δb (cm^{-1})	ω_o (cm^{-1})	FWHM (cm^{-1})
330.40	50.83	30.38	93.86	4.21
331.80	46.66	42.48	91.94	4.48
320.40	47.50	59.24	92.22	4.27
329.50	46.97	42.78	92.00	4.56
321.60	45.14	47.54	91.17	4.45

Mean FWHM for 5 scans = 4.40 (cm^{-1})

Standard deviation = 0.15 (cm^{-1})

"Matthias_CNT-X_211116_210803Bs01x1177_03_2e-2mbar_afterbakeout.csv"

T_{max} (K)	b_{max} (cm^{-1})	Δb (cm^{-1})	ω_o (cm^{-1})	FWHM (cm^{-1})

318.10	39.12	45.62	88.14	2.90
337.00	37.18	40.05	87.73	3.13
320.40	37.45	41.97	87.36	3.00
360.00	43.18	20.84	90.00	3.25
321.60	39.31	44.88	88.16	2.98
332.00	42.70	55.89	89.94	3.69

Mean FWHM for 5 scans = 3.16 (cm^{-1})

Standard deviation = 0.29 (cm^{-1})

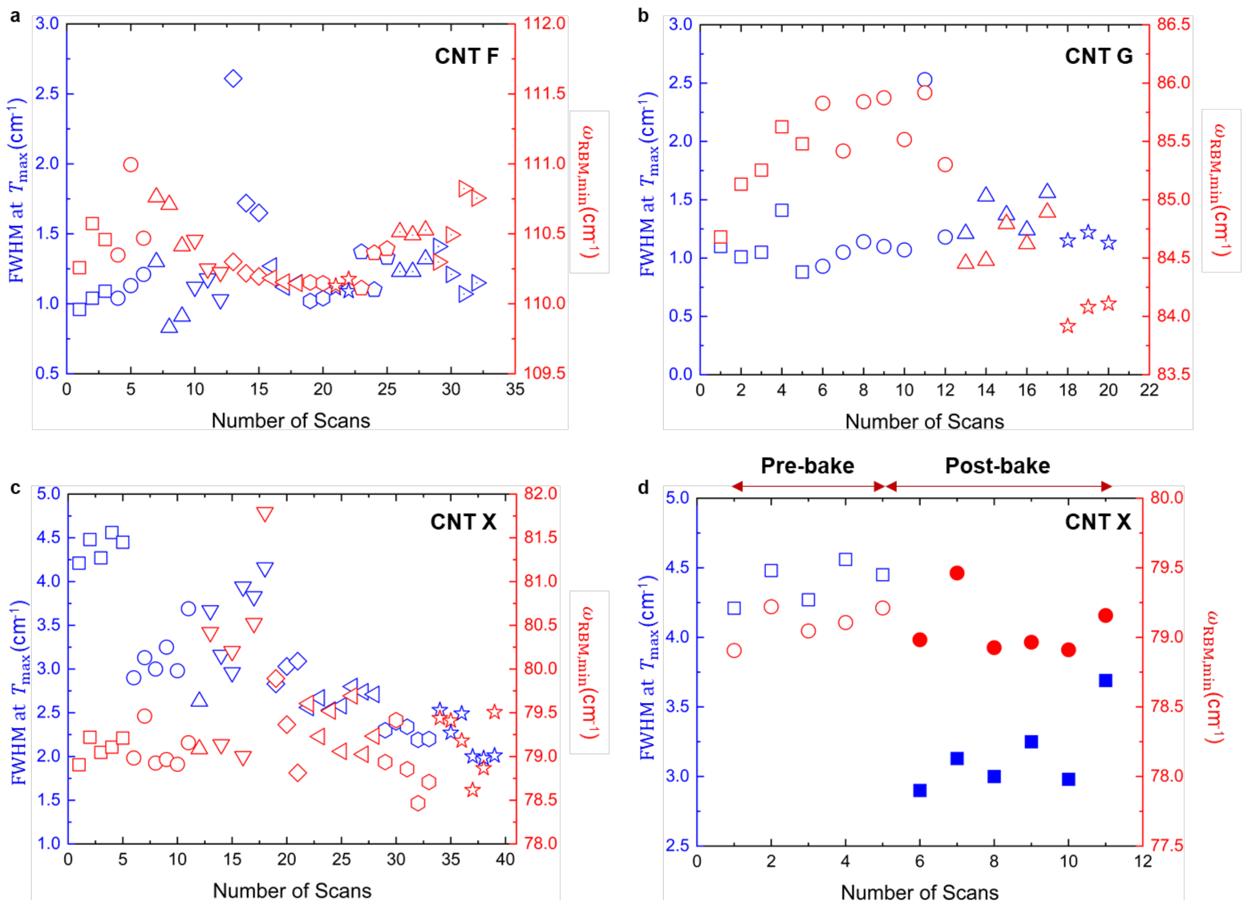

Supplementary Fig. 12-2| Comparison of FWHM ($T > T_{max}$) and $\omega_{RBM,min}$ variance between locations and vacuum conditions.

FWHM and $\omega_{RBM,min}$ of (**a**) CNT F, (**b**) CNT G, and (**c**) CNT X. FWHM is not constant at the T_{max} between scans, which indicate that the CNT is not pristine at high temperature. The different symbols in **a-c** represent the Raman scans at different spots of the same CNT and vacuum condition. **d**, FWHM and $\omega_{RBM,min}$ of CNT X change upon thermally annealing. Scans 1-5 are pre-bake (as-grown) CNT X. After 12 hr annealing of CNT X (Scans 6-11),

$\omega_{RBM,min}$ remains nearly invariant and FWHM becomes narrow. The result also indicates the decrease in the inhomogeneity of the system and the number of carbonaceous tethers.

d) The RBM variance tracks the temperature dependent change in damping, $b[T]$

As shown in the **Supplementary Text 12: a**, the RBM FWHM appears to track the RBM frequency with temperature, showing the same cusp at $T_{\max}$, and limiting behavior at $T > T_{\max}$. Approximating the Lorentzian RBM peak as a Gaussian, the square of the FWHM is proportional to the peak variance, which should be additive, including an intrinsic component σ_i^2 (at T_o) and a component associated with the same graphitic carbon that is the source of the damping, generating the temperature dependent variance $\sigma_b^2[T]$.

The sum can be expressed as:

$$\left(\frac{RBM\ FWHM}{2\sqrt{2\ ln(2)}}\right)^2 = \sigma_T^2 = \sigma_i^2 + \sigma_b^2[T] \qquad \textbf{Eq. S12-1}$$

Note that we do not observe any evidence that the intrinsic variance itself is temperature dependent over this range. This is confirmed by the limiting minimum in FWHM seen in **Supplementary Fig. 12-1** for $T > T_{\max}$, spanning more than 200 K in temperature.

A mechanism that attributes the temperature dependent variance to the carbon generating the damping response asserts the following proportionality:

$$\sigma_b^2[T] \propto b^2[T] \qquad \textbf{Eq. S12-2}$$

The limiting variance then becomes:

$$\sigma_b^2[T > T_{\max}] \propto b_{\max}^2 \qquad \textbf{Eq. S12-3}$$

This suggests that $b_{\max}$ is a natural normalization of the RBM variance. The intrinsic variance can be approximated as the minimum observed such that $\sigma_i \sim min(\sigma_T)$. It is otherwise difficult to generate and observe CNTs confirmed to be free of all carbon impurities.

These proportionalities suggest the following scaling for the RBM variances for a series of scans at a CNT spot.

$$\frac{\sigma_T^2 - \sigma_i^2}{\sigma_i^2} \sim \left(\frac{b[T]}{b_{\mathrm{max}}}\right)^2 \qquad \textbf{Eq. S12-4}$$

Supplementary Fig. 12-3 generates this comparison of variances for four data sets for CNT X as the compilation of all thermal trajectories in each series. Note that these plots analyze all of the FWHM data, not just $T > T_{\mathrm{max}}$ limit, with considerable variability observed for the $T < T_{\mathrm{max}}$ data points, similar to the scatter in Δb for each scan. We see that that all of the high temperature variances collapse to a single cluster of points around $\frac{b}{b_{\mathrm{max}}}$. This confirms that the variance for each trajectory tracks the same mechanism changing the damping. Note that the value of b_{max} varies between locations and scans, consistent with variable numbers of tethers being observed. However, while $\omega_{\mathrm{RBM,min}}$ remains invariant due to the compensatory influences of spring and damping constants, the limiting FWHM ($FWHM_o$) varies significantly from location to location but appears to scale adequately with the magnitude of the maximum damping (b_{max}), supporting a mechanism whereby its origin is the same graphitic carbon. Since b_{max} is proportional to the surface density of carbon impurities in the spot (n), the damping mechanism described in the main text appears to account for the variation of the high temperature FWHM from spot to spot, as each location experiences variable inhomogeneous broadening.

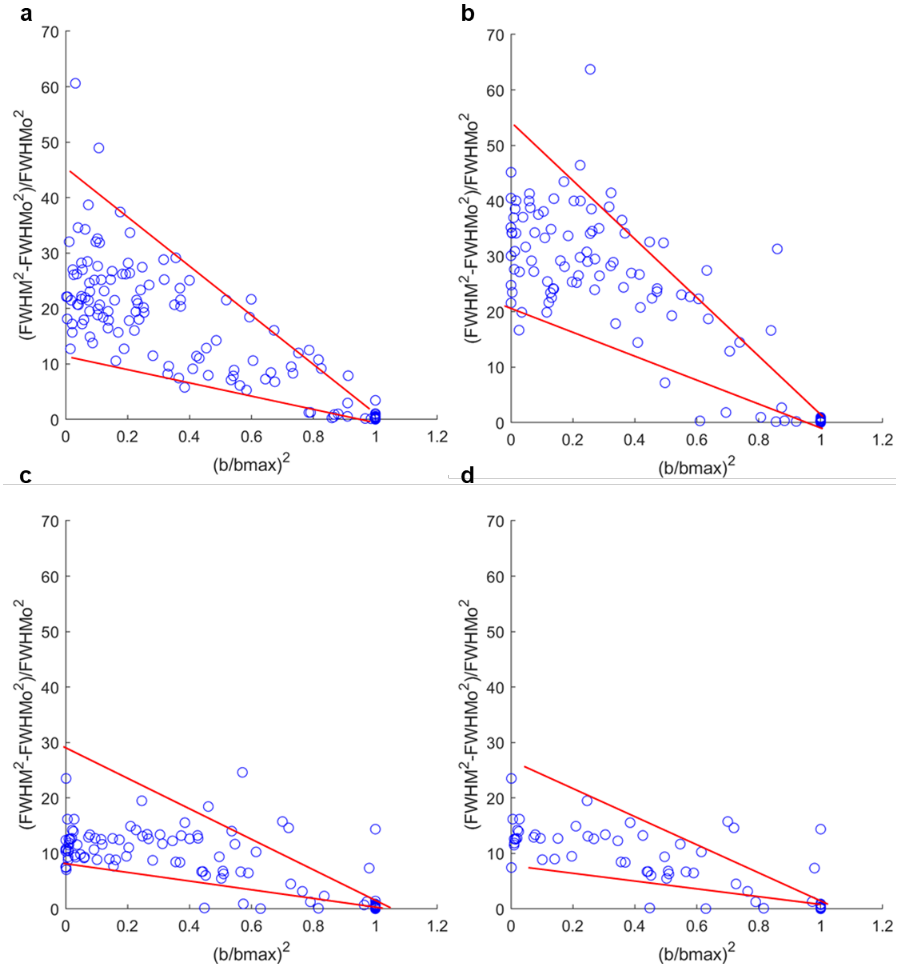

Supplementary Fig. 12-3 | Selected scaled variances for the whole temperature scan versus scaled temperature-dependent damping constant of CNT X.
The analyses are from the data set in **a**, (*Matthias_CNT-X_211222_210803Bs01x1177_10_2.5e-5mbar.csv)*; **b**, (*Matthias_CNT-X_211228_210803Bs01x1177_12_1.9e-5mbar.csv*); **c**, (*Matthias_CNT-X_211113_210803Bs01x1177_02_2e-2mbar.csv*); **d**, (*Matthias_CNT-X_211116_210803Bs01x1177_03_2e-2mbar_afterbakeout.csv*). In all four plots, the variances of FWHM are larger when the system is in the low damping state. The variances converge to the magnitude of b_{max}, suggesting the coupling mechanism is from same source (graphitic carbon) on the CNT surface. The red lines in these plots are used as visual guides for interpretation.

Note that in the above, the points at $T > T_{\max}$ for each scan collapse to a single point at $\frac{b}{b_{\max}} = 1$, indicating that the FWHM scales with $b_{\max}$ (and therefore n) in this limit, in support of the strain dependent mechanism.

13: Comparison with Prior Literature Measurements of the RBM

In this section we consider whether previous studies of SWNT and DWNT RBM frequencies have produced values distinct from this work. Past measurements, nominally on pristine CNTs free from environmental coupling, specifically the carbon masses studied in this work, have produced RBM frequencies for specific DWNT for comparison to this work. **Eq. S11-4** above predicts that independent of the surface density of adsorbed carbon, the observed frequency of the RBM remains invariant because the average spring constant increase is offset from a damping decrease. If studies of presumably clean CNTs hypothetically produced frequencies distinct from those in this work, this would be a contradiction to the **Supplementary Text 11:**. To compare DWNTs across the literature, we needed to extract an estimate of the carbon-carbon coupling spring constant between shells (see below). However, the findings of this section indicate that our minimum RBM frequencies ($\omega_{\mathrm{RBM,min}}$), extracted from the $T > T_{\mathrm{max}}$ region of the trajectory, are in general agreement with previous literature, providing a validation of **Eq. S11-4**.

a) Comparison with Liu *et al.*[8]

Liu *et al.*[8] measured the inner and outer shell RBM frequencies of a series of electron-diffracted DWNTs under conditions very similar to those in this work. The authors used a vacuum stage and suspended DWNTs across a Raman microscopy window in a reasonable attempt to remove all environmental coupling. These RBM frequencies were analyzed using an elastic shell model to extract the shell-shell spring constant, γ_{CC}, assuming environmentally uncoupled oscillators. If we add the $\omega_{\mathrm{RBM,min}}$ frequencies measured for CNT F, G, X in this work for both the inner and outer shells, to this same analysis and extract γ_{CC} values using the same undamped

($n = 0$), force balance, these are found to fall closely within the trend of Liu *et al.*[8] The system of Liu *et al.*[8] is not reported to be under tension, and so the mechanism of this work predicts that the authors would observe only an invariant $\omega_{\mathrm{RBM,min}}$ at T_{o} or with a change in temperature. However, the agreement between the γ_{CC} values indicates that Liu *et al.*[8] DWNT RBM frequencies agree with those of this work.

b) Scaling γ_{CC} values for a series of DWNT

As an aid in organizing the scaling of shell-shell coupling parameter, γ_{CC}, we note that the Lennard-Jones potential is insufficient for describing its dependence on dr, for both the Lui *et al.*[8] data and that of this work. Instead, both are well described by a Buckingham potential (green line), which empirically is:

$$\gamma_{j,j+1}(\Delta r)\left[\frac{\mathrm{GPa}}{\mathrm{nm}}\right] = 5.443 \cdot 10^{6} \cdot \exp\left(-\frac{\Delta r}{0.03895}\right) \cdot (\Delta r - 0.03895) - \frac{0.215}{\Delta r^{6}} \qquad \textbf{Eq. S13-1}$$

where $\Delta r = r_2 - r_1$ is in nanometer (nm). We use this expression to estimate γ_{CC} in DWNT as $\gamma_{\mathrm{CC}} = 95\ \mathrm{GPa/nm}$ at an example inter-tube spacing of $\Delta r = 0.35$ nm.

Inter-shell spring constants can be calculated using the two-shell force:

$$\begin{bmatrix} -\rho h\omega^2 + \frac{Eh}{1-\nu^2}\frac{1}{r_1^2} + \gamma_{\mathrm{CC}} & -\gamma_{\mathrm{CC}} \\ -\gamma_{\mathrm{CC}}\frac{r_1}{r_2} & -\rho h\omega^2 + \frac{Eh}{1-\nu^2}\frac{1}{r_2^2} + \gamma_{\mathrm{CC}}\frac{r_1}{r_2} \end{bmatrix}\begin{bmatrix} W_1 \\ W_2 \end{bmatrix} = \begin{bmatrix} 0 \\ 0 \end{bmatrix} \qquad \textbf{Eq. S13-2}$$

Eq. S13-2 gives two RBMs as a high-frequency, inner shell mode ($\omega_{\mathrm{RBM,H}}$) characterized by counter-phase oscillation of both walls for which $\operatorname{sgn} W_1 = -\operatorname{sgn} W_2$, and a low frequency, outer shell mode ($\omega_{\mathrm{RBM,L}}$) characterized by in-phase oscillation of both walls for which $\operatorname{sgn} W_1 = \operatorname{sgn} W_2$. For shell coupling calculations, we utilized values of $Eh/(1-\nu^2) = 353\ \mathrm{J/m^2}$, $\rho = 2.27\ \mathrm{g/cm^3}$, and $h = 0.34$ nm, yielding a value of $\gamma_{\mathrm{CC}} = 95\ \mathrm{GPa/nm}$. The latter two values are

determined by considering the tube-tube potential as a function of the interwall spacing, as discussed below.

While the vdW interaction between carbon shells can be described by a Lennard-Jones potential, the exchange-repulsion component of the binding energy is better described using an exponential, as employed in a Buckingham potential (**Eq. S13-1**). On the other hand, the interaction described by a Lennard-Jones potential is as follows:[9,10]

$$U(\delta r) = 2\pi\rho_c^2\epsilon\sigma^2\left[0.4\left(\frac{\sigma}{\delta r}\right)^{10} - \left(\frac{\sigma}{\delta r}\right)^{4}\right] \qquad \textbf{Eq. S13-3}$$

where δr is the radial intershell separation, $\sigma = 0.34$ nm,[9] $\rho_\mathrm{c} = 4/(3\sqrt{3}a_0^2)$ is the areal density of carbon atoms in a graphene sheet with $a_0 = 0.142$ nm the nearest-neighbor carbon-carbon distance in graphene, and ϵ is the well depth of the corresponding pairwise Lennard-Jones potential. A plot of the continuum potential is shown in **Supplementary Fig. 13-1a**. The vdW interaction coefficient between carbon shells j and $j+1$ can be estimated from the potential as:

$$\gamma_{j,j+1} = \left.\frac{d^2U}{d(\delta r)^2}\right|_{\delta r=\Delta r_{j,j+1}} = 8\pi\rho_c^2\epsilon\left(11\frac{\sigma^{12}}{\Delta r_{j,j+1}^{12}} - 5\frac{\sigma^6}{\Delta r_{j,j+1}^{6}}\right) \qquad \textbf{Eq. S13-4}$$

As can be seen in **Supplementary Fig. 13-1b**, $\gamma_{j,j+1}$ strongly depends on the radial distance δr between neighboring carbon shells. Smaller corrections due to curvature effects and chiral angle mismatch between neighboring tubes are not considered in this analysis[11-13]. In this work, we were able to assign the radii of three free-standing DWNTs by electron diffraction and observe radial breathing-like modes (RBLMs). A fit of these RBLM frequencies with the solution of **Eq. S13-2** yielded $\beta = Eh/(1-\nu^2) = 353 \pm 4\ \mathrm{J/m^2}$ as well as the γ_CC values shown as green dots in **Supplementary Fig. 13-1b**. Note that $\beta = 353 \pm 4\ \mathrm{J/m^2}$ corresponds to a slope of 227 ± 1 nm cm^{-1} in the $\omega_\mathrm{RBM} = \frac{\mathrm{slope}}{d\ [\mathrm{nm}]}$ relation commonly used for SWNT, confirming a previous analysis.[14] The best fit of our γ_CC values by **Eq. S13-4** with $\sigma = 0.34$ nm is shown as a black line

in **Supplementary Fig. 13-1b**. While this fit yields $\epsilon = 3.16$ meV, which is comparable to the graphene-graphene value $\epsilon = 2.39$ meV reported in Ref. [9], we obtain only poor agreement with the experimental data. Better agreement with the experiment can only be obtained for both unphysically low values of ϵ and unphysically large values of σ, i.e., the Lennard-Jones potential fails to describe the Δr dependence of the intershell vdW coupling. Instead, we turn to a pairwise Buckingham potential of the form $U(r) = A \exp\left(-\frac{r}{s}\right) - \frac{B}{r^6}$, which yields the continuum potential of the form:

$$U(\delta r) = 2\pi\rho_c^2 \left[A \exp\left(-\frac{\delta r}{s}\right) s(\delta r + s) - \frac{1}{4}\frac{B}{\delta r^4}\right] \qquad \textbf{Eq. S13-5}$$

and a vdW interaction coefficient between carbon shells j and $j+1$ as follows:

$$\gamma_{j,j+1} = 2\pi\rho_c^2 \left(\frac{A\mathrm{e}^{-\frac{\delta r}{s}}(\delta r - s)}{s} - \frac{5B}{\delta r^6}\right) \qquad \textbf{Eq. S13-6}$$

Requiring a value of s such that a potential well of similar magnitude as for the case of the Lennard-Jones potential is obtained, we fit our experimental data in **Supplementary Fig. 13-1b** with **Eq. S13-6** and obtain $A = 1.45 \cdot 10^5$ meV, $B = 2.93 \cdot 10^4$ meV $\cdot$ Å^6, and $s = 0.039$ nm. These values compare with graphene-graphene Buckingham parameters reported by Liu *et al.*[15]: $A = 3.31 \cdot 10^5$ meV, $B = 9.49 \cdot 10^4$ meV $\cdot$ Å^6, and $s = 0.038$ nm. The discrepancy between experiment and estimates based on the Lennard-Jones potential is corroborated by data from Liu *et al.*[8] collected on DWNTs in presumably adsorbate-free state, which we have included as grey data points in **Supplementary Fig. 13-1b**. These data agree well with our fit based on the Buckingham potential (green line), which empirically can be stated as

$$\gamma_{j,j+1}(\Delta r)\left[\frac{\mathrm{GPa}}{\mathrm{nm}}\right] = 5.443 \cdot 10^6 \cdot \exp\left(-\frac{\Delta r}{0.03895}\right) \cdot (\Delta r - 0.03895) - \frac{0.215}{\Delta r^6} \qquad \textbf{Eq. S13-7}$$

where $\Delta r = r_2 - r_1$ is in nm. We use this expression to estimate γ_{CC} in DWNT as $\gamma_{CC} = 95\ \mathrm{GPa/nm}$ at an example inter-tube spacing of $\Delta r = 0.35\ \mathrm{nm}$.

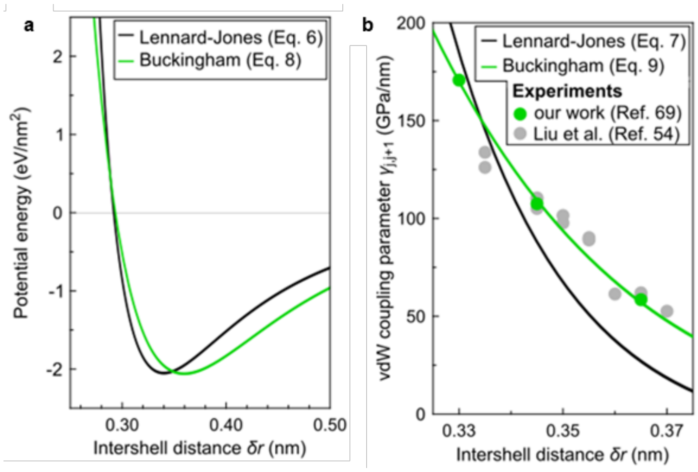

Supplementary Fig. 13-1| Comparison of experimental data with Lennard-Jones and Buckingham models. **a**, Continuum vdW potentials between carbon walls according to **Eq. S13-3**and **Eq. S13-5**, using parameters obtained for the fits of $\gamma_{j,j+1}(\delta r)$ in **b**. **b**, vdW coupling parameter between neighboring carbon shells as a function of intershell distance. Shown are best fits to our experimental data using **Eq. S13-4**and **Eq. S13-6** as described in the text.

The data from Liu *et al.*[8] consist of RBM frequencies of a series of ED-assigned DWNTs in vacuum, extracting shell-shell coupling constants under conditions similar to those in this work. The points displayed in **Supplementary Fig. 13-1** above disperse along the Buckingham potential curve with reasonable fidelity. If we take values for $\omega_{\mathrm{RBM,min}}$, the limiting frequency in the highly damped limit measured in this work for CNTs F, G, and X and use them in the **Eq. S13-2** force balance above, we obtain the green points above which fall closely within the scaling range of Liu *et al.*[8] This indicates that the DWNTs measured in Liu *et al.*[8] are in agreement with the RBM low frequencies for CNT F, G and X in this work. This agreement across two studies provides a validation of **Eq. S11-4** in this work.

14: RBM Trajectories Change after FIB Cutting and Interior Water Filling

The suspended CNT platform was fabricated and synthesized on commercially available porous Si_3N_4 membranes (Ted Pella, Inc) using the same CVD procedure mentioned above. The as-synthesized CNTs were focused Ga^+ ion beam (FIB) cutting to open both ends of CNTs (28 pA, 30 kV, 100 passes, 2 µs dwell time, 4.27 nm pitch) (**Supplementary Fig. 14-1a**)[16]. Subsequently, the FIB-cut samples were exposed to 100% DI water vapor by placing them in a high humidity chamber. To ensure airtight conditions, both ends of the samples were sealed using Torr seal glues (**Supplementary Fig. 14-1b**). The sealed samples were left to dry overnight prior to conducting isobar experiments of CNTs under vacuum (10^{-6} bar) (**Supplementary Fig. 14-1c**). Samples prepared in this way were examined by TEM mode at MIT Nano using Titan Themis Z G3 Cs-Corrected S/TEM for TEM video acquisitions (**Supplementary Fig. 14-1d-f, and Supplementary Movie 2)**, and Raman spectroscopic RBM trajectory measurements at MIT for comparison[17].

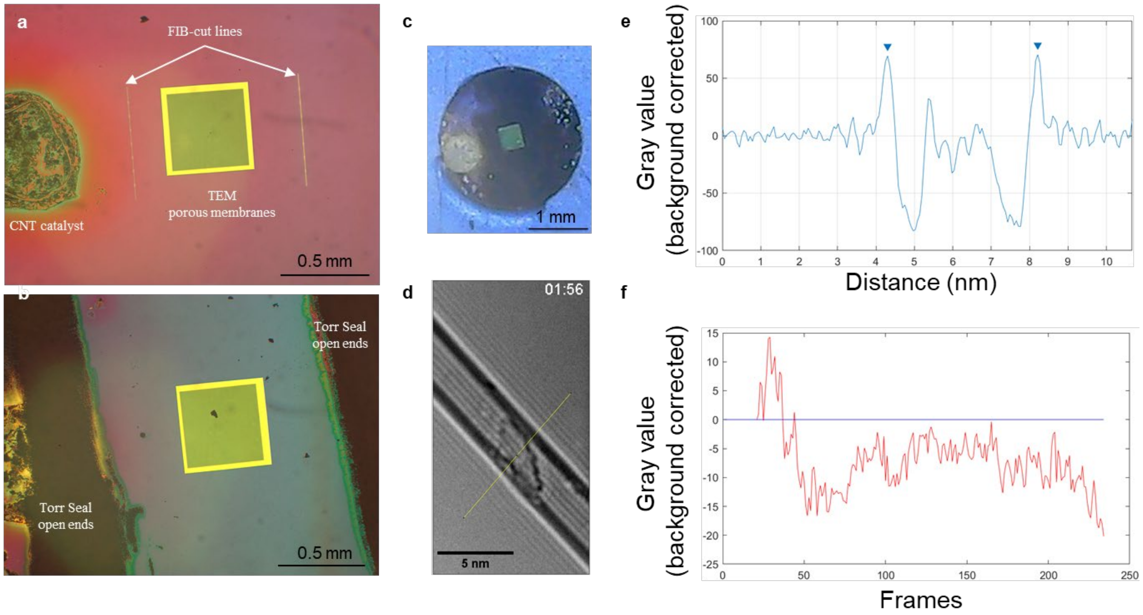

Supplementary Fig. 14-1| Interior water filled CNT sample fabrication and TEM imaging.

a, A microscopic image of FIB-cut opened CNT samples. Left: a drop-casted CNT catalyst; Squared window: 0.5 mm x 0.5 mm TEM window with 200 nm pores on a Si_3N_4 membrane; Both ends: FIB-cut lines. **b**, A microscopic image of a Torr-sealed CNT sample. Following the CNT sample exposure to water vapor, Torr seal glues were applied outside of the TEM window to seal the both ends of CNTs. **c**, A picture of the interior water vapor-filled CNT sample. **d**, A still frame of a TEM video (**Supplementary Movie 2**). **e**, The background-corrected plot profile of the yellow line across the interior entity shown in **e**. **f**. The time series of the cross-sectional plot profile shows that the internal entity appears darker than the background and dynamically forms over time. This observation suggests that the CNT is filled with fluid after the water filling procedure and may create water-carbon oxidation products from the electron beam. The blue line at the value of 0 in the plot is used as visual guide. The exposure time is 0.5 s per frame.

a) TEM Imaging of Interior Water is Statistically Rare

It was found, conversely, that imaging of interior water using TEM was relatively rare, even on samples known to have a significant yield of interior water from humidity filling. The consensus among several imaging experts (J. Cumings, A. Majundar, J. Hatchal) is that an interior water phase lacks the TEM contrast necessary to distinguish it from an empty CNT. When an interior vacuum-fluid interface is visible in a CNT segment, as in **Fig. 5d** of the main text and **Supplementary Fig. 14-1d**, a positive assignment of an imaged fluid phase can be made.

b) RBM Trajectories for Interior Filled CNTs Exhibit a Positive Second Derivative at High Temperature

Samples subjected to the process of interior fluid filling exhibit a distinct change in RBM trajectory compared to the behavior attributed to strain dependent coupling in the main text. The example below shows 7 thermal scans of the RBM of a suspended, filled and Torr-sealed CNT under conditions of interior fluid filling (**Supplementary Fig. 14-2** and **Supplementary Fig. 14-3**). The trajectories for this CNT and three others measured using Raman spectroscopy as outlined in the main text exhibit positive second derivatives at high temperature. The successful filling rate is about 4/31 CNTs from 8 TEM chips proceeded through water-filling procedure. This transition to a positive second derivative is anticipated by the mathematical proof in **Supplementary Text 6: a** above. We also observed two CNTs showed negligible temperature

change on temperature scanning. None of the trajectories exhibit the characteristic $T_{\max}$ cusp developed in the main text, or the hyperbolic, concave down trajectory of the strain dependent coupling mechanism. This is consistent with a trajectory now dominated by the temperature-dependent internal fluid adsorption and desorption within the CNT.

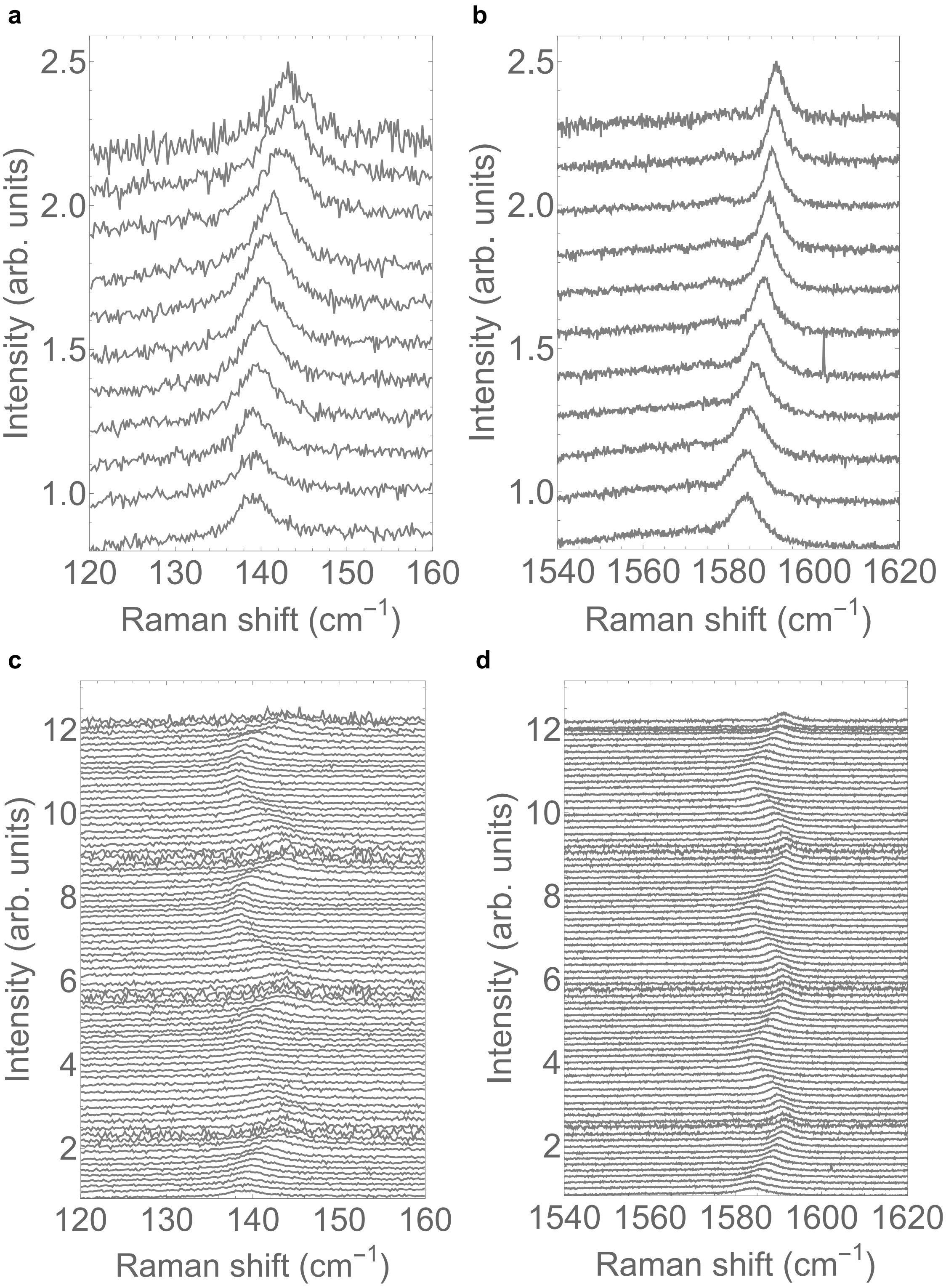

Supplementary Fig. 14-2| 78 Raman spectra of CNT (Fig. 5e) scanned with a pressure of 4e-2mbar
Raman spectra of the first local laser power cycle scans (11 Raman spectra): **a**, RBM region and **b**, G band region. The full Raman scans of **CNT (Fig. 5e)** in this experiment: **c**, RBM region and **d**, G band region.

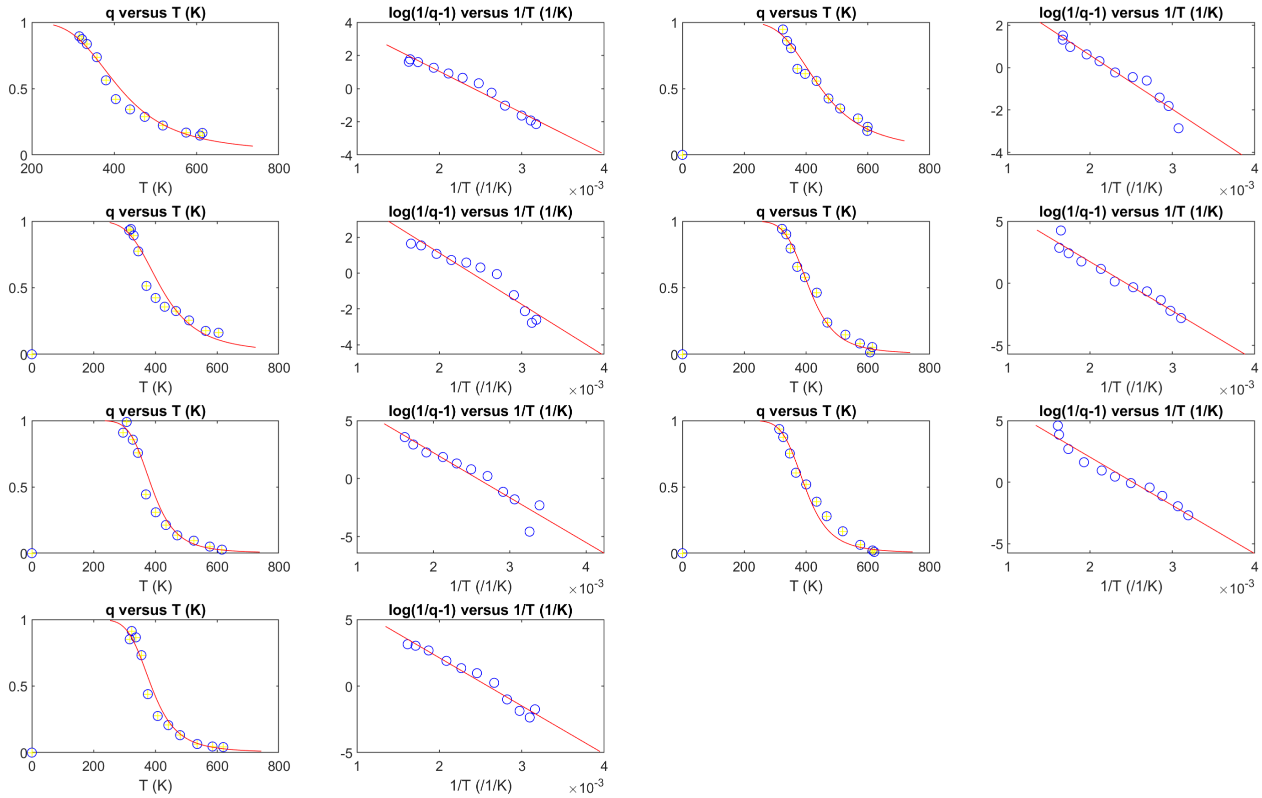

Supplementary Fig. 14-3| Interior filled isobars of water vapor-filled CNTs.
Seven laser heating cycles were conducted on the water vapor-filled, partially suspended, Torr-sealed CNTs. The RBM of the interior wall displays a reversible thermal trajectory and positive second derivative. The results were fitted to the Langmuir isobar as shown in the red curves/ lines.

The positive second derivative means that the trajectory can be described by the Langmuir isobar in **Eq. S6-4**. To quantitatively analyze fluid isobars, we convert measured RBM frequencies into fractional coverage (q) from the **Eq. S11-9**[18,19]

For a single-walled CNT, the vapor state RBM frequency (ω_V) is

$$\omega_{\mathrm{V}}^2 = \frac{1}{r^2} \cdot \frac{Eh}{\rho h(1-\nu^2)} \qquad \textbf{Eq. S14-1}$$

where r is the SWCNT radius, E is the Young modulus, h is the SWCNT wall thickness, ρ is the mass density, and ν is the Poisson ratio.

The liquid state RBM frequency (ω_{L}) is defined as

$$\omega_{\mathrm{L}}^2 = \omega_{\mathrm{V}}^2 + \frac{\gamma}{\rho h(1-\nu^2)} \qquad \textbf{Eq. S14-2}$$

where γ is an area-normalized spring constant characterizing the van der Waals coupling between the carbon shell and the first shell of adsorbed molecules. γ is related to the second derivative of the potential between the two shells.

We define the area-normalized spring constant at fractional coverage q as $q\gamma$, **Eq. S14-2** becomes:

$$\omega_{\mathrm{RBM}}^2 = \omega_{\mathrm{V}}^2 + \frac{q\gamma}{\rho h(1-\nu^2)} \qquad \textbf{Eq. S14-3}$$

where ω_{RBM} is the RBM frequency at fractional coverage q.

Combining Langmuir isobar in **Eq. S6-4** and **Eq. S14-3,** it follows the **Eq. (10)** of the main text:

$$q[T] = \frac{\omega_{\mathrm{RBM}}^2 - \omega_{\mathrm{V}}^2}{\omega_{\mathrm{L}}^2 - \omega_{\mathrm{V}}^2} = \frac{\mathrm{e}^{\frac{\Delta H}{RT}} P K_{\mathrm{o}}}{1 + \mathrm{e}^{\frac{\Delta H}{RT}} P K_{\mathrm{o}}} \qquad \textbf{Eq. S14-4}$$

where $q[T]$ is the surface coverage, ω_{V} (at which $q = 0$) is the lowest observed RBM frequency, ω_{L} (at which $q = 1$) is the highest observed RBM frequency, K_{o} is equilibrium constant, ΔH is the heat of adsorption, and P is the fluid pressure."

Computing the quantity $ln\left(\frac{1}{q[T]} - 1\right)$ and comparing with inverse temperature $(\frac{1}{T})$ yields a linearization:

$$ln\left(\frac{1}{q[T]} - 1\right) = -\frac{\Delta H}{R}\frac{1}{T} + ln(PK_{\mathrm{o}}) \qquad \textbf{Eq. S14-5}$$

Here, the negative slope is proportional to the effective enthalpy of adsorption of water (ΔH) in the confined geometry, which is a thermodynamic property of the confined water molecules (**Supplementary Fig. 14-3**).

c) Substrate-Supported Interior Filling CNTs Replicate a Positive Second Derivative

CNTs synthesized identically to the platform described in **Fig. 1** in the main text, except for the absence of the TEM window in the substrate can be made to replicate the RBM trajectories assigned to interior water filling, but not the strain dependent coupling. Similar fluid isobars and fluid phase transitions are observed when water is present within FIB-segmented, substrate-bound CNTs (**Supplementary Fig. 14-4a** and **b**). The exposure of fluid after FIB milling results in the filling of certain CNT segments, which is indicated by a characteristic upshift in the RBM frequency (**Supplementary Fig. 14-4c**). Note that prior to segmentation, the RBMs of as-grown, substrate-bound CNTs typically do not respond to liquid water immersion (**Supplementary Text 1: a**). This phenomenon attests the presence of saturated vdW interactions with the environmental coupling, and indicate that as-grown CNTs are impermeable to fluid filling. The filled CNT segment (Segment 1) displays reversible RBM trajectories with temperature cycles, in contrast, the RBM of the control (empty) CNT segment (Segment 2) does not show a distinct temperature dependence. Efficient laser heating of substrate-bound CNTs often presents challenges due to their better thermal coupling to the environment compared to partially suspended CNTs. Consequently, to measure the interior-fluid phase transitions occurring with a substrate-bound CNT segment requires a suitable spot along the CNT length where laser heating is both sufficient and efficient. These substrate-supported CNTs immersed in water environment do not exhibit the concave down trajectory or characteristic T_{max} associated with the strain-dependent coupling mechanism. This observation provides further evidence that the substrate-free suspension and self-tensioning of CNTs appear to be essential elements of that mechanism. Upon interior filling, the temperature dependent RBM trajectory, if observed, is concave up in the high temperature

range (**Supplementary Fig. 14-4d**), similar to the isobar trajectories above, and can be described successfully using the linearized Langmuir isobar equation (**Supplementary Fig. 14-4, e**).

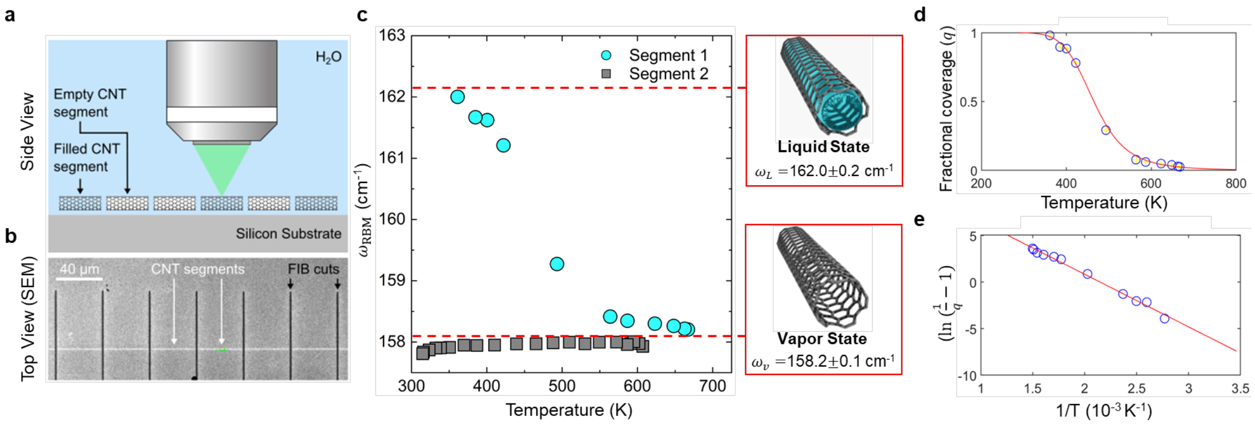

Supplementary Fig. 14-4| Observation of reversible fluid isobar of water-immersed, FIB-cut opened CNT. **a**, Schematic image of segmented, substrate-supported CNT in water environment and temperature scans by micro-Raman spectroscopy. **b**, SEM image of a segmented (40 µm each), substrate-supported CNT. **c**, Interior fluid isobars: RBM displays a reversible softening with temperature on the water-filled segment (Segment 1) only, not on the control (empty) segment (Segment 2). **d**, The experimental data (blue circles) were fitted to the Langmuir isobar adsorption as shown in the red curves, and **e**, exhibit a reasonable fit in the linearized form indicated in the red lines.

Supplementary References

1 Longhurst, M. & Quirke, N. Pressure dependence of the radial breathing mode of carbon nanotubes: the effect of fluid adsorption. *Phys. Rev. Lett.* **98**, 145503 (2007).

2 Chiashi, S. *et al.* Adsorption effects on radial breathing mode of single-walled carbon nanotubes. *Phys. Rev. B* **91**, 155415 (2015).

3 Kumar, R., Aykol, M. & Cronin, S. B. Effect of nanotube-nanotube coupling on the radial breathing mode of carbon nanotubes. *Phys. Rev. B* **78**, 165428 (2008).

4 Deng, L. *et al.* Coefficient of thermal expansion of carbon nanotubes measured by Raman spectroscopy. *Appl. Phys. Lett.* **104**, 051907 (2014).

5 Li, Z., Deng, L., Kinloch, I. A. & Young, R. J. Raman spectroscopy of carbon materials and their composites: Graphene, nanotubes and fibres. *Prog. Mater Sci.*, 101089 (2023).

6 Vollebregt, S., Ishihara, R., Tichelaar, F., Hou, Y. & Beenakker, C. Influence of the growth temperature on the first and second-order Raman band ratios and widths of carbon nanotubes and fibers. *Carbon* **50**, 3542-3554 (2012).

7 Wang, C., Ru, C. & Mioduchowski, A. Applicability and limitations of simplified elastic shell equations for carbon nanotubes. *J. Appl. Mech.* **71**, 622-631 (2004).

8 Liu, K. *et al.* Quantum-coupled radial-breathing oscillations in double-walled carbon nanotubes. *Nat. Commun.* **4**, 1375 (2013).

9 Girifalco, L. A., Hodak, M. & Lee, R. S. Carbon nanotubes, buckyballs, ropes, and a universal graphitic potential. *Phys. Rev. B* **62**, 13104-13110, doi:10.1103/PhysRevB.62.13104 (2000).

10 Lu, W. B. *et al.* A cohesive law for multi-wall carbon nanotubes. *Philos. Mag.* **87**, 2221-2232, doi:10.1080/14786430701344558 (2007).

11 Bellarosa, L., Bakalis, E., Melle-Franco, M. & Zerbetto, F. Interactions in Concentric Carbon Nanotubes: The Radius vs the Chirality Angle Contributions. *Nano Lett.* **6**, 1950-1954, doi:10.1021/nl061066g (2006).

12 Hirschmann, T. C. *et al.* Role of Intertube Interactions in Double- and Triple-Walled Carbon Nanotubes. *ACS Nano* **8**, 1330-1341, doi:10.1021/nn500420s (2014).

13 Gordeev, G., Wasserroth, S., Li, H., Flavel, B. & Reich, S. Moiré-Induced Vibrational Coupling in Double-Walled Carbon Nanotubes. *Nano Lett.* **21**, 6732-6739, doi:10.1021/acs.nanolett.1c00295 (2021).

14 Araujo, P. T. *et al.* Nature of the constant factor in the relation between radial breathing mode frequency and tube diameter for single-wall carbon nanotubes. *Phys. Rev. B* **77**, 241403, doi:10.1103/PhysRevB.77.241403 (2008).

15 Liu, Z., Gao, J., Zhang, G., Cheng, Y. & Zhang, Y.-W. From two-dimensional nano-sheets to roll-up structures: expanding the family of nanoscroll. *Nanotechnology* **28**, 385704, doi:10.1088/1361-6528/aa7bf8 (2017).

16 Kuehne, M. *et al.* Impedance of Thermal Conduction from Nanoconfined Water in Carbon Nanotube Single-Digit Nanopores. *J. Phys. Chem. C* **125**, 25717-25728, doi:10.1021/acs.jpcc.1c08146 (2021).

17 Hachtel, J. A. *et al.* Nanoscale vibrational spectroscopy and simulations of water under extreme confinement unveil diverse structural phases. *In Preparation* (2023).

18 Longhurst, M. J. & Quirke, N. The environmental effect on the radial breathing mode of carbon nanotubes in water. *J. Chem. Phys.* **124**, 234708, doi:10.1063/1.2205852 (2006).

19 Longhurst, M. J. & Quirke, N. The environmental effect on the radial breathing mode of carbon nanotubes. II. Shell model approximation for internally and externally adsorbed fluids. *J. Chem. Phys.* **125**, 184705, doi:10.1063/1.2360943 (2006).
