# Supplementary material for: Environmental damping and vibrational coupling of confined fluids within isolated carbon nanotubes
Source: Nat Commun. 2024 Jul 3;15:5605. doi: 10.1038/s41467-024-49661-8 (PMC11222464; doi:10.1038/s41467-024-49661-8)
Supplement: Supplementary file 4 — Description of Additional Supplementary Files [file 41467_2024_49661_MOESM4_ESM.pdf]

Description of Additional Supplementary Files

File Name: Supplementary Movie 1

Description: Using TEM videography, the diffusion of graphitic ribbons from the Mean Squared Displacement (MSD) tracking is observed to be approximately 0.12 nm^2 s^{-1}. This translates to a time span surpassing 10^6 s for them to reversibly leave and enter the 2 μm Raman laser spot (Supplementary Text 5: b, Supplementary Fig. 5-2).

File Name: Supplementary Movie 2

Description: A water-carbon oxidation product is observed and formed from the electron beam within the interior CNT after a water-vapor filling procedure.
